# Sex-specific associations between physical activity and cardiovascular disease under air pollution among the middle-aged and elderly population: A nationwide cross-sectional study in China

**DOI:** 10.1016/j.jnha.2025.100731

**Published:** 2025-11-21

**Authors:** Huilong Xie, Xuebing Sun, Liyu Huang, Tao Wang, Qi Yu, Min Hu, Jingwen Liao

**Affiliations:** a Guangdong Provincial Key Laboratory of Physical Activity and Health Promotion, Guangzhou Sport University, Guangzhou, China; b College of Urban and Environmental Sciences, Peking University, Beijing, China; c HSBC Business School, Peking University, Shenzhen, China

**Keywords:** Physical activity, Air pollution, Cardiovascular diseases, Middle-aged and elderly people

## Abstract

**Introduction:**

Moderate-vigorous physical activity (MVPA) is known to reduce the risk of cardiovascular disease (CVD), but its role in mitigating or exacerbating this risk under exposure to air pollution remains unclear. This study investigates the associations between MVPA and CVD in the context of air pollution among both sexes.

**Methods:**

This nationwide, cross-sectional study included 17,138 middle-aged and elderly people from the 2018 wave of the China Health and Retirement Longitudinal Study. Physical activity was assessed using the International Physical Activity Questionnaire. Data on air pollutants, including particulate matter, sulfur dioxide, ozone, nitrogen dioxide, carbon monoxide, and air quality index, were obtained from the China High Air Pollutants dataset and the Science Data Bank. CVD was identified based on self-reported physician diagnoses of heart disease or stroke.

**Results:**

More physically active participants were associated with lower CVD prevalence, while all air pollutants were linked to higher prevalence in the top exposure quartile. Physically inactive individuals exhibited the highest CVD prevalence across different levels of air pollution. Under high levels of air pollution, MVPA generally exhibited an inverse association with CVD prevalence in males (P for overall < 0.05), but not in females (P for overall > 0.05). In addition, MVPA duration positively mediated the associations between air pollutant exposure and CVD prevalence, with mediation proportions ranging from 10.10 to 33.30% in females and 5.14 to 7.53% in males.

**Conclusions:**

This study highlights that, under high levels of air pollution, the inverse association between MVPA and CVD prevalence was generally observed among males. Moreover, MVPA mediates the relationships between all air pollutants and CVD prevalence.

## 1 Introduction

As a leading cause of prevalence, disability, and premature mortality in the world, cardiovascular diseases (CVD) significantly undermine individual well-being, social functioning, and economic productivity [1,2]. Middle-aged and older adults are particularly vulnerable, owing to age-related declines in cardiovascular function, diminished motor abilities and widespread environmental hazards [[3], [4], [5]]. Both physical activity and environmental exposures are known to play pivotal roles in shaping cardiovascular health [6,7]. Physical activity has been verified as a protective factor for CVD, while exposure to air pollution has been proved to possess harmful effects on CVD [8,9]. Evidence indicates that approximately 99% of the global population is exposed to air quality levels that fall below the health standards established by the World Health Organization (WHO) [10]. Air pollution may be linked to approximately 8.34 million deaths globally, which substantially surpasses earlier projections, underscoring the escalating public health burden of environmental pollution [11]. People experiencing the burden of CVD would be driven by fine particulate matter (PM), carbon monoxide (CO), sulfur dioxide (SO_2_), nitrogen dioxide (NO_2_), ozone (O_3_) [[12], [13], [14], [15], [16]]. Given increasing concerns about air pollution, recent studies have sought to determine whether the cardioprotective effects of physical activity are modified or attenuated by exposure to air pollution [17].

Therefore, it is essential to jointly consider individuals’ physical activity and the ambient pollution levels in their regions when examining the association and impact on CVD. However, current research findings on this topic remain inconsistent. Several large-scale epidemiological studies have demonstrated that the protective effects of physical activity on atherosclerotic CVD risk, cardiovascular prevalence, cardiovascular mortality, and type 2 diabetes incidence persisted even across varying levels of air pollution [[18], [19], [20], [21], [22]]. Other studies reported that exposure to elevated concentrations of PM and NO_2_ might attenuate these benefits, particularly with respect to blood pressure regulation and metabolic syndrome [[23], [24], [25]]. Moreover, emerging studies suggest that more physical activity under high levels of PM and O_3_ may exert detrimental effects on cardiovascular health [[26], [27], [28]]. In general, the association between physical activity and CVD prevalence may vary by air pollution levels, whereas many studies neglect the linear expose-response relationship after stratifying pollutant concentrations. Additionally, given the considerable spatial heterogeneity in both the composition and concentration of air pollution, there is a critical need for more comprehensive and integrative air pollution indicators in future research. So far, although sexual differences in the aging process, CVD progression, and the cardiovascular response to physical activity have been observed [[29], [30], [31]], limited evidence based on sex-stratified analyses has restricted the ability to clarify the inconsistent associations of physical activity and air pollution with CVD outcomes.

To address these knowledge gaps, we stratified a nationally representative middle aged and elderly population sample of China by sex and assessed the associations of physical activity, comprehensive air pollution, and the prevalence of CVD. We examined both the independent and joint associations of physical activity and air pollution with CVD prevalence, evaluated their interaction and expose–response relationships, and explored the mediating role of physical activity in the link between air pollution and CVD prevalence.

## 2 Material and methods

### 2.1 Study population

The China Health and Retirement Longitudinal Study (CHARLS) is a nationally representative cohort survey of adults aged ≥45 years in China (https://charls.pku.edu.cn/). Using multistage, stratified, and probability-proportional-to-size sampling, it covers 150 counties/districts and 450 communities across 28 provinces. Full details of the study description were described elsewhere [32]. In brief, it adopted a multistage, stratified, probability-proportional-to-size (PPS) sampling design. All surveyed counties and districts were stratified by region, urban–rural status, and per capita Gross Domestic Product. Based on population size, 150 counties or districts were selected using PPS sampling, where the first sampling unit was randomly chosen and subsequent units were determined by adding a fixed interval (*n* = N/150) to ensure proportional representation. The study was performed in accordance with the principles of the Declaration of Helsinki and was approved by the Institutional Review Board of Peking University (IRB00001052-11015).

In this current study, we used data sourced from the 2018 wave of the CHARLS, which provided comprehensive information on physical activity across all participants. After excluding participants with incomplete data pertaining to CVD, physical activity, or critical covariates, as well as those under the age of 45, a definitive sample of 17,138 participants was included for the final analysis. The process of participant selection is depicted in Supplemental Fig. 1.

### 2.2 Self-reported physical activity

Physical activity data were obtained through a standardized instrument adapted from the International Physical Activity Questionnaire (IPAQ) [33]. Participants reported the weekly frequency (days/week), duration, and intensity (categorized as vigorous, moderate, or light) of physical activity. To quantify weekly physical activity duration, daily activity time was first grouped into five categories: <10, 10–29, 30–119, 120–239, and ≥240 min. Median duration was assigned to each category to facilitate estimation, with durations of 0 and 240 min applied to the lowest and highest groups, respectively, in accordance with prior studies [34]. Total weekly duration was then calculated by multiplying frequency by the assigned median duration. Responses that could not be logically assigned to any of the predefined duration categories were coded as missing. Additionally, participants who provided incomplete data on any key aspect of physical activity (including frequency, duration, or intensity) were excluded. Because light physical activity may not provide the equivalent health benefits as moderate-vigorous physical activity (MVPA), we quantified physical activity duration by aggregating the accumulated time spent in MVPA [35,36].

Physical activity intensity was subsequently expressed in metabolic equivalent tasks (METs): 8.0 METs for vigorous, 4.0 for moderate, and 3.3 for mild activity [37,38]. The total physical activity volume (MET-minutes/week) was computed as: (8.0 × time of vigorous physical activity) + (4.0 × time of moderate physical activity) + (3.3 × time of mild physical activity). Based on the WHO Guidelines on Physical Activity and Sedentary Behaviour and the IPAQ, inactive was considered as not participating in MVPA. And we use the same METs as 300 min of moderate physical activity or 150 min of vigorous physical activity as the dividing line between insufficiently active and physically active [33,39].

### 2.3 Evaluation of air pollution

Air pollution including PM_1_, PM_2.5_, PM_10_, CO, SO_2_, NO_2_, O_3_, and air quality index (AQI) were retrieved from the China High Air Pollutants dataset (https://weijing-rs.github.io/product.html) and Science Data Bank (https://www.scidb.cn/en). Details regarding the estimation of air pollution exposure have been described extensively in previous studies [[40], [41], [42], [43], [44], [45], [46]]. Briefly, air pollution exposure was estimated using space–time extremely randomized trees models, incorporating a range of spatial and temporal predictors, including meteorological conditions, land use characteristics, pollutant emission sources, and population density. Daily concentrations of PM_1_, PM_2.5_, PM_10_, and O_3_ were predicted at a 1 km × 1 km resolution, whereas CO, SO_2_, and NO_2_ were modeled at a 10 km × 10 km resolution. Due to the city-level geocoding of participant locations in CHARLS, individual air pollution exposure was assigned using city-level average concentrations. The AQI is a numerical indicator used to assess air quality, based on the concentrations of multiple pollutants, including CO, PM_2.5_, SO_2_, NO_2_, O_3_, and PM_10_. Following established methodology, air pollution exposure for each participant was assigned based on the average concentrations during the one-year period preceding the interview [34,38].

### 2.4 Cardiovascular diseases

The primary outcome of this study was the prevalence of CVD, defined as the presence of heart diseases (including angina, heart attack, congestive heart failure, and other heart problems) or stroke. In accordance with established precedents, participants were inquired whether they were diagnosed with heart disease or stroke by physician [47].

### 2.5 Assessment of covariates

The covariates included demographic characteristics (age, sex, area of residence, and marital status), socioeconomic status (education level and household expenditure per capita), and health and behavioral factors (smoke, drink, solid fuel usage, sleep duration, depression symptoms, and non-communicable diseases). Demographic characteristics included age, sex (female, male), area of residence (rural, urban), and marital status (married or partner, others). Socioeconomic status was represented by education level (primary school and below, middle school, high school and above) and household expenditure per capita (low, moderate, high), which can better symbolize the real economic status in China [48]. Health and behavioral factors included smoke (no, yes), drink (no, yes), solid fuel usage (no, yes), sleep duration (<7 h, ≥7 h), depressive symptoms (no, yes), and non-communicable diseases (no, yes).

Non-communicable diseases were identified based on self-reported physician diagnoses of any of the following conditions: hypertension, dyslipidemia, diabetes, cancer, lung disease, liver disease, kidney disease, digestive disorders, psychiatric problems, arthritis, or asthma. The 10-item version of the Center for Epidemiological Studies Depression Scale, a validated and widely applied instrument among Chinese adults, was used to assess depressive symptoms [49,50]. Respondents reported the frequency of specific emotional and psychological experiences over the past week, including difficulty concentrating, feeling depressed, restless sleep, fearfulness, loneliness, a lack of motivation, and the perception that everything required significant effort. Positive effect items, such as feelings of hopefulness and happiness, were reverse-coded. Each response was scored from 0 to 3, corresponding to symptom frequency: <1 day (0), 1–2 days (1), 3–4 days (2), and 5–7 days (3). Total scores, ranging from 0 to 30, were calculated by summing all items, with higher scores indicating more severe depressive symptoms. A threshold of ≥10 was applied to recognize participants with notable depressive symptoms [51].

### 2.6 Statistical analysis

Due to biological differences in aging process, CVD progression, and the cardiovascular response to physical activity [[29], [30], [31]], all analyses were conducted in a sex-specific manner. Specifically, we conducted analyses to describe or examine the associations in the total population, females, and males separately. Sex classification in this study was derived from the sex identifier recorded in the cohort’s registration database.

First, descriptive statistics were presented as mean (standard deviation), numbers of participants (percentages), or median (25th, 75th percentiles) by sex. Then, multivariable logistic regression models were performed to evaluate the associations of physical activity with the prevalence of CVD and air pollution with the prevalence of CVD in the total sample and in sex-specific subgroups. The first multivariable model was adjusted for sex (in the total sample only), age, area of residence, marital status, and household expenditure. Smoke, drink, solid fuel usage, and sleep duration were further adjusted in the second multivariable model. A third model (fully adjusted model) was additionally adjusted for depression symptoms and non-communicable diseases. Odds ratios (ORs) with 95% confidence intervals (CIs) and P for trend were estimated. Collinearity among covariates was assessed using a correlation matrix, which indicated no significant multicollinearity.

Jointly analyses were conducted by categorizing air pollution into quartiles and MVPA into three levels (inactive, insufficiently active, and physically active), compared to participants who were physically active under the lowest quartile of air pollution. Subgroup analysis on the associations between high levels of air pollution and the prevalence of CVD stratified by MVPA categories. Additionally, the dose-response associations between MVPA duration and the prevalence of CVD were stratified by the levels of air pollution, using restricted cubic splines fitted in the fully adjusted logistics models.

We further performed a mediation analysis to explore the mediation role of MVPA in the association between air pollution and the prevalence of CVD. Within this framework, a full adjusted logistic regression model was specified to assess the outcome, incorporating both the primary exposures—air pollution—as well as the mediator variable, defined by MVPA duration. The extent of mediation was quantified by calculating the proportion of the indirect effect relative to the total effect, expressed as the mediation percentage. The statistical significance of this mediating pathway was evaluated via bootstrap resampling (times = 1000) [52]. Furthermore, we applied generalized additive models to examine the potential nonlinear associations between air pollution and MVPA. All statistical analyses were conducted using R software (version 4.3.3). Two-sided tests were applied throughout, with statistical significance defined as P value <0.05.

### 2.7 Sensitive analyses

We performed a sensitivity analysis to assess the robustness of our findings. First, we adjusted for additional covariates including the installation of residential air cleaner and household temperature. Second, we use the average concentration of the last follow-up year and the 3-year lag year to estimate air pollution concentration. Third, Individual weights were further adjusted.

## 3 Results

### 3.1 Participants characteristics

Table 1 presents the characteristics of the 17,138 participants included in the study. The mean age was 61.17 years. Among the total sample, 8945 participants (52.2%) were female and 8193 (47.8%) were male. A total of 4123 participants (24.1%) had a diagnosis of CVD. According to the WHO guidelines, 6426 participants (37.5%) were classified as inactive, 1749 (10.2%) as insufficiently active, and 8963 (52.3%) as physically active, with significant difference between sexes (*P* < 0.001).

**Table 1:** Participant characteristics based on CHARLS 2018.

Characteristics	Total	Female	Male	P value^a^
Number of participants	17,138	8945	8193	
Age (years)	61.17 (9.63)	60.81 (9.67)	61.57 (9.57)	<0.001
Residence (rural, %)	10,359 (60.4)	5358 (59.9)	5001 (61.0)	0.131
Education				<0.001
Primary school and below	11,778 (68.7)	6859 (76.7)	4919 (60.0)	
Middle school	3373 (19.7)	1359 (15.2)	2014 (24.6)	
High school and above	1987 (11.6)	727 (8.1)	1260 (15.4)	
Marital status (Married or partnered, %)	14,807 (86.4)	7394 (82.7)	7413 (90.5)	<0.001
Household expenses per capita				0.115
Low	5713 (33.3)	3045 (34.0)	2668 (32.6)	
Moderate	5713 (33.3)	2960 (33.1)	2753 (33.6)	
High	5712 (33.3)	2940 (32.9)	2772 (33.8)	
Smoke (yes, %)	7325 (42.7)	690 (7.7)	6635 (81.0)	<0.001
Drink (yes, %)	8237 (48.1)	2077 (23.2)	6160 (75.2)	<0.001
Solid fuel usage (yes, %)	5920 (34.5)	3072 (34.3)	2848 (34.8)	0.576
Sleep duration (<7 h/day)	7737 (45.1)	4490 (50.2)	3247 (39.6)	<0.001
Depressive symptom (yes, %)	6333 (37.0)	3906 (43.7)	2427 (29.6)	<0.001
NCDs (yes, %)	13,423 (78.3)	7074 (79.1)	6349 (77.5)	0.012
CVD (yes, %)	4123 (24.1)	2376 (26.6)	1747 (21.3)	<0.001
MVPA (hours/day)	0.85 [0.00, 2.99]	0.85 [0.00, 2.99]	0.89 [0.00, 3.52]	0.034
WHO physical activity guideline^b^				<0.001
Inactive	6426 (37.5)	3284 (36.7)	3142 (38.3)	
Insufficiently active	1749 (10.2)	1035 (11.6)	714 (8.7)	
Physically active	8963 (52.3)	4626 (51.7)	4337 (52.9)	
Air pollutants (μg/m^3^)				
CO	0.92 [0.81, 1.08]	0.92 [0.81, 1.08]	0.92 [0.81, 1.08]	0.489
PM_1_	23.26 [18.78, 29.92]	23.25 [18.78, 29.92]	23.26 [18.78, 29.92]	0.672
PM_2.5_	40.94 [33.31, 54.42]	40.94 [33.31, 54.42]	40.96 [33.31, 55.13]	0.595
SO_2_	15.48 [12.63, 21.29]	15.46 [12.63, 21.29]	15.48 [12.63, 21.45]	0.531
NO_2_	26.84 [20.56, 35.29]	26.84 [20.56, 35.29]	26.84 [20.92, 35.29]	0.739
O_3_	95.86 [88.32, 105.92]	95.86 [88.32, 105.92]	95.86 [88.32, 105.92]	0.341
PM_10_	71.25 [53.60, 100.88]	71.25 [53.60, 100.88]	72.06 [53.60, 100.88]	0.637
AQI^c^	81.65 [68.14, 104.02]	81.65 [68.14, 102.12]	81.65 [68.14, 104.02]	0.618

Continuous covariates were expressed as mean (standard deviation). Continuous exposure variables were expressed as median (25th, 75th percentile). Categorical variables were expressed as number (percentage).

a Variables between groups were compared by t test, Kruskal-Wallis test, or Chi-square test. P < 0.05 was considered statistically significant.

b Based on the World Health Organization Guidelines on Physical Activity and Sedentary Behaviour 2020 and the International Physical Activity Questionnaire, inactive was considered as not participating moderate-vigorous physical activity. And we use the same METs as 300 min of moderate physical activity or 150 min of vigorous physical activity as the dividing line between insufficiently active and physically active.

c The AQI is a numerical indicator used to assess air quality, based on the concentrations of multiple pollutants, including CO, PM_2.5_, SO_2_, NO_2_, O_3_, and PM_10_. NCDs, non-communicable diseases; CVD, cardiovascular disease; MVPA, moderate-vigorous physical activity; WHO, World Health Organization; CO, Carbon monoxide; PM_1_, particulate matter with aerodynamic diameter ≤1 μm; PM_2.5_, particulate matter with aerodynamic diameter ≤2.5 μm; SO_2_, Sulfur dioxide; NO_2_, nitrogen dioxide; O_3_, Ozone; PM_10_, particulate matter with aerodynamic diameter ≤10 μm; AQI, air quality index.

### 3.2 Independent associations of MVPA and air pollution with the prevalence of CVD

After full adjustment, each additional 0.5 h/day of MVPA was associated with lower odds of CVD (OR (95% CI) = 0.95 (0.94−0.96)), with similar associations observed among females (OR (95% CI) = 0.95 (0.94−0.96)) and males (OR (95% CI) = 0.95 (0.93−0.96)) (Supplemental Tables 1–3). Compared with inactive individuals, both the insufficiently active (OR (95% CI) = 0.81 (0.71−0.92)) and the sufficiently active (OR (95% CI) = 0.66 (0.61−0.72)) were associated with lower odds of CVD. For air pollutants (Supplemental Tables 4–6), each 10-unit increase in AQI was associated with a higher prevalence of CVD (OR (95% CI) = 1.09 (1.07–1.11)); the highest prevalence observed in the top exposure quartile of AQI (OR (95% CI) = 1.55 (1.40–1.72)) than the lowest quartile. Comparable associations were observed for other air pollutants, including each 0.1-unit increase in CO (OR (95% CI) = 1.05 (1.03–1.06)), each 5-unit increase in PM_1_ (OR (95% CI) = 1.07 (1.05–1.10)), each 5-unit increase in PM_2.5_ (OR (95% CI) = 1.05 (1.04–1.07)), each 5-unit increase in SO_2_ (OR (95% CI) = 1.11 (1.09–1.14)), each 5-unit increase in NO_2_ (OR (95% CI) = 1.05 (1.03–1.07)), each 10-unit increase in O_3_ (OR (95% CI) = 1.11 (1.07–1.15)), and each 10-unit increase in PM_10_ (OR (95% CI) = 1.08 (1.07–1.10)). Similarly, the highest odds were found in the top exposure quartile for CO (OR (95% CI) = 1.45 (1.31–1.61)), PM_1_ (OR (95% CI) = 1.61 (1.45–1.78)), PM_2.5_ (OR (95% CI) = 1.73 (1.55–1.92)), SO_2_ (OR (95% CI) = 1.54 (1.39–1.71)), NO_2_ (OR (95% CI) = 1.28 (1.15–1.42)), O_3_ (OR (95% CI) = 1.36 (1.22–1.51)), and PM_10_ (OR (95% CI) = 2.01 (1.81–2.24)).

### 3.3 Joint associations of MVPA and air pollution with the prevalence of CVD

**Fig. 1:**
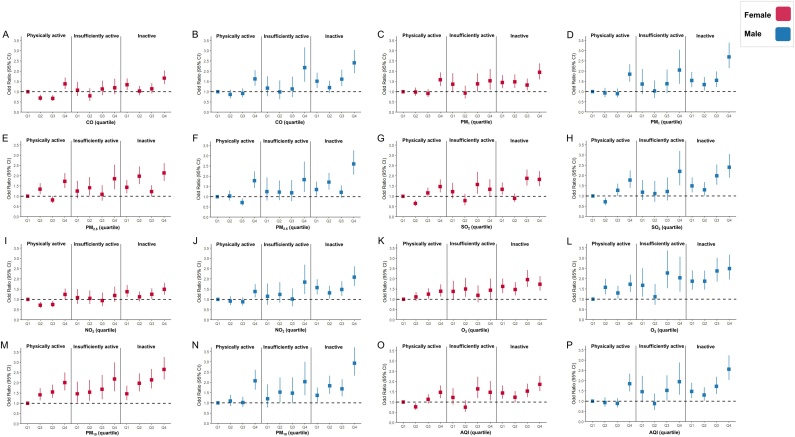
Joint association of MVPA and air pollutants with the prevalence of CVD, CHARLS 2018. Joint association of MVPA (WHO Guidelines on Physical Activity and Sedentary Behaviour 2020) and air pollutants (quartile) with the prevalence of CVD within each subgroup: female (A, C, E, G, I, K, M, and O) and male (B, D, F, H, J, L, N, and P). Based on the WHO guidelines, inactive was considered as not participating MVPA. And we use the same metabolic equivalents as 300 min of moderate physical activity or 150 min of vigorous physical activity as the dividing line between insufficiently active and physically active. Multivariable logistic regression model was used to examine the associations, which were adjusted for age, education, residence, marital status, household expenses per capita, smoke, drink, solid fuel usage, sleep duration, depressive symptoms, and non-communicable diseases. CVD, cardiovascular diseases; MVPA, moderate-vigorous physical activity; WHO, World Health Organization; CI, confidence interval; CO, carbon monoxide; PM_1_, particulate matter with aerodynamic diameter ≤1 μm; PM_2.5_, particulate matter with aerodynamic diameter ≤2.5 μm; SO_2_, sulfur dioxide; NO_2_, nitrogen dioxide; O_3_, ozone; PM_10_, particulate matter with aerodynamic diameter ≤10 μm; AQI, air quality index.

In the joint analyses (Fig. 1 and Supplemental Tables 7–9), physically active, insufficient active, and inactive people living under the worst quartile of AQI represent higher odds of CVD (OR (95% CI) = 1.62 (1.38–1.89), (OR (95% CI) = 1.66 (1.30–2.13)), and (OR (95% CI) = 2.11 (1.82–2.46)) as compared with physically active individuals under the lowest quartile of air pollution. Notably, inactive individuals in the highest quartile of air-pollutant exposure exhibited the highest odds of CVD prevalence across all joint groups; comparable results were observed across sexes and most air pollution.

### 3.4 Stratified associations (by MVPA) between high levels of air pollution and the prevalence of CVD

**Fig. 2:**
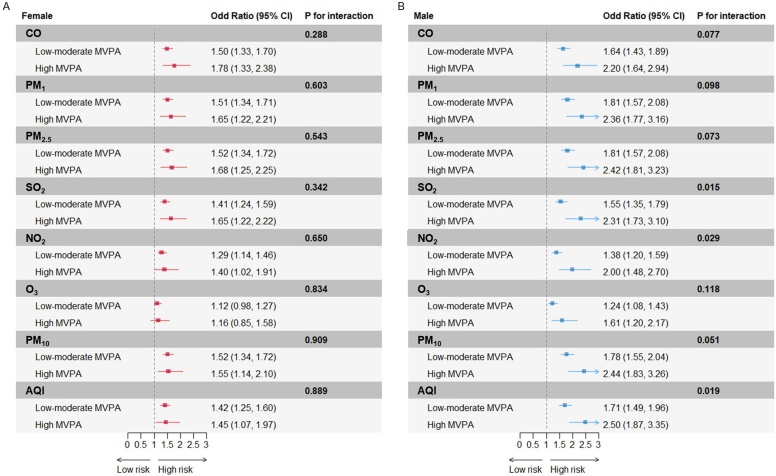
Subgroup analysis on the associations between high levels of air pollutants and the prevalence of CVD stratified by MVPA categories, CHARLS 2018. Interaction association of MVPA (high and low-medium) and air pollutants (high and low-medium) with the prevalence of CVD within each subgroup: (A) female and (B) male. The cut off point of air pollutants and MVPA are cohort-specific cut-off points at the 75%. Multivariable logistic regression model was used to examine the associations, which were adjusted for age, education, residence, marital status, household expenses per capita, smoke, drink, solid fuel usage, sleep duration, depressive symptoms, and non-communicable diseases. CVD, cardiovascular diseases; MVPA, moderate-vigorous physical activity; CI, confidence interval; CO, carbon monoxide; PM_1_, particulate matter with aerodynamic diameter ≤1 μm; PM_2.5_, particulate matter with aerodynamic diameter ≤2.5 μm; SO_2_, sulfur dioxide; NO_2_, nitrogen dioxide; O_3_, ozone; PM_10_, particulate matter with aerodynamic diameter ≤10 μm; AQI, air quality index.

Fig. 2 illustrates the associations of high levels of air pollution and CVD prevalence within each strata of MVPA (categorized as high and low-moderate) (Supplemental Tables 10–12). Although the associations generally tended to be higher in individuals with high MVPA levels, MVPA significantly modified the relationship between air pollution and CVD prevalence only among males (P for interaction = 0.015 for SO_2_, 0.029 for NO_2_, 0.019 for AQI).

### 3.5 Stratified associations (by air pollution) between MVPA and the prevalence of CVD

**Fig. 3:**
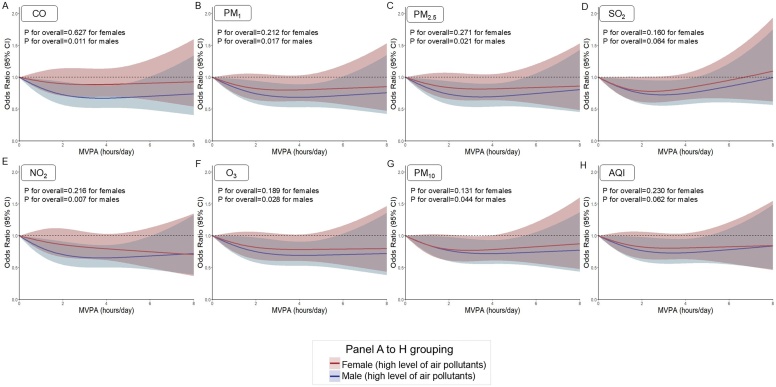
Dose–response curves of the associations of MVPA with the prevalence of CVD stratified by air pollutants concentration, CHARLS 2018. The cut off point of air pollutants is cohort-specific cut-off points at the 75%. The panel depicts adjusted restricted cubic splines with 95% confidence. All models were adjusted for age, education, residence, marital status, household expenses per capita, smoke, drink, solid fuel usage, sleep duration, depressive symptoms, and non-communicable diseases. CVD, cardiovascular diseases; MVPA, moderate-vigorous physical activity; CI, confidence interval; CO, carbon monoxide; PM_1_, particulate matter with aerodynamic diameter ≤1 μm; PM_2.5_, particulate matter with aerodynamic diameter ≤2.5 μm; SO_2_, sulfur dioxide; NO_2_, nitrogen dioxide; O_3_, ozone; PM_10_, particulate matter with aerodynamic diameter ≤10 μm; AQI, air quality index.

Fig. 3 and Supplemental Fig. 2 represent the associations between MVPA and CVD prevalence within each strata of air pollution (categorized as high and low-moderate). Under high levels of air pollution, MVPA generally maintained a significant inverse association with CVD prevalence in males except for SO_2_, whereas no significant associations were found in females across all air pollution (P for overall>0.05 for females, P for overall<0.05 for males) (Supplemental Tables 13–15). At low levels of air pollution, MVPA was significantly inversely associated with CVD prevalence in both sexes (P for overall < 0.001) (Supplemental Figs. 2, 3 and Supplemental Tables 13–15). Notably, MET-defined physical activity was consistently associated with lower odds of CVD prevalence among males (P for overall < 0.05), but not among females, under high levels of all air pollutants (P for overall > 0.05) (Supplemental Fig. 4 and Supplemental tables 13–15).

### 3.6 Mediation effects of MVPA on the associations between air pollution and the prevalence of CVD

**Fig. 4:**
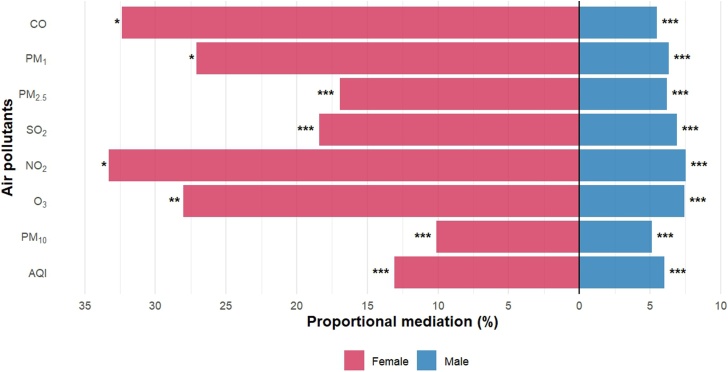
Proportional mediation of the data example in which MVPA is hypothesized as a mediator of the relation between air pollutants and the prevalence of CVD, CHARLS 2018. * *P* < 0.05, ** *P* < 0.01, *** *P* < 0.001. All Models were adjusted for age, education, residence, marital status, household expenses per capita, smoke, drink, solid fuel usage, sleep duration, depressive symptoms, and non-communicable diseases. CVD, cardiovascular diseases; MVPA, moderate-vigorous physical activity; CI, confidence interval; CO, carbon monoxide; PM_1_, particulate matter with aerodynamic diameter ≤1 μm; PM_2.5_, particulate matter with aerodynamic diameter ≤2.5 μm; SO_2_, sulfur dioxide; NO_2_, nitrogen dioxide; O_3_, ozone; PM_10_, particulate matter with aerodynamic diameter ≤10 μm; AQI, air quality index.

MVPA exhibited significant mediating effects on the associations between all air pollution and CVD prevalence (*P* < 0.001) (Fig. 4 and Supplemental Tables 16–18). All air pollution exhibited significant nonlinear associations with MVPA (P for nonlinear<0.001) (Supplemental Figs. 5–7). Among females, MVPA mediated 32.38%, 27.10%, 16.96%, 18.39%, 33.30%, 28.00%, 10.10% and 13.10% of the associations between CVD prevalence when exposure to CO, PM_1_, PM_2.5_, SO_2_, NO_2_, O_3_, PM_10_, and AQI, respectively. While among males, MVPA mediated 5.49%, 6.32%, 6.18%, 6.92%, 7.53%, 7.44%, 5.14%, and 6.02% of those associations, respectively. Notably, in females, the effects of CO, PM_1_, NO_2_, and O_3_ on CVD prevalence were not significant directly (*P* > 0.05), whereas in males, the direct effects were also significant (*P* < 0.001).

### 3.7 Sensitive analyses

The results remained generally consistent with the primary analysis after additionally adjustment for the installation of residential air cleaner and household temperature, with only slight variations in effect estimates (Supplemental Tables 19–30). Similar findings were also observed when using 3-year lagged annual average concentrations of air pollution (Supplemental Tables 31–42) and after further adjustment for individual weights (Supplemental Tables 43–54).

## 4 Discussion

In this Chinese national representative survey among middle aged and elderly population, we evaluated the associations between MVPA levels and air pollution with CVD prevalence by sexes through a cross-sectional analytical design. MVPA duration was inversely associated with CVD prevalence, while the levels of air pollution were positively associated with CVD prevalence. When considering both MVPA and air pollution, individuals with inactive lifestyles under high levels of air pollution had the greatest CVD risk. More specifically, for people with either low-moderate or high MVPA, exposure to the top quartile of air pollution was generally associated with high risk of CVD for both sexes. On the other hand, for people living under lower levels of air pollution, there was an inverse association between MVPA and CVD prevalence, which appeared to be generally attenuated for males and absent for females under higher levels of air pollution. Additionally, MVPA showed a non-linear relationship with all air pollutants and may serve as a mediator in the association between air pollution and CVD prevalence in both sexes.

Jointly considering physical activity and air pollution is essential for accurately evaluating health outcomes, particularly in ambient outdoor spaces and among aging populations. Current evidence generally supports that, in settings of elevated ambient air pollution, physically active individuals might exhibit more adverse health outcomes [17,[53], [54], [55]]. However, data from longitudinal studies consistently demonstrate that adults with less frequency or lower volume of physical activity face the highest risk of CVD, even when exposed to elevated levels of PM_2.5_ and PM_10_ [19,21]. Accordingly, our findings also demonstrate that physically inactive individuals (based on 2020 WHO Guidelines on Physical Activity and Sedentary Behaviour [39]) show the largest prevalence of CVD among middle-aged and older Chinese adults in outdoor environments with increased levels of air pollution. These findings collectively suggest that that the physically inactive population may remain at the highest risk of CVD, even when exposed to higher levels of air pollution. In addition to PM_2.5_ and PM_10_, this current study also evaluated the joint associations between other air pollution (including CO, PM_1_, SO_2_, NO_2_, O_3_, and overall AQI) and physical activity with CVD prevalence with similar findings in both sexes.

To further visualized and validate the interactions identified in the joint analysis, stratifications in a sex-specific manner were performed to examine: how the association of MVPA with CVD prevalence across levels of air pollution, or how high/low air pollution with CVD across MVPA levels. Our study demonstrated that for people with either low-moderate or high MVPA, exposures to high levels of air pollution were associated with larger CVD prevalence, with interactive effects between MVPA and air pollution existed in males but not females. Similarly, prior research in women reported no significant multiplicative interaction between PM_2.5_ exposure and physical activity on CVD risk and overall mortality [20]. A critical reality is that most of the population on the earth are living in a highly polluted environment that exceeds the WHO air quality guidelines [56], therefore we classified air pollutant level using the highest risk category (above the 75th percentile) in the other stratified analysis. Our study found an inverse relationship between MVPA (within nearly 6 h/day) and CVD prevalence in males with statistical significance but not in females, indicating potential sex-specific differences. Although sexual dimorphisms in aging, CVD development, and the adaptations to physical activity had been documented [[29], [30], [31]], no prior study specifically assessed how physical activity volume related with CVD prevalence in females and males separately. To data, the available findings examined this issue are inconsistent: a South Korea longitudinal study of young adults aged 20–39 years found that transitioning to a more physically active lifestyle was associated with an increased incidence of CVD under high levels of PM_2.5_ and PM_10_ [57]; similarly, a longitudinal study in China of adults aged 30–79 years demonstrated that high levels of commuting- or farming-related physical activity were generally associated with higher incidence of CVD among farmers when PM_2.5_ exposure exceeded the median level but not in non-farmers [26]; another longitudinal study of 113,918 patients (≥40 years old) with dyslipidemia in South Korea found that transitioning to a more active lifestyle was not associated with CVD incidence at high levels of air pollution [58]. These disparate results may reflect differences in age, sex, or disease status of the recruited participants. To the best of our knowledge, this is the first sex-specified study among middle-aged and elderly population to explore the association between physical activity levels and CVD prevalence under high level of air pollution.

It is plausible that physical activity would modulate or attenuate the adverse cardiovascular effects of air pollution. At the physiological level, physical activity would increase breathing rate, tidal volume, and route of inhalation [59], which may influence pollutant deposition and subsequent cardiovascular responses. Mechanistically, animal studies have shown that physical activity can alleviate PM_2.5_-induced cardiovascular dysfunction by regulating lipid metabolism, coagulation, immune responses, and preserving endothelial function and nitric oxide bioavailability [60,61]. Exercise also mitigates oxidative stress and calcium handling disturbances in cardiomyocytes under CO exposure [62]. Behaviorally, individuals tend to reduce outdoor physical activity during high pollution periods [63], which, although protective in the short term, may increase overall cardiovascular risk due to physical inactivity, creating a feedback loop. Additionally, given the differing CVD risks between pre- and postmenopausal females, as well as the sex-specific differences in responses to air pollution, the underlying mechanisms by which females respond to air pollution and exercise across different hormonal phases remain unclear [64,65].

This study has several limitations that warrant consideration. First, the cross-sectional design limits causal inference between MVPA and CVD risk in polluted environments. Longitudinal data of the CHARLS cohort were not used due to substantial missingness in physical activity in earlier waves and pandemic-related disruptions in the later wave. Future longitudinal studies or clinical trials are needed to establish temporality and clarify potential causal pathways. Second, given the evolving environmental factors, regional heterogeneity, and variability in race, our findings may not be generalizable to other settings. Future studies leveraging more recent datasets and multicenter cohorts are needed to validate and extend these results. Third, the data did not specify between physical activity contexts (e.g., indoor and outdoor), which limits our ability to accurately analyze the relationship between physical activity and air pollution exposure. Moreover, physical activity was assessed using self-reported data via questionnaire; future research should incorporate objective activity measurements and context-specific data to better elucidate these associations.

## 5 Conclusions

Our findings indicate that all assessed concentration of air pollution were associated with an increased CVD prevalence, while longer durations of MVPA were linked to a decreased prevalence. Physically inactive individuals exhibited the highest CVD prevalence across the evaluated ranges of air pollution. More importantly, among males, engaging in MVPA was generally associated with a lower prevalence of CVD under high levels of air pollution, whereas this association was largely absent in females. Moreover, MVPA partially mediated the association between all air pollutants and CVD prevalence, with a numerically higher mediation proportion observed in females. These findings highlight the critical need to consider sex differences among middle-aged and elderly population when evaluating the complex interactions among MVPA, environmental exposures, and cardiovascular health, as well as when developing targeted public health interventions.

## Ethics approval and consent to participate

The CHARLS was approved by the Biomedical Ethics Committee of Peking University, and all participants were required to sign informed consent.

## Consent for publication

Not applicable.

## Declaration of Generative AI and AI-assisted technologies in the writing process

AI was not used in the preparation of this manuscript nor its table or figures.

## Funding sources

This work was supported by the 10.13039/501100003453Natural Science Foundation of Guangdong Province [grant numbers: 2025A1515012478], the Program for Key Research Areas of University in Guangdong Province [grant numbers: 2024ZDZX2062], the Special Funds for the Cultivation of Guangdong College Students’ Scientific and Technological lnnovation [grant numbers: 72301602 and 72400102], and the Guangzhou Basic Research Plan, Basic and Applied Basic Research Project [grant numbers: SL2023A04J00446].

## Data availability statement

The CHARLS database used in this study can be accessed by researchers on application (https://charls.pku.edu.cn/). Air pollution including PM_1_, PM_2.5_, PM_10_, CO, SO_2_, NO_2_, O_3_, and AQI were retrieved from the China High Air Pollutants dataset (https://weijing-rs.github.io/product.html) and Science Data Bank (https://www.scidb.cn/en).

## CRediT authorship contribution statement

**Huilong Xie:** Writing - original draft, Writing - review & editing, Software, Data curation, Validation, Methodology, Formal analysis. **Xuebing Sun:** Methodology, Formal analysis, Data curation. **Liyu Huang:** Methodology, Formal analysis, Software. **Tao Wang:** Software, Data curation, Methodology. **Qi Yu:** Data curation, Methodology. **Min Hu:** Conceptualization, Supervision. **Jingwen Liao:** Methodology, Writing - original draft, Writing - review & editing, Software, Conceptualization, Supervision.

## Declaration of competing interest

The authors declare that they have no known competing financial interests or personal relationships that could have appeared to influence the work reported in this paper.

## Glossary

CVD: Cardiovascular disease
MVPA: Moderate-vigorous physical activity
WHO: World Health Organization
PM: Fine particulate matter
CO: Carbon monoxide
SO_2_: Sulfur dioxide
NO_2_: Nitrogen dioxide
O_3_: Ozone
AQI: Air quality index
ORs: Odds ratios
Cis: Confidence intervals
