# Supplementary material for: Sex-specific associations between physical activity and cardiovascular disease under air pollution among the middle-aged and elderly population: A nationwide cross-sectional study in China
Source: J Nutr Health Aging. 2025 Nov 21;30(1):100731. doi: 10.1016/j.jnha.2025.100731 (PMC12681717; doi:10.1016/j.jnha.2025.100731)
Supplement: Supplementary file 1 [file mmc1.docx]

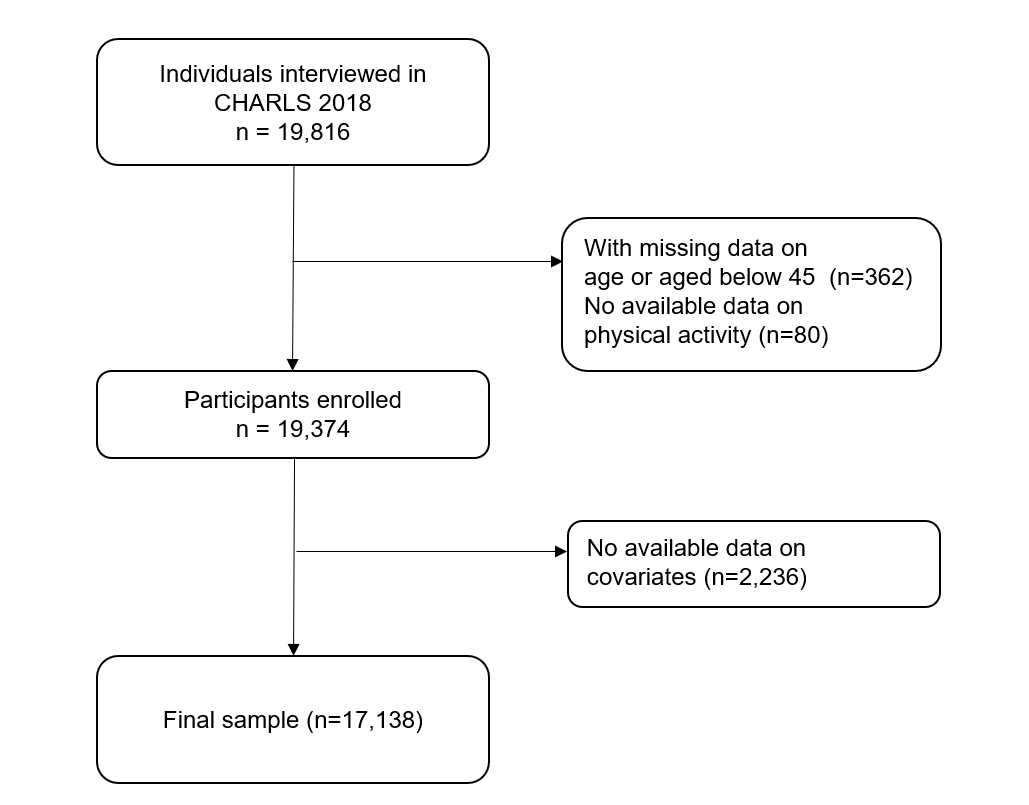


Supplemental figure 1. Flowchart of participant selection process


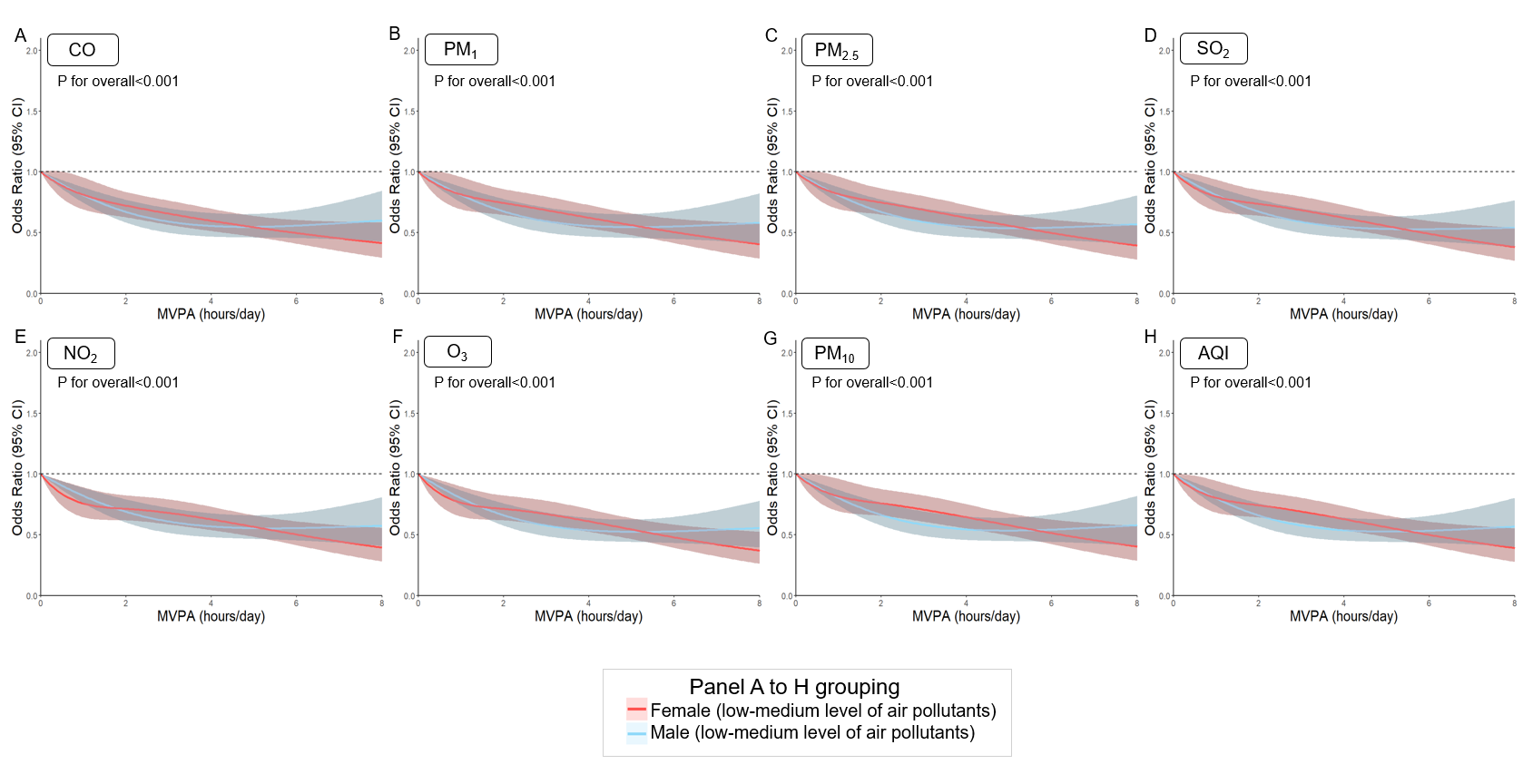


Supplementary figure 2. Dose–response curves of the associations of MVPA with the prevalence of CVD stratified by air pollutants concentration, CHARLS 2018. The cut off point of air pollutants is cohort-specific cut-off points at 75%. The panel depicts adjusted restricted cubic splines with 95% confidence. All models were adjusted for age, education, residence, marital status, household expenses per capita, smoke, drink, solid fuel usage, sleep duration, depressive symptoms, and non-communicable diseases. CVD, cardiovascular diseases; MVPA, moderate-vigorous physical activity; CO, carbon monoxide; PM_1_, particulate matter with aerodynamic diameter ≤1µm; PM_2.5_, particulate matter with aerodynamic diameter ≤2.5µm; SO_2_, sulfur dioxide; NO_2_, nitrogen dioxide; O_3_, ozone; PM_10_, particulate matter with aerodynamic diameter ≤10µm; AQI, air quality index.


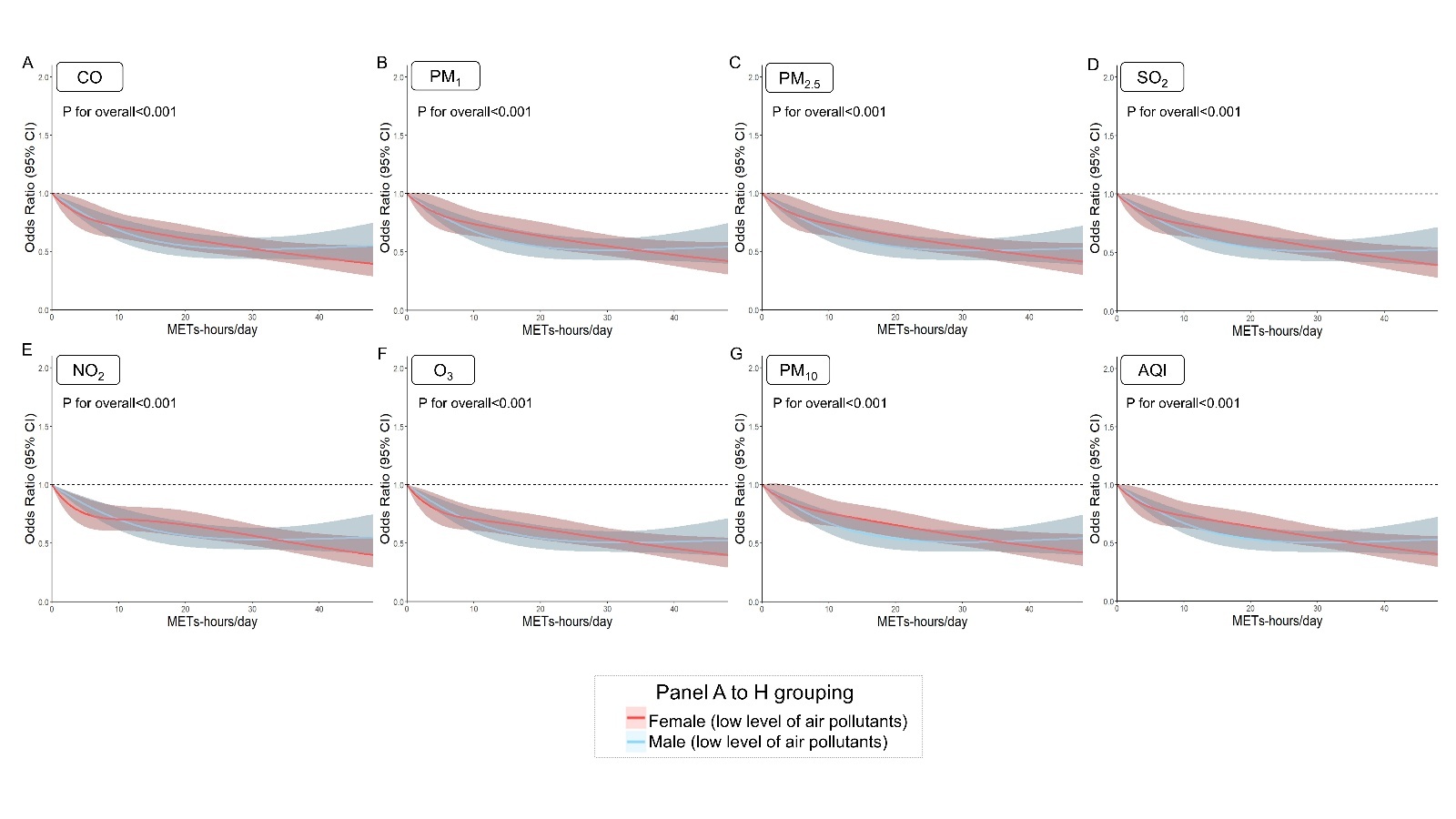


Supplementary figure 3. Dose–response curves of the associations of the METs of MVPA with the prevalence of CVD stratified by air pollutants concentration, CHARLS 2018. The cut off point of air pollutants is cohort-specific cut-off points at 75%. The panel depicts adjusted restricted cubic splines with 95% confidence. All models were adjusted for age, education, residence, marital status, household expenses per capita, smoke, drink, solid fuel usage, sleep duration, depressive symptoms, and non-communicable diseases. CVD, cardiovascular diseases; MVPA, moderate-vigorous physical activity; METs, Metabolic equivalents; CO, carbon monoxide; PM_1_, particulate matter with aerodynamic diameter ≤1µm; PM_2.5_, particulate matter with aerodynamic diameter ≤2.5µm; SO_2_, sulfur dioxide; NO_2_, nitrogen dioxide; O_3_, ozone; PM_10_, particulate matter with aerodynamic diameter ≤10µm; AQI, air quality index.


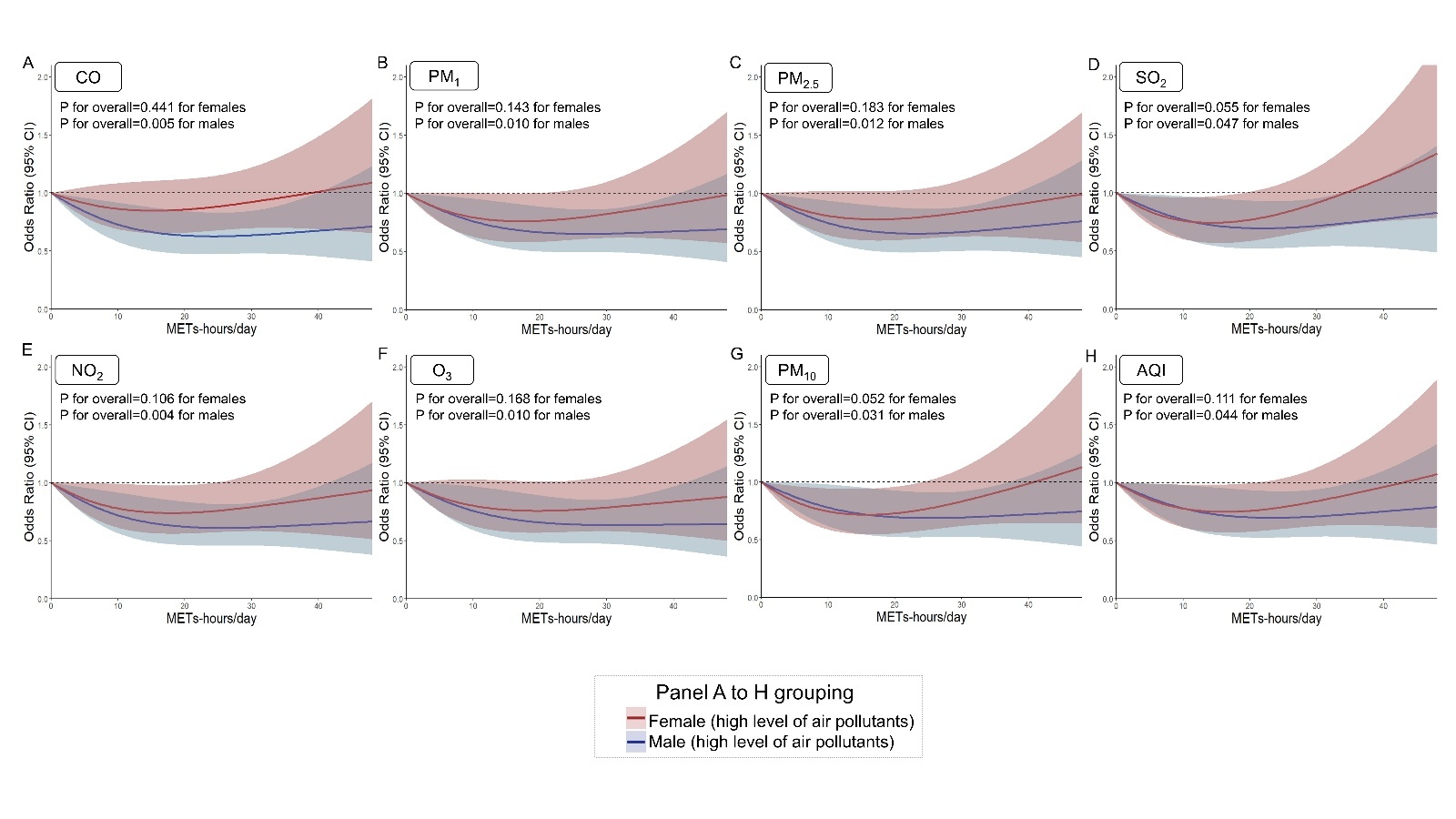


Supplementary figure 4. Dose–response curves of the associations of the METs of MVPA with the prevalence of CVD stratified by air pollutants concentration, CHARLS 2018. The cut off point of air pollutants is cohort-specific cut-off points at 75%. The panel depicts adjusted restricted cubic splines with 95% confidence. All models were adjusted for age, education, residence, marital status, household expenses per capita, smoke, drink, solid fuel usage, sleep duration, depressive symptoms, and non-communicable diseases. CVD, cardiovascular diseases; MVPA, moderate-vigorous physical activity; METs, Metabolic equivalents; CO, carbon monoxide; PM_1_, particulate matter with aerodynamic diameter ≤1µm; PM_2.5_, particulate matter with aerodynamic diameter ≤2.5µm; SO_2_, sulfur dioxide; NO_2_, nitrogen dioxide; O_3_, ozone; PM_10_, particulate matter with aerodynamic diameter ≤10µm; AQI, air quality index.


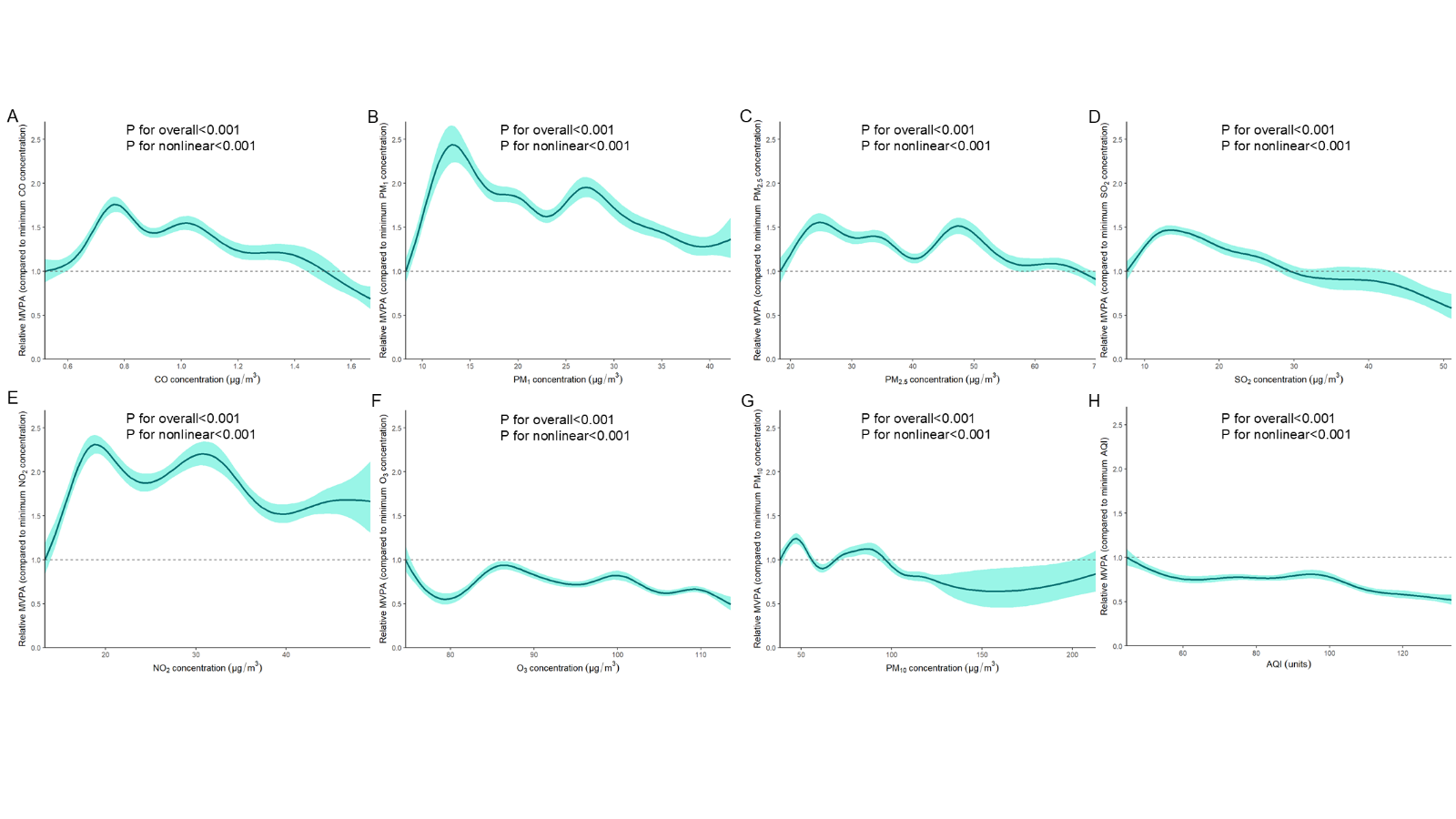


Supplementary figure 5. Independent association between air pollutants and MVPA, CHARLS 2018. All model adjusted for sex, age, education, residence, marital status, household expenses per capita, smoke, drink, solid fuel usage, sleep duration, depressive symptoms, and non-communicable diseases. MVPA, moderate-vigorous physical activity; CO, Carbon monoxide; PM_1_, particulate matter with aerodynamic diameter ≤1µm; PM_2.5_, particulate matter with aerodynamic diameter ≤2.5µm; SO_2_, Sulfur dioxide; NO_2_, nitrogen dioxide; O_3_, Ozone; PM_10_, particulate matter with aerodynamic diameter ≤10µm; AQI, air quality index.


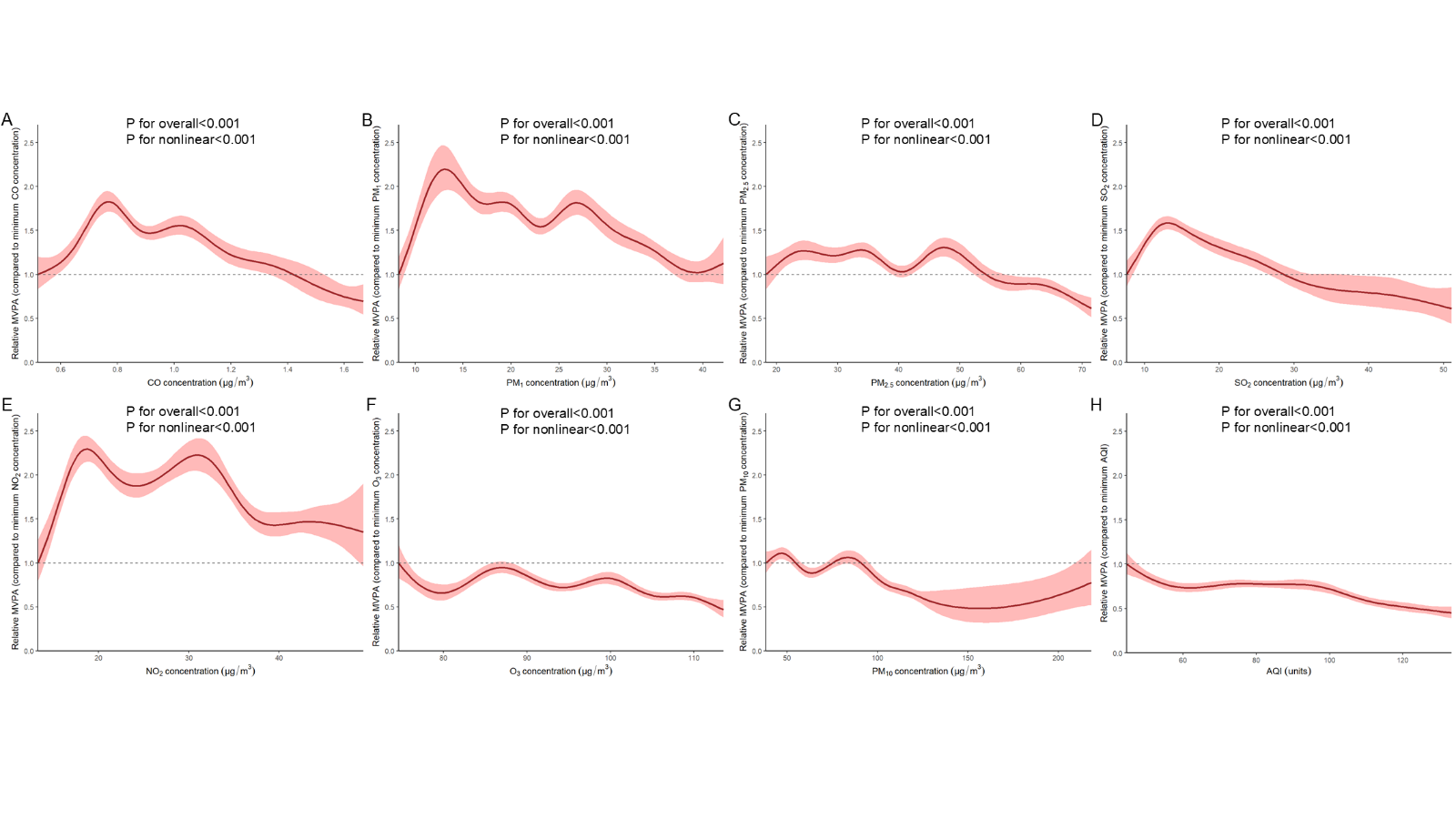


Supplementary figure 6. Independent association between air pollutants and MVPA in females, CHARLS 2018. All model adjusted for age, education, residence, marital status, household expenses per capita, smoke, drink, solid fuel usage, sleep duration, depressive symptoms, and non-communicable diseases. MVPA, moderate-vigorous physical activity; CO, Carbon monoxide; PM_1_, particulate matter with aerodynamic diameter ≤1µm; PM_2.5_, particulate matter with aerodynamic diameter ≤2.5µm; SO_2_, Sulfur dioxide; NO_2_, nitrogen dioxide; O_3_, Ozone; PM_10_, particulate matter with aerodynamic diameter ≤10µm; AQI, air quality index.


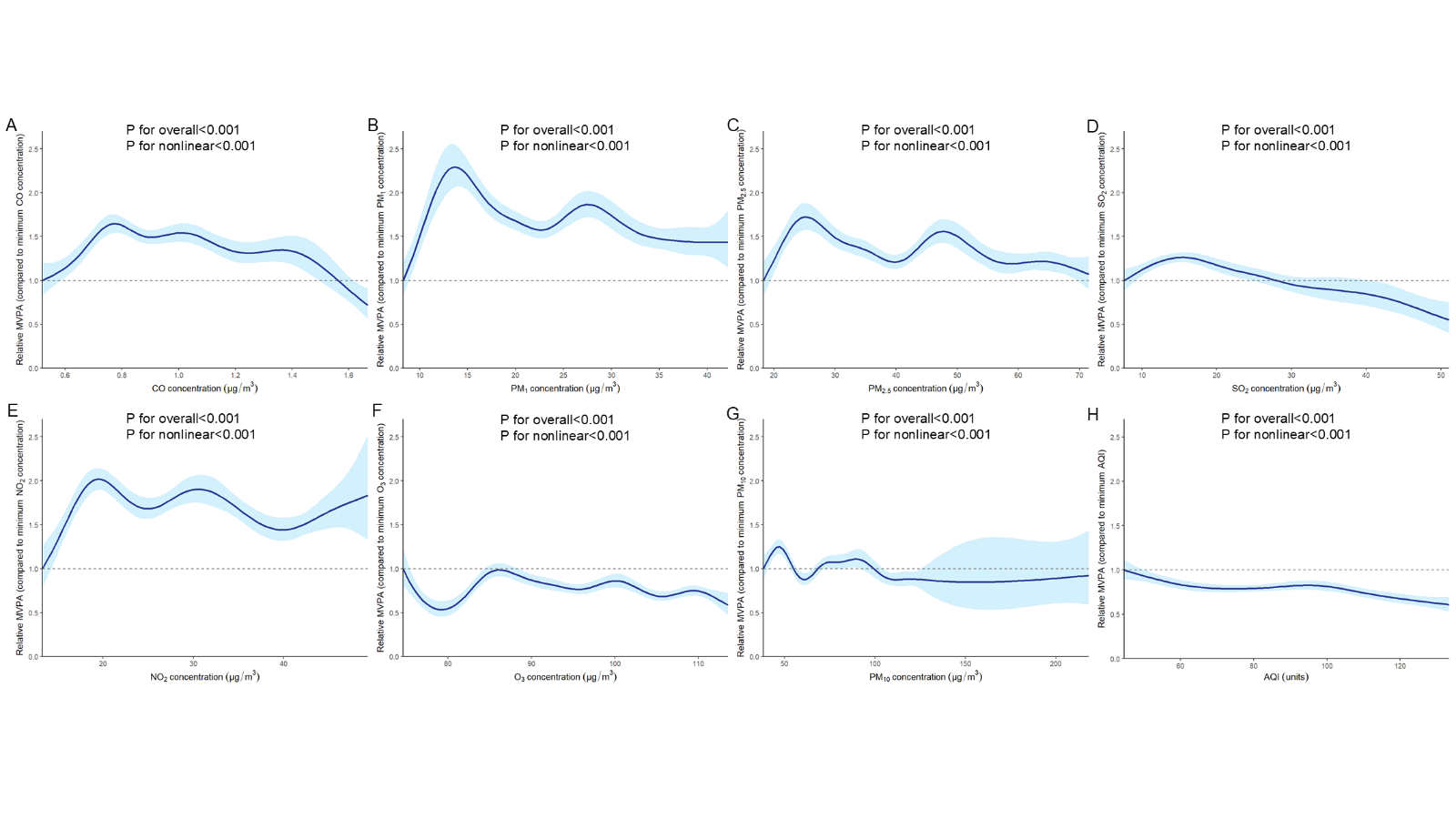


Supplementary figure 7. Independent association between air pollutants and MVPA in males, CHARLS 2018. All model adjusted for age, education, residence, marital status, household expenses per capita, smoke, drink, solid fuel usage, sleep duration, depressive symptoms, and non-communicable diseases. MVPA, moderate-vigorous physical activity; CO, Carbon monoxide; PM_1_, particulate matter with aerodynamic diameter ≤1µm; PM_2.5_, particulate matter with aerodynamic diameter ≤2.5µm; SO_2_, Sulfur dioxide; NO_2_, nitrogen dioxide; O_3_, Ozone; PM_10_, particulate matter with aerodynamic diameter ≤10µm; AQI, air quality index.

| Supplemental table 1. Independent association between MVPA and the prevalence of CVD, CHARLS 2018. |
| --- |
| \|  \|  \| Odd Ratio (95%CI) \| \| \| \| --- \| --- \| --- \| --- \| --- \| \|  \| Events/N \| Model 1^a^ \| Model 2^b^ \| Model 3^c^ \| \| Total \| 4123/17,138 \|  \|  \|  \| \| MVPA (h/day) \|  \|  \|  \|  \| \| 0 \| 1,947/6,426 \| Reference \| Reference \| Reference \| \| 0 -< 1 \| 556/2,313 \| 0.78 (0.70, 0.87) \| 0.79 (0.71, 0.89) \| 0.79 (0.70, 0.89) \| \| 1 -< 2 \| 593/2,479 \| 0.79 (0.70, 0.88) \| 0.79 (0.71, 0.88) \| 0.79 (0.71, 0.88) \| \| 2 -< 3 \| 366/1,771 \| 0.70 (0.61, 0.79) \| 0.70 (0.61, 0.79) \| 0.69 (0.61, 0.79) \| \| >= 3 \| 661/4,149 \| 0.58 (0.52, 0.64) \| 0.56 (0.51, 0.62) \| 0.55 (0.50, 0.61) \| \| ^#^P for trend \|  \| P < 0.001 \| P < 0.001 \| P < 0.001 \| \| Per 0.5h increase \|  \| 0.95 (0.94, 0.96) \| 0.95 (0.94, 0.96) \| 0.95 (0.94, 0.96) \| \| WHO recommendation^&^ \|  \|  \|  \|  \| \| Inactive \| 1,947/6,426 \| Reference \| Reference \| Reference \| \| Insufficiently active \| 438/1,749 \| 0.79 (0.70, 0.90) \| 0.81 (0.71, 0.92) \| 0.81 (0.71, 0.92) \| \| Physically active \| 1,738/8,963 \| 0.67 (0.62, 0.73) \| 0.67 (0.62, 0.72) \| 0.66 (0.61, 0.72) \|   ^a^ Model.1 adjusted for sex, age, education, residence, marital status, and household expenses per capita. ^b^ Model. 2: model. 1 further adjusted for smoke, drink, solid fuel usage, and sleep duration. ^c^ Model. 3: model. 2 further adjusted for depressive symptoms and non-communicable diseases. ^d^ P for trend was calculated using the duration spent on each category of MVPA as a continuous variable. ^e^ Based on the WHO Guidelines on Physical Activity and Sedentary Behaviour 2020, inactive was considered as not participating MVPA. And we use the same METs as 300 minutes of moderate physical activity or 150 minutes of vigorous physical activity as the dividing line between insufficiently active and physically active. CVD, cardiovascular disease; MVPA, moderate-vigorous physical activity; CI, confidence interval; WHO, World Health Organization; METs, metabolic equivalents. |

| \| Supplemental table 2. Independent association between MVPA and the prevalence of CVD in females, CHARLS 2018. \| \| \| \| \| \| --- \| --- \| --- \| --- \| --- \| \|  \|  \| Odd Ratio (95%CI) \| \| \| \|  \| Events/N \| Model 1^a^ \| Model 2^b^ \| Model 3^c^ \| \| Female \| 2,376/8,945 \|  \|  \|  \| \| MVPA (h/day) \|  \|  \|  \|  \| \| 0 \| 1,087/3,284 \| Reference \| Reference \| Reference \| \| 0 -< 1 \| 340/1,298 \| 0.80 (0.69, 0.92) \| 0.81 (0.70, 0.94) \| 0.81 (0.70, 0.95) \| \| 1 -< 2 \| 366/1,434 \| 0.78 (0.67, 0.90) \| 0.79 (0.68, 0.91) \| 0.79 (0.68, 0.92) \| \| 2 -< 3 \| 226/992 \| 0.71 (0.60, 0.84) \| 0.72 (0.61, 0.85) \| 0.72 (0.61, 0.86) \| \| >= 3 \| 357/1,937 \| 0.60 (0.52, 0.69) \| 0.57 (0.49, 0.66) \| 0.56 (0.48, 0.65) \| \| ^#^P for trend \|  \| P < 0.001 \| P < 0.001 \| P < 0.001 \| \| Per 0.5h increase \|  \| 0.95 (0.94, 0.97) \| 0.95 (0.94, 0.96) \| 0.95 (0.94, 0.96) \| \| WHO recommendation^&^ \|  \|  \|  \|  \| \| Inactive \| 1,087/3,284 \| Reference \| Reference \| Reference \| \| Insufficiently active \| 276/1,035 \| 0.80 (0.68, 0.93) \| 0.82 (0.70, 0.96) \| 0.81 (0.69, 0.96) \| \| Physically active \| 1,013/4,626 \| 0.69 (0.62, 0.77) \| 0.68 (0.62, 0.76) \| 0.68 (0.61, 0.76) \| |
| --- | --- | --- | --- | --- | --- | --- | --- | --- | --- | --- | --- | --- | --- | --- | --- | --- | --- | --- | --- | --- | --- | --- | --- | --- | --- | --- | --- | --- | --- | --- | --- | --- | --- | --- | --- | --- | --- | --- | --- | --- | --- | --- | --- | --- | --- | --- | --- | --- | --- | --- | --- | --- | --- | --- | --- | --- | --- | --- | --- | --- | --- | --- | --- | --- | --- | --- | --- | --- | --- | --- | --- | --- | --- | --- | --- | --- | --- | --- | --- | --- |
| ^a^ Model.1 adjusted for age, education, residence, marital status, and household expenses per capita. ^b^ Model. 2: model. 1 further adjusted for smoke, drink, solid fuel usage, and sleep duration. ^c^ Model. 3: model. 2 further adjusted for depressive symptoms and non-communicable diseases. ^d^ P for trend was calculated using the duration spent on each category of MVPA as a continuous variable. ^e^ Based on the WHO Guidelines on Physical Activity and Sedentary Behaviour 2020, inactive was considered as not participating MVPA. And we use the same METs as 300 minutes of moderate physical activity or 150 minutes of vigorous physical activity as the dividing line between insufficiently active and physically active. CVD, cardiovascular disease; MVPA, moderate-vigorous physical activity; CI, confidence interval; WHO, World Health Organization; METs, metabolic equivalents. |
|  |

| Supplemental table 3. Independent association between MVPA and the prevalence of CVD in males, CHARLS 2018. |  |
| --- | --- |
| \|  \|  \| Odd Ratio (95%CI) \| \| \| \| --- \| --- \| --- \| --- \| --- \| \|  \| Events/N \| Model 1^a^ \| Model 2^b^ \| Model 3^c^ \| \| Male \| 1,747/8,193 \|  \|  \|  \| \| MVPA (h/day) \|  \|  \|  \|  \| \| 0 \| 860/3,142 \| Reference \| Reference \| Reference \| \| 0 -< 1 \| 216/1,015 \| 0.76 (0.64, 0.91) \| 0.77 (0.65, 0.92) \| 0.76 (0.64, 0.91) \| \| 1 -< 2 \| 227/1,045 \| 0.81 (0.68, 0.96) \| 0.80 (0.68, 0.95) \| 0.79 (0.66, 0.94) \| \| 2 -< 3 \| 140/779 \| 0.68 (0.55, 0.83) \| 0.68 (0.55, 0.83) \| 0.66 (0.54, 0.81) \| \| >= 3 \| 304/2,212 \| 0.57 (0.49, 0.66) \| 0.56 (0.48, 0.65) \| 0.54 (0.47, 0.64) \| \| ^#^P for trend \|  \| P < 0.001 \| P < 0.001 \| P < 0.001 \| \| Per 0.5h increase \|  \| 0.95 (0.94, 0.97) \| 0.95 (0.94, 0.96) \| 0.95 (0.93, 0.96) \| \| WHO recommendation^&^ \|  \|  \|  \|  \| \| Inactive \| 860/3,142 \| Reference \| Reference \| Reference \| \| Insufficiently active \| 162/714 \| 0.80 (0.65, 0.97) \| 0.80 (0.66, 0.98) \| 0.80 (0.66, 0.99) \| \| Physically active \| 725/4,337 \| 0.66 (0.59, 0.74) \| 0.65 (0.58, 0.73) \| 0.64 (0.57, 0.72) \|   ^a^ Model.1 adjusted for age, education, residence, marital status, and household expenses per capita. ^b^ Model. 2: model. 1 further adjusted for smoke, drink, solid fuel usage, and sleep duration. ^c^ Model. 3: model. 2 further adjusted for depressive symptoms and non-communicable diseases. ^d^ P for trend was calculated using the duration spent on each category of MVPA as a continuous variable. ^e^ Based on the WHO Guidelines on Physical Activity and Sedentary Behaviour 2020, inactive was considered as not participating MVPA. And we use the same METs as 300 minutes of moderate physical activity or 150 minutes of vigorous physical activity as the dividing line between insufficiently active and physically active. CVD, cardiovascular disease; MVPA, moderate-vigorous physical activity; CI, confidence interval; WHO, World Health Organization; METs, metabolic equivalents. | |

| Supplemental table 4. Independent association between air pollutants and the prevalence of CVD, CHARLS 2018. | | | | | | | | | |
| --- | --- | --- | --- | --- | --- | --- | --- | --- | --- |
|  |  | Odd Ratio (95%CI) | | |  |  | Odd Ratio (95%CI) | | |
|  | Events/N | Model 1^a^ | Model 2^b^ | Model 3^c^ |  | Events/N | Model 1^a^ | Model 2^b^ | Model 3^c^ |
| Total | 4123/17,138 |  |  |  |  |  |  |  |  |
| CO (µg/m^3^) |  |  |  |  | NO_2_ (µg/m^3^) |  |  |  |  |
| Q1 (0.52-0.81) | 1,028/4,285 | Reference | Reference | Reference | Q1 (13.28-20.56) | 1,071/4,285 | Reference | Reference | Reference |
| Q2 (0.81-0.92) | 881/4,285 | 0.79 (0.71, 0.88) | 0.79 (0.71, 0.87) | 0.77 (0.69, 0.86) | Q2 (20.56-26.84) | 938/4,285 | 0.80 (0.72, 0.89) | 0.82 (0.74, 0.91) | 0.83 (0.75, 0.93) |
| Q3 (0.92-1.08) | 941/4,284 | 0.88 (0.79, 0.98) | 0.87 (0.79, 0.97) | 0.86 (0.77, 0.96) | Q3 (26.84-35.29) | 927/4,284 | 0.81 (0.73, 0.90) | 0.84 (0.76, 0.93) | 0.85 (0.76, 0.94) |
| Q4 (1.08-1.67) | 1,273/4,284 | 1.41 (1.28, 1.56) | 1.44 (1.30, 1.59) | 1.45 (1.31, 1.61) | Q4 (35.29-49.32) | 1,187/4,284 | 1.13 (1.02, 1.25) | 1.19 (1.08, 1.32) | 1.28 (1.15, 1.42) |
| ^#^P for trend |  | P < 0.001 | P < 0.001 | P < 0.001 | ^#^P for trend |  | P < 0.05 | P < 0.001 | P < 0.001 |
| Per 0.1 µg/m^3^ increase |  | 1.04 (1.02, 1.05) | 1.04 (1.03, 1.06) | 1.05 (1.03, 1.06) | Per 5 µg/m^3^ increase |  | 1.02 (1.00, 1.04) | 1.04 (1.01, 1.06) | 1.05 (1.03, 1.07) |
| PM_1_ (µg/m^3^) |  |  |  |  | O_3_ (µg/m^3^) |  |  |  |  |
| Q1 (8.28-18.78) | 973/4,285 | Reference | Reference | Reference | Q1 (74.61-88.32) | 970/4,285 | Reference | Reference | Reference |
| Q2 (18.78-23.26) | 931/4,285 | 0.87 (0.78, 0.97) | 0.91 (0.81, 1.01) | 0.92 (0.82, 1.02) | Q2 (88.32-95.86) | 1,017/4,285 | 1.04 (0.94, 1.15) | 1.07 (0.96, 1.18) | 1.10 (0.99, 1.22) |
| Q3 (23.26-29.92) | 958/4,284 | 0.89 (0.80, 0.98) | 0.93 (0.83, 1.03) | 0.95 (0.86, 1.06) | Q3 (95.86-105.92) | 1,041/4,284 | 1.16 (1.05, 1.28) | 1.20 (1.08, 1.33) | 1.24 (1.11, 1.38) |
| Q4 (29.92-42.16) | 1,261/4,284 | 1.42 (1.29, 1.58) | 1.51 (1.37, 1.67) | 1.61 (1.45, 1.78) | Q4 (105.92-113.55) | 1,095/4,284 | 1.19 (1.08, 1.32) | 1.26 (1.14, 1.40) | 1.36 (1.22, 1.51) |
| ^#^P for trend |  | P < 0.001 | P < 0.001 | P < 0.001 | ^#^P for trend |  | P < 0.001 | P < 0.001 | P < 0.001 |
| Per 5 µg/m^3^ increase |  | 1.04 (1.02, 1.06) | 1.06 (1.03, 1.08) | 1.07 (1.05, 1.10) | Per 10 µg/m^3^ increase |  | 1.06 (1.02, 1.10) | 1.08 (1.04, 1.12) | 1.11 (1.07, 1.15) |
| PM_2.5_ (µg/m^3^) |  |  |  |  | PM_10_ (µg/m^3^) |  |  |  |  |
| Q1 (18.25-33.31) | 900/4,285 | Reference | Reference | Reference | Q1 (38.38-53.60) | 795/4,285 | Reference | Reference | Reference |
| Q2 (33.31-40.94) | 1,118/4,285 | 1.22 (1.10, 1.35) | 1.23 (1.10, 1.36) | 1.24 (1.11, 1.37) | Q2 (53.60-71.25) | 1,051/4,285 | 1.32 (1.18, 1.47) | 1.31 (1.18, 1.46) | 1.30 (1.17, 1.45) |
| Q3 (40.94-54.42) | 829/4,284 | 0.82 (0.73, 0.91) | 0.84 (0.75, 0.94) | 0.83 (0.74, 0.92) | Q3 (71.25-100.88) | 1,000/4,284 | 1.31 (1.18, 1.46) | 1.31 (1.17, 1.46) | 1.29 (1.16, 1.44) |
| Q4 (54.42-71.51) | 1,276/4,284 | 1.58 (1.43, 1.75) | 1.65 (1.49, 1.83) | 1.73 (1.55, 1.92) | Q4 (100.88-218.17) | 1,277/4,284 | 1.89 (1.70, 2.10) | 1.92 (1.73, 2.13) | 2.01 (1.81, 2.24) |
| ^#^P for trend |  | P < 0.001 | P < 0.001 | P < 0.001 | ^#^P for trend |  | P < 0.001 | P < 0.001 | P < 0.001 |
| Per 5 µg/m^3^ increase |  | 1.04 (1.03, 1.06) | 1.05 (1.04, 1.06) | 1.05 (1.04, 1.07) | Per 10 µg/m^3^ increase |  | 1.07 (1.06, 1.09) | 1.08 (1.06, 1.09) | 1.08 (1.07, 1.10) |
| SO_2_ (µg/m^3^) |  |  |  |  | AQI^^^ (units) |  |  |  |  |
| Q1 (7.59-12.63) | 989/4,285 | Reference | Reference | Reference | Q1 (44.58-68.14) | 956/4,285 | Reference | Reference | Reference |
| Q2 (12.63-15.48) | 773/4,285 | 0.71 (0.63, 0.79) | 0.73 (0.65, 0.81) | 0.71 (0.63, 0.79) | Q2 (68.14-81.65) | 858/4,285 | 0.84 (0.76, 0.94) | 0.85 (0.76, 0.94) | 0.82 (0.73, 0.91) |
| Q3 (15.48-21.29) | 1,141/4,284 | 1.29 (1.16, 1.42) | 1.27 (1.14, 1.40) | 1.26 (1.14, 1.40) | Q3 (81.65-104.02) | 1,048/4,284 | 1.07 (0.96, 1.18) | 1.07 (0.97, 1.19) | 1.05 (0.95, 1.17) |
| Q4 (21.29-51.07) | 1,220/4,284 | 1.43 (1.29, 1.58) | 1.45 (1.31, 1.60) | 1.54 (1.39, 1.71) | Q4 (104.02-133.31) | 1,261/4,284 | 1.48 (1.34, 1.64) | 1.51 (1.36, 1.67) | 1.55 (1.40, 1.72) |
| ^#^P for trend |  | P < 0.001 | P < 0.001 | P < 0.001 | ^#^P for trend |  | P < 0.001 | P < 0.001 | P < 0.001 |
| Per 5 µg/m^3^ increase |  | 1.10 (1.07, 1.12) | 1.10 (1.07, 1.12) | 1.11 (1.09, 1.14) | Per 10 units increase |  | 1.08 (1.06, 1.09) | 1.08 (1.07, 1.10) | 1.09 (1.07, 1.11) |
| ^a^ Model.1 adjusted for sex, age, education, residence, marital status, and household expenses per capita. ^b^ Model. 2: model.1 further adjusted for smoke, drink, solid fuel usage, and sleep duration. ^c^ Model. 3: model. 2 further adjusted for depressive symptoms, and non-communicable diseases. ^d^ P for trend was calculated using the duration spent on each category of MVPA as a continuous variable. ^e^ The AQI is a numerical indicator used to assess air quality, based on the concentrations of multiple pollutants, including CO, PM_2.5_, SO_2_, NO_2_, O_3_, and PM_10_. CVD, cardiovascular diseases; CI, confidence interval; CO, Carbon monoxide; PM_1_, particulate matter with aerodynamic diameter ≤1µm; PM_2.5_, particulate matter with aerodynamic diameter ≤2.5µm; SO_2_, Sulfur dioxide; NO_2_, nitrogen dioxide; O_3_, Ozone; PM_10_, particulate matter with aerodynamic diameter ≤10µm; AQI, air quality index. | | | | | | | | | |

| Supplemental table 5. Independent association between air pollutants and the prevalence of CVD in females, CHARLS 2018. | | | | | | | | | |  |
| --- | --- | --- | --- | --- | --- | --- | --- | --- | --- | --- |
|  |  | Odd Ratio (95%CI) | | |  |  | Odd Ratio (95%CI) | | | |
|  | Events/N | Model 1^a^ | Model 2^b^ | Model 3^c^ |  | Events/N | Model 1^a^ | Model 2^b^ | Model 3^c^ | |
| Female | 2,376/8,945 |  |  |  |  |  |  |  |  | |
| CO (µg/m^3^) |  |  |  |  | NO_2_ (µg/m^3^) |  |  |  |  | |
| Q1 (0.52-0.81) | 619/2,237 | Reference | Reference | Reference | Q1 (13.28-20.56) | 637/2,237 | Reference | Reference | Reference | |
| Q2 (0.81-0.92) | 505/2,236 | 0.74 (0.64, 0.85) | 0.73 (0.63, 0.84) | 0.73 (0.63, 0.84) | Q2 (20.56-26.84) | 532/2,236 | 0.75 (0.65, 0.85) | 0.77 (0.67, 0.88) | 0.79 (0.68, 0.91) | |
| Q3 (0.92-1.08) | 537/2,236 | 0.80 (0.70, 0.92) | 0.79 (0.69, 0.91) | 0.79 (0.68, 0.91) | Q3 (26.84-35.29) | 535/2,236 | 0.78 (0.68, 0.89) | 0.82 (0.72, 0.94) | 0.83 (0.72, 0.96) | |
| Q4 (1.08-1.67) | 715/2,236 | 1.26 (1.11, 1.44) | 1.30 (1.13, 1.48) | 1.32 (1.15, 1.51) | Q4 (35.29-49.32) | 672/2,236 | 1.04 (0.91, 1.19) | 1.12 (0.98, 1.28) | 1.19 (1.03, 1.37) | |
| ^#^P for trend |  | P < 0.001 | P < 0.001 | P < 0.001 | ^#^P for trend |  | 0.436 | 0.064 | P < 0.05 | |
| Per 0.1 µg/m^3^ increase |  | 1.01 (0.99, 1.03) | 1.02 (1.00, 1.04) | 1.02 (1.00, 1.05) | Per 5 µg/m^3^ increase |  | 1.00 (0.97, 1.03) | 1.02 (0.99, 1.05) | 1.03 (1.00, 1.06) | |
| PM_1_ (µg/m^3^) |  |  |  |  | O_3_ (µg/m^3^) |  |  |  |  | |
| Q1 (8.28-18.78) | 572/2,237 | Reference | Reference | Reference | Q1 (74.61-88.32) | 582/2,237 | Reference | Reference | Reference | |
| Q2 (18.78-23.25) | 551/2,236 | 0.89 (0.77, 1.02) | 0.93 (0.81, 1.07) | 0.95 (0.82, 1.10) | Q2 (88.32-95.86) | 573/2,236 | 0.97 (0.85, 1.12) | 1.01 (0.88, 1.16) | 1.04 (0.91, 1.20) | |
| Q3 (23.25-29.92) | 551/2,236 | 0.86 (0.75, 0.99) | 0.90 (0.78, 1.04) | 0.93 (0.81, 1.08) | Q3 (95.86-105.92) | 605/2,236 | 1.11 (0.96, 1.27) | 1.18 (1.03, 1.35) | 1.23 (1.07, 1.41) | |
| Q4 (29.92-42.16) | 702/2,236 | 1.31 (1.15, 1.50) | 1.40 (1.22, 1.61) | 1.47 (1.28, 1.69) | Q4 (105.92-113.55) | 616/2,236 | 1.10 (0.96, 1.26) | 1.18 (1.03, 1.36) | 1.27 (1.10, 1.46) | |
| ^#^P for trend |  | P < 0.001 | P < 0.001 | P < 0.001 | ^#^P for trend |  | 0.064 | P < 0.01 | P < 0.001 | |
| Per 5 µg/m^3^ increase |  | 1.01 (0.98, 1.04) | 1.03 (1.00, 1.07) | 1.05 (1.01, 1.08) | Per 10 µg/m^3^ increase |  | 1.02 (0.98, 1.07) | 1.05 (1.00, 1.10) | 1.08 (1.02, 1.13) | |
| PM_2.5_ (µg/m^3^) |  |  |  |  | PM_10_ (µg/m^3^) |  |  |  |  | |
| Q1 (18.25-33.31) | 519/2,237 | Reference | Reference | Reference | Q1 (38.38-53.60) | 455/2,237 | Reference | Reference | Reference | |
| Q2 (33.31-40.94) | 667/2,236 | 1.30 (1.14, 1.49) | 1.30 (1.14, 1.50) | 1.33 (1.15, 1.53) | Q2 (53.60-71.25) | 598/2,236 | 1.33 (1.15, 1.53) | 1.33 (1.15, 1.54) | 1.35 (1.17, 1.56) | |
| Q3 (40.94-54.42) | 479/2,236 | 0.81 (0.70, 0.94) | 0.85 (0.73, 0.98) | 0.84 (0.72, 0.98) | Q3 (71.25-100.88) | 613/2,236 | 1.46 (1.27, 1.68) | 1.46 (1.26, 1.69) | 1.48 (1.28, 1.71) | |
| Q4 (52.42-71.51) | 711/2,236 | 1.51 (1.32, 1.73) | 1.59 (1.38, 1.83) | 1.65 (1.43, 1.90) | Q4 (100.88-218.17) | 710/2,236 | 1.82 (1.58, 2.09) | 1.86 (1.62, 2.15) | 1.94 (1.68, 2.25) | |
| ^#^P for trend |  | P < 0.001 | P < 0.001 | P < 0.001 | ^#^P for trend |  | P < 0.001 | P < 0.001 | P < 0.001 | |
| Per 5 µg/m^3^ increase |  | 1.03 (1.01, 1.05) | 1.04 (1.02, 1.06) | 1.04 (1.03, 1.06) | Per 10 µg/m^3^ increase |  | 1.07 (1.05, 1.08) | 1.07 (1.05, 1.09) | 1.08 (1.06, 1.10) | |
| SO_2_ (µg/m^3^) |  |  |  |  | AQI^^^ (units) |  |  |  |  | |
| Q1 (7.59-12.63) | 582/2,237 | Reference | Reference | Reference | Q1 (44.58-68.14) | 569/2,237 | Reference | Reference | Reference | |
| Q2 (12.63-15.46) | 430/2,236 | 0.64 (0.56, 0.74) | 0.67 (0.58, 0.78) | 0.66 (0.57, 0.76) | Q2 (68.14-81.65) | 476/2,236 | 0.77 (0.67, 0.89) | 0.79 (0.68, 0.91) | 0.78 (0.67, 0.90) | |
| Q3 (15.46-21.29) | 672/2,236 | 1.31 (1.14, 1.49) | 1.28 (1.11, 1.46) | 1.27 (1.11, 1.46) | Q3 (81.65-102.12) | 633/2,236 | 1.11 (0.97, 1.27) | 1.12 (0.98, 1.29) | 1.13 (0.98, 1.30) | |
| Q4 (21.29-51.07) | 692/2,236 | 1.33 (1.16, 1.52) | 1.34 (1.17, 1.53) | 1.42 (1.23, 1.63) | Q4 (102.12-133.31) | 698/2,236 | 1.34 (1.17, 1.53) | 1.37 (1.20, 1.57) | 1.41 (1.23, 1.62) | |
| ^#^P for trend |  | P < 0.001 | P < 0.001 | P < 0.001 | ^#^P for trend |  | P < 0.001 | P < 0.001 | P < 0.001 | |
| Per 5 µg/m^3^ increase |  | 1.07 (1.04, 1.10) | 1.06 (1.03, 1.10) | 1.08 (1.05, 1.12) | Per 10 µg/m^3^ increase |  | 1.06 (1.04, 1.08) | 1.07 (1.05, 1.09) | 1.07 (1.05, 1.10) | |
| ^a^ Model.1 adjusted for age, education, residence, marital status, and household expenses per capita. ^b^ Model. 2: model.1 further adjusted for smoke, drink, solid fuel usage, and sleep duration. ^c^ Model. 3: model. 2 further adjusted for depressive symptoms, and non-communicable diseases. ^d^ P for trend was calculated using the duration spent on each category of MVPA as a continuous variable. ^e^ The AQI is a numerical indicator used to assess air quality, based on the concentrations of multiple pollutants, including CO, PM_2.5_, SO_2_, NO_2_, O_3_, and PM_10_. CVD, cardiovascular diseases; CI, confidence interval; CO, Carbon monoxide; PM_1_, particulate matter with aerodynamic diameter ≤1µm; PM_2.5_, particulate matter with aerodynamic diameter ≤2.5µm; SO_2_, Sulfur dioxide; NO_2_, nitrogen dioxide; O_3_, Ozone; PM_10_, particulate matter with aerodynamic diameter ≤10µm; AQI, air quality index. | | | | | | | | | | |

| Supplemental table 6. Independent association between air pollutants and the prevalence of CVD in males, CHARLS 2018. | | | | | | | | | |  |
| --- | --- | --- | --- | --- | --- | --- | --- | --- | --- | --- |
|  |  | Odd Ratio (95%CI) | | |  |  | Odd Ratio (95%CI) | | | |
|  | Events/N | Model 1^a^ | Model 2^b^ | Model 3^c^ |  | Events/N | Model 1^a^ | Model 2^b^ | Model 3^c^ | |
| Male | 1,747/8,193 |  |  |  |  |  |  |  |  | |
| CO (µg/m^3^) |  |  |  |  | NO_2_ (µg/m^3^) |  |  |  |  | |
| Q1 (0.52-0.81) | 409/2,049 | Reference | Reference | Reference | Q1 (13.28-20.92) | 434/2,049 | Reference | Reference | Reference | |
| Q2 (0.81-0.92) | 375/2,048 | 0.87 (0.74, 1.02) | 0.87 (0.74, 1.02) | 0.83 (0.70, 0.97) | Q2 (20.92-26.84) | 406/2,048 | 0.88 (0.76, 1.03) | 0.90 (0.77, 1.05) | 0.91 (0.78, 1.07) | |
| Q3 (0.92-1.08) | 403/2,048 | 0.98 (0.84, 1.15) | 0.98 (0.84, 1.15) | 0.96 (0.82, 1.13) | Q3 (26.84-35.29) | 394/2,048 | 0.86 (0.74, 1.01) | 0.89 (0.76, 1.04) | 0.90 (0.77, 1.06) | |
| Q4 (1.08-1.67) | 560/2,048 | 1.64 (1.41, 1.90) | 1.66 (1.43, 1.93) | 1.65 (1.41, 1.93) | Q4 (35.29-49.32) | 513/2,048 | 1.24 (1.07, 1.45) | 1.30 (1.11, 1.51) | 1.42 (1.21, 1.65) | |
| ^#^P for trend |  | P < 0.001 | P < 0.001 | P < 0.001 | ^#^P for trend |  | P < 0.01 | P < 0.01 | P < 0.001 | |
| Per 0.1 µg/m^3^ increase |  | 1.07 (1.04, 1.09) | 1.07 (1.05, 1.10) | 1.08 (1.05, 1.10) | Per 5 µg/m^3^ increase |  | 1.05 (1.01, 1.08) | 1.06 (1.03, 1.09) | 1.08 (1.04, 1.11) | |
| PM_1_ (µg/m^3^) |  |  |  |  | O_3_ (µg/m^3^) |  |  |  |  | |
| Q1 (8.28-18.78) | 401/2,049 | Reference | Reference | Reference | Q1 (74.61-88.32) | 388/2,049 | Reference | Reference | Reference | |
| Q2 (18.78-23.26) | 382/2,048 | 0.86 (0.73, 1.01) | 0.89 (0.76, 1.05) | 0.89 (0.76, 1.05) | Q2 (88.32-95.86) | 439/2,048 | 1.14 (0.97, 1.33) | 1.16 (0.99, 1.36) | 1.19 (1.01, 1.40) | |
| Q3 (23.26-29.92) | 403/2,048 | 0.91 (0.77, 1.06) | 0.95 (0.81, 1.11) | 0.97 (0.82, 1.14) | Q3 (95.86-105.92) | 440/2,048 | 1.23 (1.05, 1.44) | 1.25 (1.07, 1.47) | 1.29 (1.10, 1.52) | |
| Q4 (29.92-42.16) | 561/2,048 | 1.58 (1.36, 1.84) | 1.66 (1.43, 1.94) | 1.81 (1.55, 2.13) | Q4 (105.92-113.55) | 480/2,048 | 1.32 (1.13, 1.54) | 1.38 (1.18, 1.61) | 1.51 (1.29, 1.77) | |
| ^#^P for trend |  | P < 0.001 | P < 0.001 | P < 0.001 | ^#^P for trend |  | P < 0.001 | P < 0.001 | P < 0.001 | |
| Per 5 µg/m^3^ increase |  | 1.08 (1.04, 1.11) | 1.09 (1.05, 1.13) | 1.11 (1.07, 1.15) | Per 10 µg/m^3^ increase |  | 1.10 (1.04, 1.16) | 1.12 (1.06, 1.18) | 1.16 (1.09, 1.22) | |
| PM_2.5_ (µg/m^3^) |  |  |  |  | PM_10_ (µg/m^3^) |  |  |  |  | |
| Q1 (18.25-33.31) | 381/2,049 | Reference | Reference | Reference | Q1 (38.38-53.60) | 341/2,049 | Reference | Reference | Reference | |
| Q2 (33.31-40.96) | 449/2,048 | 1.12 (0.96, 1.31) | 1.13 (0.97, 1.32) | 1.13 (0.97, 1.33) | Q2 (53.60-72.06) | 448/2,048 | 1.28 (1.09, 1.50) | 1.28 (1.09, 1.50) | 1.24 (1.05, 1.46) | |
| Q3 (40.96-55.13) | 351/2,048 | 0.82 (0.70, 0.96) | 0.84 (0.72, 0.99) | 0.81 (0.69, 0.96) | Q3 (72.06-100.88) | 391/2,048 | 1.15 (0.98, 1.36) | 1.15 (0.98, 1.36) | 1.12 (0.94, 1.32) | |
| Q4 (55.13-71.51) | 566/2,048 | 1.68 (1.45, 1.96) | 1.74 (1.50, 2.03) | 1.84 (1.57, 2.16) | Q4 (100.88-218.17) | 567/2,048 | 1.98 (1.69, 2.31) | 1.99 (1.70, 2.33) | 2.11 (1.80, 2.48) | |
| ^#^P for trend |  | P < 0.001 | P < 0.001 | P < 0.001 | ^#^P for trend |  | P < 0.001 | P < 0.001 | P < 0.001 | |
| Per 5 µg/m^3^ increase |  | 1.06 (1.04, 1.08) | 1.06 (1.04, 1.09) | 1.07 (1.05, 1.09) | Per 10 µg/m^3^ increase |  | 1.09 (1.07, 1.11) | 1.09 (1.07, 1.11) | 1.09 (1.07, 1.12) | |
| SO_2_ (µg/m^3^) |  |  |  |  | AQI^^^ (units) |  |  |  |  | |
| Q1 (7.59-12.63) | 406/2,049 | Reference | Reference | Reference | Q1 (44.58-68.14) | 385/2,049 | Reference | Reference | Reference | |
| Q2 (12.63-15.48) | 344/2,048 | 0.79 (0.67, 0.93) | 0.81 (0.69, 0.96) | 0.79 (0.67, 0.93) | Q2 (68.14-81.65) | 375/2,048 | 0.93 (0.79, 1.09) | 0.93 (0.79, 1.10) | 0.87 (0.74, 1.03) | |
| Q3 (15.48-21.45) | 468/2,048 | 1.27 (1.09, 1.48) | 1.26 (1.08, 1.47) | 1.25 (1.07, 1.47) | Q3 (81.65-104.02) | 426/2,048 | 1.06 (0.90, 1.24) | 1.07 (0.91, 1.25) | 1.02 (0.86, 1.20) | |
| Q4 (21.45-51.07) | 529/2,048 | 1.59 (1.36, 1.85) | 1.60 (1.37, 1.87) | 1.73 (1.47, 2.02) | Q4 (104.02-133.31) | 561/2,048 | 1.69 (1.45, 1.97) | 1.71 (1.47, 2.00) | 1.78 (1.52, 2.08) | |
| ^#^P for trend |  | P < 0.001 | P < 0.001 | P < 0.001 | ^#^P for trend |  | P < 0.001 | P < 0.001 | P < 0.001 | |
| Per 5 µg/m^3^ increase |  | 1.13 (1.09, 1.17) | 1.13 (1.09, 1.17) | 1.15 (1.11, 1.19) | Per 10 units increase |  | 1.10 (1.07, 1.13) | 1.10 (1.08, 1.13) | 1.11 (1.08, 1.14) | |
| ^a^ Model.1 adjusted for age, education, residence, marital status, and household expenses per capita. ^b^ Model. 2: model.1 further adjusted for smoke, drink, solid fuel usage, and sleep duration. ^c^ Model. 3: model. 2 further adjusted for depressive symptoms, and non-communicable diseases. ^d^ P for trend was calculated using the duration spent on each category of MVPA as a continuous variable. ^e^ The AQI is a numerical indicator used to assess air quality, based on the concentrations of multiple pollutants, including CO, PM_2.5_, SO_2_, NO_2_, O_3_, and PM_10_. CVD, cardiovascular diseases; CI, confidence interval; CO, Carbon monoxide; PM_1_, particulate matter with aerodynamic diameter ≤1µm; PM_2.5_, particulate matter with aerodynamic diameter ≤2.5µm; SO_2_, Sulfur dioxide; NO_2_, nitrogen dioxide; O_3_, Ozone; PM_10_, particulate matter with aerodynamic diameter ≤10µm; AQI, air quality index. | | | | | | | | | | |

| Supplemental table 7. Joint association of MVPA and air pollutants with the prevalence of CVD, CHARLS 2018. |
| --- |
| \|  \| Odd Ratio (95%CI) \| \| \|  \| Odd Ratio (95%CI) \| \| \| \| --- \| --- \| --- \| --- \| --- \| --- \| --- \| --- \| \|  \| Physically active^&^ \| Insufficiently active^&^ \| Inactive^&^ \|  \| Physically active^&^ \| Insufficiently active^&^ \| Inactive^&^ \| \| CO (µg/m^3^) \|  \|  \|  \| NO_2_ (µg/m^3^) \|  \|  \|  \| \| Q1 \| Reference \| 1.10 (0.86, 1.40) \| 1.38 (1.18, 1.62) \| Q1 \| Reference \| 1.13 (0.87, 1.46) \| 1.45 (1.24, 1.70) \| \| Q2 \| 0.74 (0.64, 0.87) \| 0.85 (0.64, 1.13) \| 1.11 (0.94, 1.30) \| Q2 \| 0.78 (0.67, 0.91) \| 1.12 (0.88, 1.43) \| 1.19 (1.02, 1.40) \| \| Q3 \| 0.76 (0.65, 0.89) \| 1.19 (0.93, 1.52) \| 1.31 (1.11, 1.54) \| Q3 \| 0.80 (0.68, 0.92) \| 0.96 (0.73, 1.25) \| 1.32 (1.12, 1.54) \| \| Q4 \| 1.46 (1.25, 1.69) \| 1.48 (1.16, 1.88) \| 1.92 (1.66, 2.24) \| Q4 \| 1.29 (1.11, 1.50) \| 1.39 (1.10, 1.77) \| 1.72 (1.49, 2.00) \| \| PM_1_ (µg/m^3^) \|  \|  \|  \| O_3_ (µg/m^3^) \|  \|  \|  \| \| Q1 \| Reference \| 1.37 (1.05, 1.78) \| 1.48 (1.26, 1.74) \| Q1 \| Reference \| 1.49 (1.16, 1.91) \| 1.70 (1.45, 2.00) \| \| Q2 \| 0.94 (0.81, 1.10) \| 0.95 (0.74, 1.22) \| 1.40 (1.19, 1.65) \| Q2 \| 1.27 (1.09, 1.48) \| 1.35 (1.06, 1.74) \| 1.61 (1.37, 1.89) \| \| Q3 \| 0.92 (0.78, 1.07) \| 1.39 (1.09, 1.79) \| 1.41 (1.20, 1.66) \| Q3 \| 1.23 (1.05, 1.43) \| 1.57 (1.21, 2.03) \| 2.10 (1.79, 2.45) \| \| Q4 \| 1.67 (1.43, 1.95) \| 1.71 (1.33, 2.20) \| 2.24 (1.93, 2.60) \| Q4 \| 1.50 (1.28, 1.76) \| 1.60 (1.24, 2.07) \| 2.00 (1.72, 2.33) \| \| PM_2.5_ (µg/m^3^) \|  \|  \|  \| PM_10_ (µg/m^3^) \|  \|  \|  \| \| Q1 \| Reference \| 1.24 (0.95, 1.63) \| 1.38 (1.17, 1.63) \| Q1 \| Reference \| 1.36 (1.04, 1.78) \| 1.42 (1.19, 1.69) \| \| Q2 \| 1.18 (1.01, 1.37) \| 1.35 (1.06, 1.71) \| 1.84 (1.57, 2.16) \| Q2 \| 1.27 (1.09, 1.49) \| 1.54 (1.20, 1.97) \| 1.91 (1.62, 2.25) \| \| Q3 \| 0.77 (0.65, 0.90) \| 1.11 (0.85, 1.44) \| 1.21 (1.03, 1.43) \| Q3 \| 1.27 (1.09, 1.48) \| 1.54 (1.18, 2.01) \| 1.92 (1.63, 2.26) \| \| Q4 \| 1.74 (1.49, 2.03) \| 1.81 (1.41, 2.32) \| 2.31 (1.99, 2.69) \| Q4 \| 2.04 (1.74, 2.39) \| 2.12 (1.66, 2.71) \| 2.76 (2.37, 3.22) \| \| SO_2_ (µg/m^3^) \|  \|  \|  \| AQI* \|  \|  \|  \| \| Q1 \| Reference \| 1.23 (0.96, 1.56) \| 1.41 (1.20, 1.65) \| Q1 \| Reference \| 1.32 (1.03, 1.71) \| 1.44 (1.23, 1.70) \| \| Q2 \| 0.67 (0.57, 0.79) \| 0.90 (0.68, 1.18) \| 1.06 (0.89, 1.25) \| Q2 \| 0.83 (0.71, 0.97) \| 0.81 (0.61, 1.07) \| 1.25 (1.06, 1.48) \| \| Q3 \| 1.20 (1.03, 1.39) \| 1.45 (1.11, 1.89) \| 1.92 (1.64, 2.25) \| Q3 \| 1.00 (0.85, 1.16) \| 1.50 (1.17, 1.91) \| 1.58 (1.35, 1.86) \| \| Q4 \| 1.60 (1.37, 1.87) \| 1.61 (1.27, 2.05) \| 2.05 (1.76, 2.38) \| Q4 \| 1.62 (1.38, 1.89) \| 1.66 (1.30, 2.13) \| 2.11 (1.82, 2.46) \|   All model adjusted for sex, age, education, residence, marital status, household expenses per capita, smoke, drink, solid fuel usage, sleep duration, depressive symptoms, and non-communicable diseases. ^a^ Based on the WHO Guidelines on Physical Activity and Sedentary Behaviour 2020, inactive was considered as not participating MVPA. And we use the same METs as 300 minutes of moderate physical activity or 150 minutes of vigorous physical activity as the dividing line between insufficiently active and physically active. ^b^ The AQI is a numerical indicator used to assess air quality, based on the concentrations of multiple pollutants, including CO, PM_2.5_, SO_2_, NO_2_, O_3_, and PM_10_. CVD, cardiovascular diseases; MVPA, moderate-vigorous physical activity; CI, confidence interval; CO, Carbon monoxide; PM_1_, particulate matter with aerodynamic diameter ≤1µm; PM_2.5_, particulate matter with aerodynamic diameter ≤2.5µm; SO_2_, Sulfur dioxide; NO_2_, nitrogen dioxide; O_3_, Ozone; PM_10_, particulate matter with aerodynamic diameter ≤10µm; AQI, air quality index. |

| Supplemental table 8. Joint association of MVPA and air pollutants with the prevalence of CVD in females, CHARLS 2018. |
| --- |
| \|  \| Odd Ratio (95%CI) \| \| \|  \| Odd Ratio (95%CI) \| \| \| \| --- \| --- \| --- \| --- \| --- \| --- \| --- \| --- \| \|  \| Physically active^&^ \| Insufficiently active^&^ \| Inactive^&^ \|  \| Physically active^&^ \| Insufficiently active^&^ \| Inactive^&^ \| \| CO (µg/m^3^) \|  \|  \|  \| NO_2_ (µg/m^3^) \|  \|  \|  \| \| Q1 \| Reference \| 1.07 (0.79, 1.47) \| 1.34 (1.08, 1.65) \| Q1 \| Reference \| 1.08 (0.78, 1.50) \| 1.37 (1.11, 1.70) \| \| Q2 \| 0.69 (0.56, 0.85) \| 0.80 (0.55, 1.16) \| 1.03 (0.83, 1.28) \| Q2 \| 0.71 (0.58, 0.87) \| 1.05 (0.77, 1.43) \| 1.12 (0.90, 1.38) \| \| Q3 \| 0.67 (0.55, 0.83) \| 1.13 (0.83, 1.54) \| 1.14 (0.92, 1.42) \| Q3 \| 0.75 (0.62, 0.92) \| 0.94 (0.66, 1.33) \| 1.25 (1.01, 1.54) \| \| Q4 \| 1.38 (1.13, 1.69) \| 1.19 (0.87, 1.63) \| 1.66 (1.36, 2.03) \| Q4 \| 1.24 (1.01, 1.52) \| 1.19 (0.87, 1.61) \| 1.49 (1.22, 1.81) \| \| PM_1_ (µg/m^3^) \|  \|  \|  \| O_3_ (µg/m^3^) \|  \|  \|  \| \| Q1 \| Reference \| 1.36 (0.98, 1.90) \| 1.45 (1.17, 1.81) \| Q1 \| Reference \| 1.38 (1.00, 1.90) \| 1.62 (1.30, 2.01) \| \| Q2 \| 0.98 (0.80, 1.20) \| 0.93 (0.67, 1.29) \| 1.48 (1.18, 1.84) \| Q2 \| 1.11 (0.91, 1.35) \| 1.50 (1.11, 2.04) \| 1.47 (1.19, 1.83) \| \| Q3 \| 0.91 (0.74, 1.12) \| 1.38 (1.00, 1.89) \| 1.32 (1.07, 1.64) \| Q3 \| 1.25 (1.02, 1.53) \| 1.19 (0.85, 1.67) \| 1.96 (1.60, 2.42) \| \| Q4 \| 1.58 (1.28, 1.94) \| 1.53 (1.10, 2.11) \| 1.95 (1.60, 2.38) \| Q4 \| 1.39 (1.13, 1.72) \| 1.44 (1.03, 2.01) \| 1.73 (1.42, 2.12) \| \| PM_2.5_ (µg/m^3^) \|  \|  \|  \| PM_10_ (µg/m^3^) \|  \|  \|  \| \| Q1 \| Reference \| 1.25 (0.89, 1.75) \| 1.43 (1.14, 1.79) \| Q1 \| Reference \| 1.46 (1.04, 2.05) \| 1.46 (1.15, 1.86) \| \| Q2 \| 1.34 (1.09, 1.63) \| 1.41 (1.03, 1.93) \| 1.97 (1.59, 2.45) \| Q2 \| 1.41 (1.14, 1.73) \| 1.54 (1.12, 2.13) \| 1.97 (1.58, 2.46) \| \| Q3 \| 0.81 (0.66, 1.00) \| 1.09 (0.78, 1.53) \| 1.22 (0.98, 1.53) \| Q3 \| 1.55 (1.26, 1.91) \| 1.68 (1.20, 2.37) \| 2.14 (1.72, 2.67) \| \| Q4 \| 1.72 (1.40, 2.12) \| 1.85 (1.34, 2.54) \| 2.13 (1.74, 2.61) \| Q4 \| 2.01 (1.62, 2.50) \| 2.18 (1.58, 2.99) \| 2.65 (2.16, 3.26) \| \| SO_2_ (µg/m^3^) \|  \|  \|  \| AQI* \|  \|  \|  \| \| Q1 \| Reference \| 1.22 (0.90, 1.66) \| 1.34 (1.08, 1.67) \| Q1 \| Reference \| 1.22 (0.88, 1.68) \| 1.44 (1.16, 1.80) \| \| Q2 \| 0.65 (0.52, 0.80) \| 0.79 (0.56, 1.12) \| 0.90 (0.72, 1.13) \| Q2 \| 0.76 (0.62, 0.94) \| 0.75 (0.52, 1.08) \| 1.23 (0.98, 1.53) \| \| Q3 \| 1.16 (0.96, 1.42) \| 1.57 (1.12, 2.19) \| 1.87 (1.52, 2.30) \| Q3 \| 1.13 (0.92, 1.38) \| 1.64 (1.20, 2.23) \| 1.53 (1.24, 1.89) \| \| Q4 \| 1.47 (1.19, 1.82) \| 1.34 (0.98, 1.83) \| 1.82 (1.49, 2.22) \| Q4 \| 1.47 (1.19, 1.81) \| 1.47 (1.08, 2.02) \| 1.86 (1.53, 2.27) \|   All model adjusted for age, education, residence, marital status, household expenses per capita, smoke, drink, solid fuel usage, sleep duration, depressive symptoms, and non-communicable diseases. ^a^ Based on the WHO Guidelines on Physical Activity and Sedentary Behaviour 2020, inactive was considered as not participating MVPA. And we use the same METs as 300 minutes of moderate physical activity or 150 minutes of vigorous physical activity as the dividing line between insufficiently active and physically active. ^b^ The AQI is a numerical indicator used to assess air quality, based on the concentrations of multiple pollutants, including CO, PM_2.5_, SO_2_, NO_2_, O_3_, and PM_10_. CVD, cardiovascular diseases; MVPA, moderate-vigorous physical activity; CI, confidence interval; CO, Carbon monoxide; PM_1_, particulate matter with aerodynamic diameter ≤1µm; PM_2.5_, particulate matter with aerodynamic diameter ≤2.5µm; SO_2_, Sulfur dioxide; NO_2_, nitrogen dioxide; O_3_, Ozone; PM_10_, particulate matter with aerodynamic diameter ≤10µm; AQI, air quality index. |

| Supplemental table 9. Joint association of MVPA and air pollutants with the prevalence of CVD in males, CHARLS 2018. |  |
| --- | --- |
| \|  \| Odd Ratio (95%CI) \| \| \|  \| Odd Ratio (95%CI) \| \| \| \| --- \| --- \| --- \| --- \| --- \| --- \| --- \| --- \| \|  \| Physically active^&^ \| Insufficiently active^&^ \| Inactive^&^ \|  \| Physically active^&^ \| Insufficiently active^&^ \| Inactive^&^ \| \| CO (µg/m^3^) \|  \|  \|  \| NO_2_ (µg/m^3^) \|  \|  \|  \| \| Q1 \| Reference \| 1.17 (0.78, 1.75) \| 1.51 (1.18, 1.92) \| Q1 \| Reference \| 1.15 (0.74, 1.77) \| 1.57 (1.24, 1.98) \| \| Q2 \| 0.86 (0.67, 1.10) \| 0.99 (0.63, 1.55) \| 1.20 (0.94, 1.53) \| Q2 \| 0.91 (0.72, 1.15) \| 1.24 (0.84, 1.84) \| 1.31 (1.04, 1.66) \| \| Q3 \| 0.91 (0.72, 1.16) \| 1.13 (0.74, 1.73) \| 1.61 (1.26, 2.07) \| Q3 \| 0.88 (0.70, 1.10) \| 1.01 (0.66, 1.53) \| 1.48 (1.16, 1.88) \| \| Q4 \| 1.62 (1.29, 2.05) \| 2.17 (1.48, 3.16) \| 2.41 (1.91, 3.03) \| Q4 \| 1.38 (1.10, 1.74) \| 1.84 (1.26, 2.69) \| 2.08 (1.66, 2.61) \| \| PM_1_ (µg/m^3^) \|  \|  \|  \| O_3_ (µg/m^3^) \|  \|  \|  \| \| Q1 \| Reference \| 1.36 (0.88, 2.10) \| 1.54 (1.21, 1.96) \| Q1 \| Reference \| 1.67 (1.11, 2.51) \| 1.87 (1.46, 2.40) \| \| Q2 \| 0.93 (0.73, 1.18) \| 1.03 (0.69, 1.54) \| 1.34 (1.05, 1.71) \| Q2 \| 1.57 (1.23, 1.99) \| 1.11 (0.72, 1.73) \| 1.87 (1.46, 2.39) \| \| Q3 \| 0.90 (0.71, 1.15) \| 1.38 (0.92, 2.07) \| 1.55 (1.22, 1.98) \| Q3 \| 1.30 (1.02, 1.65) \| 2.27 (1.53, 3.37) \| 2.37 (1.86, 3.02) \| \| Q4 \| 1.85 (1.48, 2.33) \| 2.05 (1.38, 3.04) \| 2.69 (2.14, 3.38) \| Q4 \| 1.72 (1.35, 2.19) \| 2.04 (1.36, 3.07) \| 2.49 (1.96, 3.17) \| \| PM_2.5_ (µg/m^3^) \|  \|  \|  \| PM_10_ (µg/m^3^) \|  \|  \|  \| \| Q1 \| Reference \| 1.25 (0.81, 1.94) \| 1.35 (1.05, 1.73) \| Q1 \| Reference \| 1.20 (0.77, 1.90) \| 1.36 (1.05, 1.75) \| \| Q2 \| 1.03 (0.81, 1.30) \| 1.22 (0.83, 1.79) \| 1.71 (1.35, 2.16) \| Q2 \| 1.09 (0.85, 1.39) \| 1.52 (1.03, 2.24) \| 1.83 (1.44, 2.32) \| \| Q3 \| 0.71 (0.56, 0.91) \| 1.19 (0.78, 1.81) \| 1.21 (0.95, 1.55) \| Q3 \| 1.01 (0.79, 1.29) \| 1.47 (0.97, 2.24) \| 1.68 (1.31, 2.15) \| \| Q4 \| 1.78 (1.42, 2.23) \| 1.83 (1.23, 2.71) \| 2.60 (2.07, 3.26) \| Q4 \| 2.07 (1.64, 2.61) \| 2.03 (1.37, 3.00) \| 2.93 (2.32, 3.70) \| \| SO_2_ (µg/m^3^) \|  \|  \|  \| AQI* \|  \|  \|  \| \| Q1 \| Reference \| 1.18 (0.80, 1.77) \| 1.49 (1.17, 1.90) \| Q1 \| Reference \| 1.46 (0.95, 2.23) \| 1.47 (1.15, 1.89) \| \| Q2 \| 0.71 (0.55, 0.91) \| 1.11 (0.71, 1.73) \| 1.30 (1.01, 1.67) \| Q2 \| 0.93 (0.73, 1.19) \| 0.88 (0.57, 1.36) \| 1.30 (1.02, 1.67) \| \| Q3 \| 1.27 (1.00, 1.60) \| 1.21 (0.78, 1.90) \| 1.98 (1.55, 2.53) \| Q3 \| 0.89 (0.70, 1.14) \| 1.52 (1.03, 2.26) \| 1.72 (1.35, 2.19) \| \| Q4 \| 1.77 (1.40, 2.24) \| 2.20 (1.51, 3.19) \| 2.40 (1.89, 3.04) \| Q4 \| 1.85 (1.47, 2.34) \| 1.95 (1.31, 2.88) \| 2.56 (2.03, 3.23) \|   All model adjusted for age, education, residence, marital status, household expenses per capita, smoke, drink, solid fuel usage, sleep duration, depressive symptoms, and non-communicable diseases. ^a^ Based on the WHO Guidelines on Physical Activity and Sedentary Behaviour 2020, inactive was considered as not participating MVPA. And we use the same METs as 300 minutes of moderate physical activity or 150 minutes of vigorous physical activity as the dividing line between insufficiently active and physically active. ^b^ The AQI is a numerical indicator used to assess air quality, based on the concentrations of multiple pollutants, including CO, PM_2.5_, SO_2_, NO_2_, O_3_, and PM_10_. CVD, cardiovascular diseases; MVPA, moderate-vigorous physical activity; CI, confidence interval; CO, Carbon monoxide; PM_1_, particulate matter with aerodynamic diameter ≤1µm; PM_2.5_, particulate matter with aerodynamic diameter ≤2.5µm; SO_2_, Sulfur dioxide; NO_2_, nitrogen dioxide; O_3_, Ozone; PM_10_, particulate matter with aerodynamic diameter ≤10µm; AQI, air quality index. | |

| Supplemental table 10. Subgroup analysis on the associations between air pollutants and the prevalence of CVD stratified by MVPA categories, CHARLS 2018. |
| --- |
| \| High levels of air pollutants \| MVPA level \| \| P for interaction \| \| --- \| --- \| --- \| --- \| \| Low-medium \| High \| \| CO \| 1.55 (1.41, 1.70) \| 1.86 (1.52, 2.28) \| 0.106 \| \| PM_1_ \| 1.64 (1.50, 1.80) \| 1.86 (1.52, 2.28) \| 0.265 \| \| PM_2.5_ \| 1.63 (1.49, 1.79) \| 1.86 (1.52, 2.27) \| 0.252 \| \| SO_2_ \| 1.48 (1.35, 1.63) \| 1.82 (1.48, 2.23) \| 0.073 \| \| NO_2_ \| 1.34 (1.22, 1.47) \| 1.57 (1.27, 1.94) \| 0.182 \| \| O_3_ \| 1.18 (1.07, 1.30) \| 1.29 (1.05, 1.60) \| 0.439 \| \| PM_10_ \| 1.63 (1.49, 1.79) \| 1.85 (1.50, 2.27) \| 0.284 \| \| AQI \| 1.57 (1.43, 1.72) \| 1.86 (1.51, 2.28) \| 0.144 \|   P<0.05 was considered statistically significant. The cut off point of air pollutants and moderate-vigorous physical activity are cohort-specific cut-off points at the 75%. Multivariable logistic regression model was used to examine the associations, which were adjusted for sex, age, education, residence, marital status, household expenses per capita, smoke, drink, solid fuel usage, sleep duration, depressive symptoms, and non-communicable diseases. The results expressed as odd ratios (OR) and 95% confidence intervals (CI). CVD, cardiovascular diseases; MVPA, moderate-vigorous physical activity; CO, Carbon monoxide; PM_1_, particulate matter with aerodynamic diameter ≤1µm; PM_2.5_, particulate matter with aerodynamic diameter ≤2.5µm; SO_2_, Sulfur dioxide; NO_2_, nitrogen dioxide; O_3_, Ozone; PM_10_, particulate matter with aerodynamic diameter ≤10µm; AQI, air quality index. |
|  |
| Supplemental table 11. Subgroup analysis on the associations between air pollutants and the prevalence of CVD stratified by MVPA categories in females, CHARLS 2018.   \| Air pollution (high) \| MVPA level \| \| P for interaction \| \| --- \| --- \| --- \| --- \| \| Low-medium \| High \| \| CO \| 1.50 (1.33, 1.70) \| 1.78 (1.33, 2.38) \| 0.288 \| \| PM_1_ \| 1.51 (1.34, 1.71) \| 1.65 (1.22, 2.21) \| 0.603 \| \| PM_2.5_ \| 1.52 (1.34, 1.72) \| 1.68 (1.25, 2.25) \| 0.543 \| \| SO_2_ \| 1.41 (1.24, 1.59) \| 1.65 (1.22, 2.22) \| 0.342 \| \| NO_2_ \| 1.29 (1.14, 1.46) \| 1.40 (1.02, 1.91) \| 0.650 \| \| O_3_ \| 1.12 (0.98, 1.27) \| 1.16 (0.85, 1.58) \| 0.834 \| \| PM_10_ \| 1.52 (1.34, 1.72) \| 1.55 (1.14, 2.10) \| 0.909 \| \| AQI \| 1.42 (1.25, 1.60) \| 1.45 (1.07, 1.97) \| 0.889 \|   P<0.05 was considered statistically significant. The cut off point of air pollutants and moderate-vigorous physical activity are cohort-specific cut-off points at the 75%. Multivariable logistic regression model was used to examine the associations, which were adjusted for age, education, residence, marital status, household expenses per capita, smoke, drink, solid fuel usage, sleep duration, depressive symptoms, and non-communicable diseases. The results expressed as odd ratios (OR) and 95% confidence intervals (CI). CVD, cardiovascular diseases; MVPA, moderate-vigorous physical activity; CO, Carbon monoxide; PM_1_, particulate matter with aerodynamic diameter ≤1µm; PM_2.5_, particulate matter with aerodynamic diameter ≤2.5µm; SO_2_, Sulfur dioxide; NO_2_, nitrogen dioxide; O_3_, Ozone; PM_10_, particulate matter with aerodynamic diameter ≤10µm; AQI, air quality index. |
| Supplemental table 12. Subgroup analysis on the associations between air pollutants and the prevalence of CVD stratified by MVPA categories in males, CHARLS 2018. |
| \| High levels of air pollutants \| MVPA level \| \| P for interaction \| \| --- \| --- \| --- \| --- \| \| Low-medium \| High \| \| CO \| 1.64 (1.43, 1.89) \| 2.20 (1.64, 2.94) \| 0.077 \| \| PM_1_ \| 1.81 (1.57, 2.08) \| 2.36 (1.77, 3.16) \| 0.098 \| \| PM_2.5_ \| 1.81 (1.57, 2.08) \| 2.42 (1.81, 3.23) \| 0.073 \| \| SO_2_ \| 1.55 (1.35, 1.79) \| 2.31 (1.73, 3.10) \| 0.015 \| \| NO_2_ \| 1.38 (1.20, 1.59) \| 2.00 (1.48, 2.70) \| 0.029 \| \| O_3_ \| 1.24 (1.08, 1.43) \| 1.61 (1.20, 2.17) \| 0.118 \| \| PM_10_ \| 1.78 (1.55, 2.04) \| 2.44 (1.83, 3.26) \| 0.051 \| \| AQI \| 1.71 (1.49, 1.96) \| 2.50 (1.87, 3.35) \| 0.019 \|   P<0.05 was considered statistically significant. The cut off point of air pollutants and moderate-vigorous physical activity are cohort-specific cut-off points at the 75%. Multivariable logistic regression model was used to examine the associations, which were adjusted for age, education, residence, marital status, household expenses per capita, smoke, drink, solid fuel usage, sleep duration, depressive symptoms, and non-communicable diseases. The results expressed as odd ratios (OR) and 95% confidence intervals (CI). CVD, cardiovascular diseases; MVPA, moderate-vigorous physical activity; CO, Carbon monoxide; PM_1_, particulate matter with aerodynamic diameter ≤1µm; PM_2.5_, particulate matter with aerodynamic diameter ≤2.5µm; SO_2_, Sulfur dioxide; NO_2_, nitrogen dioxide; O_3_, Ozone; PM_10_, particulate matter with aerodynamic diameter ≤10µm; AQI, air quality index. |

|  |
| --- |
| \| Supplemental table 13. Dose–response associations of MVPA with the prevalence of CVD stratified by air pollutants concentration, CHARLS 2018. \| \| \| \| \| \| \| --- \| --- \| --- \| --- \| --- \| --- \| \| Air pollutants \| P for overall (low-medium) \| P for nonlinear (low-medium) \| P for overall (high) \| P for nonlinear (high) \| P for interaction \| \| Duration \|  \|  \|  \|  \|  \| \| CO \| <0.001 \| 0.006 \| 0.007 \| 0.125 \| 0.168 \| \| PM_1_ \| <0.001 \| 0.017 \| 0.004 \| 0.076 \| 0.099 \| \| PM_2.5_ \| <0.001 \| 0.020 \| 0.003 \| 0.059 \| 0.078 \| \| SO_2_ \| <0.001 \| 0.039 \| 0.011 \| 0.017 \| 0.003 \| \| NO_2_ \| <0.001 \| 0.014 \| 0.002 \| 0.146 \| 0.095 \| \| O_3_ \| <0.001 \| 0.012 \| 0.004 \| 0.132 \| 0.067 \| \| PM_10_ \| <0.001 \| 0.020 \| 0.005 \| 0.063 \| 0.114 \| \| AQI \| <0.001 \| 0.013 \| 0.016 \| 0.076 \| 0.039 \| \|  \|  \|  \|  \|  \|  \| \| METs \|  \|  \|  \|  \|  \| \| CO \| <0.001 \| 0.002 \| 0.003 \| 0.032 \| 0.122 \| \| PM_1_ \| <0.001 \| 0.003 \| 0.002 \| 0.032 \| 0.080 \| \| PM_2.5_ \| <0.001 \| 0.005 \| 0.001 \| 0.017 \| 0.056 \| \| SO_2_ \| <0.001 \| 0.008 \| 0.007 \| 0.009 \| 0.003 \| \| NO_2_ \| <0.001 \| 0.005 \| 0.001 \| 0.024 \| 0.057 \| \| O_3_ \| <0.001 \| 0.002 \| 0.002 \| 0.080 \| 0.086 \| \| PM_10_ \| <0.001 \| 0.006 \| 0.002 \| 0.011 \| 0.047 \| \| AQI \| <0.001 \| 0.003 \| 0.008 \| 0.022 \| 0.024 \| |
| P<0.05 was considered statistically significant. The cut off point of air pollutants and moderate-vigorous physical activity are cohort-specific cut-off points at the 75%. All models were adjusted for was used to examine the associations, which were adjusted for sex, age, education, residence, marital status, household expenses per capita, smoke, drink, solid fuel usage, sleep duration, depressive symptoms, and non-communicable diseases. CVD, cardiovascular diseases; MVPA, moderate-vigorous physical activity; METs, Metabolic equivalents; CO, Carbon monoxide; PM_1_, particulate matter with aerodynamic diameter ≤1µm; PM_2.5_, particulate matter with aerodynamic diameter ≤2.5µm; SO_2_, Sulfur dioxide; NO_2_, nitrogen dioxide; O_3_, Ozone; PM_10_, particulate matter with aerodynamic diameter ≤10µm; AQI, air quality index. |

| \| Supplemental table 14. Dose–response associations of MVPA with the prevalence of CVD stratified by air pollutants concentration in females, CHARLS 2018. \| \| \| \| \| \| \| --- \| --- \| --- \| --- \| --- \| --- \| \| Air pollutants \| P for overall (low-medium) \| P for nonlinear (low-medium) \| P for overall (high) \| P for nonlinear (high) \| P for interaction \| \| Duration \|  \|  \|  \|  \|  \| \| CO \| <0.001 \| 0.446 \| 0.627 \| 0.551 \| 0.031 \| \| PM_1_ \| <0.001 \| 0.559 \| 0.212 \| 0.329 \| 0.152 \| \| PM_2.5_ \| <0.001 \| 0.555 \| 0.271 \| 0.371 \| 0.103 \| \| SO_2_ \| <0.001 \| 0.523 \| 0.160 \| 0.090 \| 0.018 \| \| NO_2_ \| <0.001 \| 0.124 \| 0.216 \| 0.709 \| 0.156 \| \| O_3_ \| <0.001 \| 0.186 \| 0.189 \| 0.382 \| 0.090 \| \| PM_10_ \| <0.001 \| 0.609 \| 0.131 \| 0.207 \| 0.212 \| \| AQI \| <0.001 \| 0.411 \| 0.230 \| 0.353 \| 0.182 \| \|  \|  \|  \|  \|  \|  \| \| METs \|  \|  \|  \|  \|  \| \| CO \| <0.001 \| 0.200 \| 0.441 \| 0.217 \| 0.009 \| \| PM_1_ \| <0.001 \| 0.300 \| 0.143 \| 0.103 \| 0.069 \| \| PM_2.5_ \| <0.001 \| 0.317 \| 0.183 \| 0.123 \| 0.048 \| \| SO_2_ \| <0.001 \| 0.381 \| 0.055 \| 0.017 \| 0.002 \| \| NO_2_ \| <0.001 \| 0.058 \| 0.106 \| 0.110 \| 0.047 \| \| O_3_ \| <0.001 \| 0.101 \| 0.168 \| 0.190 \| 0.064 \| \| PM_10_ \| <0.001 \| 0.447 \| 0.052 \| 0.026 \| 0.041 \| \| AQI \| <0.001 \| 0.277 \| 0.111 \| 0.062 \| 0.048 \| |
| --- | --- | --- | --- | --- | --- | --- | --- | --- | --- | --- | --- | --- | --- | --- | --- | --- | --- | --- | --- | --- | --- | --- | --- | --- | --- | --- | --- | --- | --- | --- | --- | --- | --- | --- | --- | --- | --- | --- | --- | --- | --- | --- | --- | --- | --- | --- | --- | --- | --- | --- | --- | --- | --- | --- | --- | --- | --- | --- | --- | --- | --- | --- | --- | --- | --- | --- | --- | --- | --- | --- | --- | --- | --- | --- | --- | --- | --- | --- | --- | --- | --- | --- | --- | --- | --- | --- | --- | --- | --- | --- | --- | --- | --- | --- | --- | --- | --- | --- | --- | --- | --- | --- | --- | --- | --- | --- | --- | --- | --- | --- | --- | --- | --- | --- | --- | --- | --- | --- | --- | --- | --- | --- | --- | --- | --- | --- |
| P<0.05 was considered statistically significant. The cut off point of air pollutants and moderate-vigorous physical activity are cohort-specific cut-off points at the 75%. All models were adjusted for was used to examine the associations, which were adjusted for age, education, residence, marital status, household expenses per capita, smoke, drink, solid fuel usage, sleep duration, depressive symptoms, and non-communicable diseases. CVD, cardiovascular diseases; MVPA, moderate-vigorous physical activity; METs, Metabolic equivalents; CO, Carbon monoxide; PM_1_, particulate matter with aerodynamic diameter ≤1µm; PM_2.5_, particulate matter with aerodynamic diameter ≤2.5µm; SO_2_, Sulfur dioxide; NO_2_, nitrogen dioxide; O_3_, Ozone; PM_10_, particulate matter with aerodynamic diameter ≤10µm; AQI, air quality index. |

| \| Supplemental table 15. Dose–response associations of MVPA with the prevalence of CVD stratified by air pollutants concentration in males, CHARLS 2018. \| \| \| \| \| \| \| --- \| --- \| --- \| --- \| --- \| --- \| \| Air pollutants \| P for overall (low-medium) \| P for nonlinear (low-medium) \| P for overall (high) \| P for nonlinear (high) \| P for interaction \| \| Duration \|  \|  \|  \|  \|  \| \| CO \| <0.001 \| 0.002 \| 0.011 \| 0.127 \| 0.735 \| \| PM_1_ \| <0.001 \| 0.005 \| 0.017 \| 0.141 \| 0.591 \| \| PM_2.5_ \| <0.001 \| 0.004 \| 0.021 \| 0.104 \| 0.455 \| \| SO_2_ \| <0.001 \| 0.008 \| 0.064 \| 0.064 \| 0.130 \| \| NO_2_ \| <0.001 \| 0.009 \| 0.007 \| 0.105 \| 0.566 \| \| O_3_ \| <0.001 \| 0.003 \| 0.028 \| 0.228 \| 0.547 \| \| PM_10_ \| <0.001 \| 0.003 \| 0.044 \| 0.201 \| 0.368 \| \| AQI \| <0.001 \| 0.003 \| 0.062 \| 0.155 \| 0.267 \| \|  \|  \|  \|  \|  \|  \| \| METs \|  \|  \|  \|  \|  \| \| CO \| <0.001 \| 0.003 \| 0.005 \| 0.082 \| 0.865 \| \| PM_1_ \| <0.001 \| 0.002 \| 0.010 \| 0.153 \| 0.666 \| \| PM_2.5_ \| <0.001 \| 0.003 \| 0.012 \| 0.088 \| 0.532 \| \| SO_2_ \| <0.001 \| 0.003 \| 0.047 \| 0.116 \| 0.275 \| \| NO_2_ \| <0.001 \| 0.006 \| 0.004 \| 0.098 \| 0.635 \| \| O_3_ \| <0.001 \| 0.002 \| 0.010 \| 0.222 \| 0.715 \| \| PM_10_ \| <0.001 \| 0.002 \| 0.031 \| 0.174 \| 0.422 \| \| AQI \| <0.001 \| 0.002 \| 0.044 \| 0.148 \| 0.336 \| |
| --- | --- | --- | --- | --- | --- | --- | --- | --- | --- | --- | --- | --- | --- | --- | --- | --- | --- | --- | --- | --- | --- | --- | --- | --- | --- | --- | --- | --- | --- | --- | --- | --- | --- | --- | --- | --- | --- | --- | --- | --- | --- | --- | --- | --- | --- | --- | --- | --- | --- | --- | --- | --- | --- | --- | --- | --- | --- | --- | --- | --- | --- | --- | --- | --- | --- | --- | --- | --- | --- | --- | --- | --- | --- | --- | --- | --- | --- | --- | --- | --- | --- | --- | --- | --- | --- | --- | --- | --- | --- | --- | --- | --- | --- | --- | --- | --- | --- | --- | --- | --- | --- | --- | --- | --- | --- | --- | --- | --- | --- | --- | --- | --- | --- | --- | --- | --- | --- | --- | --- | --- | --- | --- | --- | --- | --- | --- |
| P<0.05 was considered statistically significant. The cut off point of air pollutants and moderate-vigorous physical activity are cohort-specific cut-off points at the 75%. All models were adjusted for was used to examine the associations, which were adjusted for age, education, residence, marital status, household expenses per capita, smoke, drink, solid fuel usage, sleep duration, depressive symptoms, and non-communicable diseases. CVD, cardiovascular diseases; MVPA, moderate-vigorous physical activity; METs, Metabolic equivalents; CO, Carbon monoxide; PM_1_, particulate matter with aerodynamic diameter ≤1µm; PM_2.5_, particulate matter with aerodynamic diameter ≤2.5µm; SO_2_, Sulfur dioxide; NO_2_, nitrogen dioxide; O_3_, Ozone; PM_10_, particulate matter with aerodynamic diameter ≤10µm; AQI, air quality index. |

| Supplemental table 16. Path data of the example in which MVPA is hypothesized as a mediator of the relation between air pollutants and the prevalence of CVD, CHARLS 2018. | | | |
| --- | --- | --- | --- |
| Air pollution | ADE (average) | ACME (average) | Proportion of mediation  (average) |
| CO | 0.056870*** | 0.008830*** | 13.52%*** |
| PM_1_ | 0.001692*** | 0.000272*** | 13.88%*** |
| PM_2.5_ | 0.001257*** | 0.000155*** | 10.90%*** |
| SO_2_ | 0.002628*** | 0.000332*** | 11.18%*** |
| NO_2_ | 0.001180*** | 0.000234*** | 16.43%*** |
| O_3_ | 0.000802*** | 0.000153*** | 15.81%*** |
| PM_10_ | 0.000884*** | 0.000075*** | 7.80%*** |
| AQI | 0.000864*** | 0.000092*** | 9.57%*** |
| *** p<0.001, ** p<0.01, * p<0.05. Multivariable logistic regression model was used to examine the associations, which were adjusted for sex, age, education, residence, marital status, household expenses per capita, smoke, drink, solid fuel usage, sleep duration, depressive symptoms, and non-communicable diseases. CVD, cardiovascular diseases; MVPA, moderate-vigorous physical activity; CO, Carbon monoxide; PM_1_, particulate matter with aerodynamic diameter ≤1µm; PM_2.5_, particulate matter with aerodynamic diameter ≤2.5µm; SO_2_, Sulfur dioxide; NO_2_, nitrogen dioxide; O_3_, Ozone; PM_10_, particulate matter with aerodynamic diameter ≤10µm; AQI, air quality index; ACME, average causal mediation effects; ADE, average direct effects. | | | |
| Supplemental table 17. Path data of the example in which MVPA is hypothesized as a mediator of the relation between air pollutants and the prevalence of CVD in females, CHARLS 2018. | | | |
| Air pollution | ADE (average) | ACME (average) | Proportion of mediation  (average) |
| CO | 0.025700 | 0.012990*** | 32.38%* |
| PM_1_ | 0.001030 | 0.000398*** | 27.10%* |
| PM_2.5_ | 0.001049*** | 0.000220*** | 16.96%*** |
| SO_2_ | 0.002000*** | 0.000449*** | 18.39%*** |
| NO_2_ | 0.000617 | 0.000330*** | 33.30%* |
| O_3_ | 0.000625 | 0.000262*** | 28.00%** |
| PM_10_ | 0.000891*** | 0.000102*** | 10.10%*** |
| AQI | 0.000820*** | 0.000126*** | 13.10%*** |
| *** p<0.001, ** p<0.01, * p<0.05. Multivariable logistic regression model was used to examine the associations, which were adjusted for age, education, residence, marital status, household expenses per capita, smoke, drink, solid fuel usage, sleep duration, depressive symptoms, and non-communicable diseases. CVD, cardiovascular diseases; MVPA, moderate-vigorous physical activity; CO, Carbon monoxide; PM_1_, particulate matter with aerodynamic diameter ≤1µm; PM_2.5_, particulate matter with aerodynamic diameter ≤2.5µm; SO_2_, Sulfur dioxide; NO_2_, nitrogen dioxide; O_3_, Ozone; PM_10_, particulate matter with aerodynamic diameter ≤10µm; AQI, air quality index; ACME, average causal mediation effects; ADE, average direct effects; CESD. | | | |
|  | | | |
| Supplemental table 18. Path data of the example in which MVPA is hypothesized as a mediator of the relation between air pollutants and the prevalence of CVD in males, CHARLS 2018. | | | |
| Air pollution | ADE (average) | ACME (average) | Proportion of mediation  (average) |
| CO | 0.089030*** | 0.005280*** | 5.49%*** |
| PM_1_ | 0.002300*** | 0.000158*** | 6.32%*** |
| PM_2.5_ | 0.001420*** | 0.000095*** | 6.18%*** |
| SO_2_ | 0.003052*** | 0.000230*** | 6.92%*** |
| NO_2_ | 0.001680*** | 0.000140*** | 7.53%*** |
| O_3_ | 0.000802*** | 0.000068*** | 7.44%*** |
| PM_10_ | 0.000877*** | 0.000049*** | 5.14%*** |
| AQI | 0.000880*** | 0.000057*** | 6.02%*** |
| *** p<0.001, ** p<0.01, * p<0.05. Multivariable logistic regression model was used to examine the associations, which were adjusted for age, education, residence, marital status, household expenses per capita, smoke, drink, solid fuel usage, sleep duration, depressive symptoms, and non-communicable diseases. CVD, cardiovascular diseases; MVPA, moderate-vigorous physical activity; CO, Carbon monoxide; PM_1_, particulate matter with aerodynamic diameter ≤1µm; PM_2.5_, particulate matter with aerodynamic diameter ≤2.5µm; SO_2_, Sulfur dioxide; NO_2_, nitrogen dioxide; O_3_, Ozone; PM_10_, particulate matter with aerodynamic diameter ≤10µm; AQI, air quality index; ACME, average causal mediation effects; ADE, average direct effects. | | | |

| Supplemental table 19. Joint association of MVPA and air pollutants with the prevalence of CVD, CHARLS 2018 (adjusted for additional covariates). |  |  |
| --- | --- | --- |
| \|  \| Odd Ratio (95%CI) \| \| \|  \| Odd Ratio (95%CI) \| \| \| \| --- \| --- \| --- \| --- \| --- \| --- \| --- \| --- \| \|  \| Physically active^&^ \| Insufficiently active^&^ \| Inactive^&^ \|  \| Physically active^&^ \| Insufficiently active^&^ \| Inactive^&^ \| \| CO (µg/m^3^) \|  \|  \|  \| NO_2_ (µg/m^3^) \|  \|  \|  \| \| Q1 \| Reference \| 1.10 (0.86, 1.42) \| 1.39 (1.17, 1.63) \| Q1 \| Reference \| 1.15 (0.88, 1.52) \| 1.46 (1.24, 1.72) \| \| Q2 \| 0.74 (0.63, 0.87) \| 0.81 (0.60, 1.09) \| 1.11 (0.94, 1.31) \| Q2 \| 0.80 (0.69, 0.94) \| 1.11 (0.87, 1.43) \| 1.21 (1.03, 1.43) \| \| Q3 \| 0.77 (0.66, 0.91) \| 1.12 (0.86, 1.46) \| 1.27 (1.08, 1.51) \| Q3 \| 0.83 (0.71, 0.97) \| 0.89 (0.67, 1.18) \| 1.37 (1.16, 1.61) \| \| Q4 \| 1.42 (1.21, 1.66) \| 1.48 (1.15, 1.91) \| 1.94 (1.66, 2.27) \| Q4 \| 1.29 (1.10, 1.51) \| 1.45 (1.13, 1.86) \| 1.73 (1.49, 2.02) \| \| PM_1_ (µg/m^3^) \|  \|  \|  \| O_3_ (µg/m^3^) \|  \|  \|  \| \| Q1 \| Reference \| 1.38 (1.05, 1.81) \| 1.49 (1.26, 1.76) \| Q1 \| Reference \| 1.45 (1.11, 1.88) \| 1.71 (1.44, 2.02) \| \| Q2 \| 0.92 (0.78, 1.08) \| 0.89 (0.68, 1.16) \| 1.35 (1.14, 1.60) \| Q2 \| 1.24 (1.06, 1.46) \| 1.30 (1.00, 1.70) \| 1.59 (1.35, 1.88) \| \| Q3 \| 0.90 (0.76, 1.05) \| 1.31 (1.01, 1.70) \| 1.37 (1.16, 1.62) \| Q3 \| 1.22 (1.04, 1.43) \| 1.47 (1.12, 1.93) \| 2.06 (1.75, 2.43) \| \| Q4 \| 1.67 (1.42, 1.95) \| 1.72 (1.33, 2.23) \| 2.27 (1.94, 2.65) \| Q4 \| 1.49 (1.27, 1.75) \| 1.64 (1.26, 2.14) \| 1.99 (1.70, 2.34) \| \| PM_2.5_ (µg/m^3^) \|  \|  \|  \| PM_10_ (µg/m^3^) \|  \|  \|  \| \| Q1 \| Reference \| 1.27 (0.96, 1.68) \| 1.38 (1.17, 1.64) \| Q1 \| Reference \| 1.33 (1.00, 1.76) \| 1.42 (1.19, 1.70) \| \| Q2 \| 1.16 (0.99, 1.36) \| 1.23 (0.96, 1.59) \| 1.78 (1.51, 2.10) \| Q2 \| 1.25 (1.06, 1.47) \| 1.43 (1.10, 1.85) \| 1.88 (1.59, 2.23) \| \| Q3 \| 0.74 (0.63, 0.88) \| 1.05 (0.79, 1.38) \| 1.20 (1.01, 1.42) \| Q3 \| 1.27 (1.08, 1.49) \| 1.56 (1.19, 2.06) \| 1.88 (1.59, 2.24) \| \| Q4 \| 1.73 (1.47, 2.03) \| 1.81 (1.40, 2.34) \| 2.34 (2.00, 2.73) \| Q4 \| 2.04 (1.73, 2.41) \| 2.12 (1.64, 2.75) \| 2.82 (2.41, 3.31) \| \| SO_2_ (µg/m^3^) \|  \|  \|  \| AQI* \|  \|  \|  \| \| Q1 \| Reference \| 1.17 (0.91, 1.51) \| 1.44 (1.22, 1.70) \| Q1 \| Reference \| 1.36 (1.04, 1.77) \| 1.44 (1.22, 1.71) \| \| Q2 \| 0.66 (0.55, 0.78) \| 0.85 (0.64, 1.13) \| 1.02 (0.86, 1.22) \| Q2 \| 0.85 (0.72, 1.00) \| 0.79 (0.60, 1.06) \| 1.28 (1.08, 1.52) \| \| Q3 \| 1.18 (1.01, 1.38) \| 1.35 (1.02, 1.78) \| 1.88 (1.60, 2.22) \| Q3 \| 1.04 (0.88, 1.22) \| 1.45 (1.12, 1.87) \| 1.62 (1.38, 1.92) \| \| Q4 \| 1.59 (1.35, 1.86) \| 1.69 (1.32, 2.18) \| 2.03 (1.74, 2.38) \| Q4 \| 1.63 (1.39, 1.92) \| 1.74 (1.35, 2.24) \| 2.19 (1.87, 2.56) \|   All model adjusted for sex, age, education, residence, marital status, household expenses per capita, smoke, drink, solid fuel usage, sleep duration, depressive symptoms, non-communicable diseases, installation of residential air cleaner, and residential temperature. ^a^ Based on the WHO Guidelines on Physical Activity and Sedentary Behaviour 2020, inactive was considered as not participating MVPA. And we use the same METs as 300 minutes of moderate physical activity or 150 minutes of vigorous physical activity as the dividing line between insufficiently active and physically active. ^b^ The AQI is a numerical indicator used to assess air quality, based on the concentrations of multiple pollutants, including CO, PM_2.5_, SO_2_, NO_2_, O_3_, and PM_10_. CVD, cardiovascular diseases; MVPA, moderate-vigorous physical activity; CI, confidence interval; CO, Carbon monoxide; PM_1_, particulate matter with aerodynamic diameter ≤1µm; PM_2.5_, particulate matter with aerodynamic diameter ≤2.5µm; SO_2_, Sulfur dioxide; NO_2_, nitrogen dioxide; O_3_, Ozone; PM_10_, particulate matter with aerodynamic diameter ≤10µm; AQI, air quality index. | | |

| Supplemental table 20. Joint association of MVPA and air pollutants with the prevalence of CVD in females, CHARLS 2018 (adjusted for additional covariates). |  |  |
| --- | --- | --- |
| \|  \| Odd Ratio (95%CI) \| \| \|  \| Odd Ratio (95%CI) \| \| \| \| --- \| --- \| --- \| --- \| --- \| --- \| --- \| --- \| \|  \| Physically active^&^ \| Insufficiently active^&^ \| Inactive^&^ \|  \| Physically active^&^ \| Insufficiently active^&^ \| Inactive^&^ \| \| CO (µg/m^3^) \|  \|  \|  \| NO_2_ (µg/m^3^) \|  \|  \|  \| \| Q1 \| Reference \| 1.15 (0.83, 1.57) \| 1.39 (1.12, 1.74) \| Q1 \| Reference \| 1.17 (0.84, 1.64) \| 1.42 (1.14, 1.76) \| \| Q2 \| 0.69 (0.56, 0.85) \| 0.80 (0.55, 1.18) \| 1.06 (0.85, 1.33) \| Q2 \| 0.73 (0.59, 0.90) \| 1.04 (0.76, 1.44) \| 1.16 (0.93, 1.45) \| \| Q3 \| 0.69 (0.56, 0.86) \| 1.06 (0.76, 1.47) \| 1.14 (0.91, 1.42) \| Q3 \| 0.79 (0.64, 0.97) \| 0.89 (0.62, 1.28) \| 1.34 (1.08, 1.66) \| \| Q4 \| 1.34 (1.09, 1.66) \| 1.23 (0.88, 1.70) \| 1.67 (1.35, 2.05) \| Q4 \| 1.24 (1.00, 1.54) \| 1.28 (0.93, 1.76) \| 1.49 (1.21, 1.83) \| \| PM_1_ (µg/m^3^) \|  \|  \|  \| O_3_ (µg/m^3^) \|  \|  \|  \| \| Q1 \| Reference \| 1.40 (1.00, 1.98) \| 1.48 (1.18, 1.86) \| Q1 \| Reference \| 1.38 (0.99, 1.92) \| 1.64 (1.31, 2.06) \| \| Q2 \| 0.95 (0.77, 1.18) \| 0.88 (0.63, 1.24) \| 1.43 (1.14, 1.80) \| Q2 \| 1.06 (0.86, 1.30) \| 1.44 (1.04, 1.99) \| 1.50 (1.20, 1.88) \| \| Q3 \| 0.90 (0.73, 1.12) \| 1.34 (0.96, 1.87) \| 1.31 (1.04, 1.64) \| Q3 \| 1.25 (1.01, 1.54) \| 1.23 (0.86, 1.75) \| 1.94 (1.56, 2.40) \| \| Q4 \| 1.53 (1.23, 1.90) \| 1.59 (1.14, 2.23) \| 2.00 (1.62, 2.45) \| Q4 \| 1.36 (1.09, 1.69) \| 1.45 (1.03, 2.04) \| 1.71 (1.39, 2.11) \| \| PM_2.5_ (µg/m^3^) \|  \|  \|  \| PM_10_ (µg/m^3^) \|  \|  \|  \| \| Q1 \| Reference \| 1.34 (0.94, 1.90) \| 1.46 (1.16, 1.85) \| Q1 \| Reference \| 1.46 (1.03, 2.07) \| 1.54 (1.20, 1.96) \| \| Q2 \| 1.32 (1.07, 1.62) \| 1.31 (0.94, 1.81) \| 1.93 (1.54, 2.42) \| Q2 \| 1.38 (1.11, 1.72) \| 1.59 (1.13, 2.23) \| 1.95 (1.54, 2.46) \| \| Q3 \| 0.79 (0.64, 0.99) \| 1.06 (0.75, 1.51) \| 1.21 (0.96, 1.52) \| Q3 \| 1.57 (1.27, 1.95) \| 1.65 (1.16, 2.34) \| 2.19 (1.74, 2.76) \| \| Q4 \| 1.68 (1.35, 2.09) \| 1.93 (1.38, 2.70) \| 2.20 (1.79, 2.72) \| Q4 \| 2.02 (1.61, 2.54) \| 2.30 (1.65, 3.21) \| 2.73 (2.21, 3.39) \| \| SO_2_ (µg/m^3^) \|  \|  \|  \| AQI* \|  \|  \|  \| \| Q1 \| Reference \| 1.24 (0.90, 1.71) \| 1.42 (1.13, 1.78) \| Q1 \| Reference \| 1.28 (0.91, 1.79) \| 1.49 (1.19, 1.88) \| \| Q2 \| 0.64 (0.52, 0.80) \| 0.78 (0.55, 1.12) \| 0.89 (0.70, 1.13) \| Q2 \| 0.82 (0.66, 1.01) \| 0.78 (0.53, 1.13) \| 1.28 (1.02, 1.61) \| \| Q3 \| 1.14 (0.93, 1.40) \| 1.51 (1.06, 2.14) \| 1.90 (1.53, 2.36) \| Q3 \| 1.17 (0.95, 1.44) \| 1.60 (1.15, 2.22) \| 1.65 (1.32, 2.06) \| \| Q4 \| 1.46 (1.17, 1.81) \| 1.39 (1.01, 1.93) \| 1.78 (1.45, 2.19) \| Q4 \| 1.51 (1.21, 1.88) \| 1.67 (1.20, 2.31) \| 1.95 (1.58, 2.40) \|   All model adjusted for age, education, residence, marital status, household expenses per capita, smoke, drink, solid fuel usage, sleep duration, depressive symptoms, non-communicable diseases, installation of residential air cleaner, and residential temperature. ^a^ Based on the WHO Guidelines on Physical Activity and Sedentary Behaviour 2020, inactive was considered as not participating MVPA. And we use the same METs as 300 minutes of moderate physical activity or 150 minutes of vigorous physical activity as the dividing line between insufficiently active and physically active. ^b^ The AQI is a numerical indicator used to assess air quality, based on the concentrations of multiple pollutants, including CO, PM_2.5_, SO_2_, NO_2_, O_3_, and PM_10_. CVD, cardiovascular diseases; MVPA, moderate-vigorous physical activity; CI, confidence interval; CO, Carbon monoxide; PM_1_, particulate matter with aerodynamic diameter ≤1µm; PM_2.5_, particulate matter with aerodynamic diameter ≤2.5µm; SO_2_, Sulfur dioxide; NO_2_, nitrogen dioxide; O_3_, Ozone; PM_10_, particulate matter with aerodynamic diameter ≤10µm; AQI, air quality index. | | |

| Supplemental table 21. Joint association of MVPA and air pollutants with the prevalence of CVD in males, CHARLS 2018 (adjusted for additional covariates). |  |  |
| --- | --- | --- |
| \|  \| Odd Ratio (95%CI) \| \| \|  \| Odd Ratio (95%CI) \| \| \| \| --- \| --- \| --- \| --- \| --- \| --- \| --- \| --- \| \|  \| Physically active^&^ \| Insufficiently active^&^ \| Inactive^&^ \|  \| Physically active^&^ \| Insufficiently active^&^ \| Inactive^&^ \| \| CO (µg/m^3^) \|  \|  \|  \| NO_2_ (µg/m^3^) \|  \|  \|  \| \| Q1 \| Reference \| 1.00 (0.65, 1.54) \| 1.43 (1.11, 1.83) \| Q1 \| Reference \| 1.07 (0.67, 1.72) \| 1.57 (1.23, 2.00) \| \| Q2 \| 0.85 (0.66, 1.09) \| 0.90 (0.56, 1.46) \| 1.19 (0.92, 1.54) \| Q2 \| 0.96 (0.75, 1.22) \| 1.24 (0.83, 1.85) \| 1.31 (1.03, 1.67) \| \| Q3 \| 0.91 (0.71, 1.17) \| 1.19 (0.77, 1.84) \| 1.50 (1.16, 1.94) \| Q3 \| 0.91 (0.71, 1.15) \| 0.90 (0.57, 1.42) \| 1.46 (1.13, 1.87) \| \| Q4 \| 1.58 (1.24, 2.01) \| 1.98 (1.32, 2.95) \| 2.43 (1.91, 3.09) \| Q4 \| 1.40 (1.10, 1.78) \| 1.82 (1.22, 2.70) \| 2.15 (1.70, 2.72) \| \| PM_1_ (µg/m^3^) \|  \|  \|  \| O_3_ (µg/m^3^) \|  \|  \|  \| \| Q1 \| Reference \| 1.32 (0.84, 2.08) \| 1.51 (1.18, 1.94) \| Q1 \| Reference \| 1.60 (1.05, 2.46) \| 1.88 (1.45, 2.43) \| \| Q2 \| 0.90 (0.70, 1.16) \| 0.92 (0.60, 1.42) \| 1.28 (1.00, 1.65) \| Q2 \| 1.58 (1.23, 2.02) \| 0.99 (0.62, 1.59) \| 1.84 (1.42, 2.37) \| \| Q3 \| 0.89 (0.69, 1.14) \| 1.22 (0.79, 1.89) \| 1.46 (1.14, 1.87) \| Q3 \| 1.30 (1.01, 1.67) \| 2.10 (1.37, 3.20) \| 2.30 (1.79, 2.97) \| \| Q4 \| 1.84 (1.45, 2.33) \| 1.93 (1.29, 2.90) \| 2.69 (2.12, 3.41) \| Q4 \| 1.75 (1.36, 2.24) \| 2.06 (1.35, 3.14) \| 2.50 (1.95, 3.21) \| \| PM_2.5_ (µg/m^3^) \|  \|  \|  \| PM_10_ (µg/m^3^) \|  \|  \|  \| \| Q1 \| Reference \| 1.20 (0.75, 1.91) \| 1.32 (1.02, 1.70) \| Q1 \| Reference \| 1.15 (0.71, 1.86) \| 1.31 (1.00, 1.71) \| \| Q2 \| 1.02 (0.80, 1.31) \| 1.10 (0.73, 1.64) \| 1.63 (1.28, 2.09) \| Q2 \| 1.09 (0.85, 1.40) \| 1.27 (0.84, 1.92) \| 1.81 (1.42, 2.32) \| \| Q3 \| 0.70 (0.54, 0.90) \| 1.15 (0.73, 1.80) \| 1.21 (0.94, 1.55) \| Q3 \| 1.00 (0.78, 1.29) \| 1.48 (0.95, 2.30) \| 1.58 (1.22, 2.04) \| \| Q4 \| 1.80 (1.42, 2.27) \| 1.69 (1.12, 2.55) \| 2.59 (2.04, 3.28) \| Q4 \| 2.08 (1.63, 2.64) \| 1.98 (1.31, 2.97) \| 2.98 (2.34, 3.79) \| \| SO_2_ (µg/m^3^) \|  \|  \|  \| AQI* \|  \|  \|  \| \| Q1 \| Reference \| 1.03 (0.67, 1.57) \| 1.49 (1.16, 1.91) \| Q1 \| Reference \| 1.46 (0.93, 2.29) \| 1.41 (1.09, 1.83) \| \| Q2 \| 0.69 (0.53, 0.90) \| 0.98 (0.61, 1.58) \| 1.19 (0.91, 1.54) \| Q2 \| 0.92 (0.72, 1.19) \| 0.82 (0.52, 1.28) \| 1.31 (1.01, 1.69) \| \| Q3 \| 1.25 (0.98, 1.59) \| 1.17 (0.73, 1.87) \| 1.92 (1.50, 2.47) \| Q3 \| 0.93 (0.72, 1.19) \| 1.37 (0.90, 2.10) \| 1.66 (1.29, 2.14) \| \| Q4 \| 1.79 (1.40, 2.28) \| 2.19 (1.48, 3.22) \| 2.43 (1.90, 3.10) \| Q4 \| 1.83 (1.44, 2.33) \| 1.88 (1.25, 2.84) \| 2.59 (2.04, 3.30) \|   All model adjusted for age, education, residence, marital status, household expenses per capita, smoke, drink, solid fuel usage, sleep duration, depressive symptoms, non-communicable diseases, installation of residential air cleaner, and residential temperature. ^a^ Based on the WHO Guidelines on Physical Activity and Sedentary Behaviour 2020, inactive was considered as not participating MVPA. And we use the same METs as 300 minutes of moderate physical activity or 150 minutes of vigorous physical activity as the dividing line between insufficiently active and physically active. ^b^ The AQI is a numerical indicator used to assess air quality, based on the concentrations of multiple pollutants, including CO, PM_2.5_, SO_2_, NO_2_, O_3_, and PM_10_. CVD, cardiovascular diseases; MVPA, moderate-vigorous physical activity; CI, confidence interval; CO, Carbon monoxide; PM_1_, particulate matter with aerodynamic diameter ≤1µm; PM_2.5_, particulate matter with aerodynamic diameter ≤2.5µm; SO_2_, Sulfur dioxide; NO_2_, nitrogen dioxide; O_3_, Ozone; PM_10_, particulate matter with aerodynamic diameter ≤10µm; AQI, air quality index. | | |

| Supplemental table 22. Subgroup analysis on the associations between air pollutants and the prevalence of CVD stratified by MVPA categories, CHARLS 2018 (adjusted for additional covariates). | |
| --- | --- |
| \|  \| MVPA level \| \|  \| \| --- \| --- \| --- \| --- \| \| High levels of air pollutants \| Low-medium \| High \| P for interaction \| \| CO \| 1.55 (1.40, 1.70) \| 1.89 (1.53, 2.33) \| 0.087 \| \| PM_1_ \| 1.66 (1.51, 1.83) \| 1.83 (1.48, 2.25) \| 0.414 \| \| PM_2.5_ \| 1.69 (1.53, 1.86) \| 1.89 (1.53, 2.33) \| 0.333 \| \| SO_2_ \| 1.48 (1.34, 1.63) \| 1.91 (1.55, 2.36) \| 0.031 \| \| NO_2_ \| 1.35 (1.23, 1.49) \| 1.55 (1.25, 1.93) \| 0.265 \| \| O_3_ \| 1.20 (1.08, 1.32) \| 1.28 (1.03, 1.59) \| 0.582 \| \| PM_10_ \| 1.65 (1.50, 1.82) \| 1.82 (1.47, 2.25) \| 0.404 \| \| AQI \| 1.59 (1.44, 1.75) \| 1.82 (1.47, 2.25) \| 0.253 \|   P<0.05 was considered statistically significant. The cut off point of air pollutants and moderate-vigorous physical activity are cohort-specific cut-off points at the 75%. Multivariable logistic regression model was used to examine the associations, which were adjusted for sex, age, education, residence, marital status, household expenses per capita, smoke, drink, solid fuel usage, sleep duration, depressive symptoms, non-communicable diseases, installation of residential air cleaner, and residential temperature. The results expressed as odd ratios (OR) and 95% confidence intervals (CI). CVD, cardiovascular diseases; MVPA, moderate-vigorous physical activity; CO, Carbon monoxide; PM_1_, particulate matter with aerodynamic diameter ≤1µm; PM_2.5_, particulate matter with aerodynamic diameter ≤2.5µm; SO_2_, Sulfur dioxide; NO_2_, nitrogen dioxide; O_3_, Ozone; PM_10_, particulate matter with aerodynamic diameter ≤10µm; AQI, air quality index. |  |

| Supplemental table 23. Subgroup analysis on the associations between air pollutants and the prevalence of CVD stratified by MVPA categories in females, CHARLS 2018 (adjusted for additional covariates). | |
| --- | --- |
| \|  \| MVPA level \| \|  \| \| --- \| --- \| --- \| --- \| \| High levels of air pollutants \| Low-medium \| High \| P for interaction \| \| CO \| 1.46 (1.28, 1.66) \| 1.68 (1.24, 2.29) \| 0.399 \| \| PM_1_ \| 1.52 (1.34, 1.73) \| 1.56 (1.15, 2.12) \| 0.869 \| \| PM_2.5_ \| 1.58 (1.39, 1.79) \| 1.66 (1.23, 2.26) \| 0.750 \| \| SO_2_ \| 1.36 (1.19, 1.55) \| 1.70 (1.25, 2.31) \| 0.191 \| \| NO_2_ \| 1.29 (1.13, 1.47) \| 1.38 (1.00, 1.91) \| 0.693 \| \| O_3_ \| 1.12 (0.99, 1.28) \| 1.10 (0.80, 1.52) \| 0.910 \| \| PM_10_ \| 1.53 (1.35, 1.74) \| 1.49 (1.09, 2.05) \| 0.886 \| \| AQI \| 1.47 (1.29, 1.67) \| 1.46 (1.06, 2.00) \| 0.974 \|   P<0.05 was considered statistically significant. The cut off point of air pollutants and moderate-vigorous physical activity are cohort-specific cut-off points at the 75%. Multivariable logistic regression model was used to examine the associations, which were adjusted for age, education, residence, marital status, household expenses per capita, smoke, drink, solid fuel usage, sleep duration, depressive symptoms, non-communicable diseases, installation of residential air cleaner, and residential temperature. The results expressed as odd ratios (OR) and 95% confidence intervals (CI). CVD, cardiovascular diseases; MVPA, moderate-vigorous physical activity; CO, Carbon monoxide; PM_1_, particulate matter with aerodynamic diameter ≤1µm; PM_2.5_, particulate matter with aerodynamic diameter ≤2.5µm; SO_2_, Sulfur dioxide; NO_2_, nitrogen dioxide; O_3_, Ozone; PM_10_, particulate matter with aerodynamic diameter ≤10µm; AQI, air quality index. |  |

| Supplemental table 24. Subgroup analysis on the associations between air pollutants and the prevalence of CVD stratified by MVPA categories in males, CHARLS 2018 (adjusted for additional covariates). | | |
| --- | --- | --- |
| \|  \| MVPA level \| \|  \| \| --- \| --- \| --- \| --- \| \| High levels of air pollutants \| Low-medium \| High \| P for interaction \| \| CO \| 1.67 (1.45, 1.93) \| 2.28 (1.68, 3.08) \| 0.068 \| \| PM_1_ \| 1.81 (1.57, 2.10) \| 2.35 (1.74, 3.18) \| 0.125 \| \| PM_2.5_ \| 1.82 (1.58, 2.11) \| 2.39 (1.77, 3.23) \| 0.109 \| \| SO_2_ \| 1.61 (1.39, 1.87) \| 2.45 (1.81, 3.32) \| 0.013 \| \| NO_2_ \| 1.42 (1.23, 1.64) \| 1.97 (1.44, 2.69) \| 0.059 \| \| O_3_ \| 1.29 (1.11, 1.50) \| 1.57 (1.15, 2.14) \| 0.260 \| \| PM_10_ \| 1.81 (1.56, 2.08) \| 2.44 (1.80, 3.29) \| 0.075 \| \| AQI \| 1.74 (1.51, 2.01) \| 2.46 (1.82, 3.32) \| 0.042 \| \| P<0.05 was considered statistically significant. The cut off point of air pollutants and moderate-vigorous physical activity are cohort-specific cut-off points at the 75%. Multivariable logistic regression model was used to examine the associations, which were adjusted for age, education, residence, marital status, household expenses per capita, smoke, drink, solid fuel usage, sleep duration, depressive symptoms, non-communicable diseases, installation of residential air cleaner, and residential temperature. The results expressed as odd ratios (OR) and 95% confidence intervals (CI). CVD, cardiovascular diseases; MVPA, moderate-vigorous physical activity; CO, Carbon monoxide; PM_1_, particulate matter with aerodynamic diameter ≤1µm; PM_2.5_, particulate matter with aerodynamic diameter ≤2.5µm; SO_2_, Sulfur dioxide; NO_2_, nitrogen dioxide; O_3_, Ozone; PM_10_, particulate matter with aerodynamic diameter ≤10µm; AQI, air quality index. \| \| \| \| | |  |
|  |  |  |
|  |  |  |
|  |  |  |

| \| Supplemental table 25. Dose–response associations of MVPA with the prevalence of CVD stratified by air pollutants concentration, CHARLS 2018 (adjusted for additional covariates). \| \| \| \| \| \| \| --- \| --- \| --- \| --- \| --- \| --- \| \| Air pollutants \| P for overall (low-medium) \| P for nonlinear (low-medium) \| P for overall (high) \| P for nonlinear (high) \| P for interaction \| \| Duration \|  \|  \|  \|  \|  \| \| CO \| <0.001 \| 0.008 \| 0.002 \| 0.040 \| 0.213 \| \| PM_1_ \| <0.001 \| 0.015 \| 0.002 \| 0.055 \| 0.199 \| \| PM_2.5_ \| <0.001 \| 0.019 \| 0.003 \| 0.041 \| 0.118 \| \| SO_2_ \| <0.001 \| 0.034 \| 0.007 \| 0.008 \| 0.002 \| \| NO_2_ \| <0.001 \| 0.012 \| 0.002 \| 0.123 \| 0.130 \| \| O_3_ \| <0.001 \| 0.008 \| 0.002 \| 0.134 \| 0.152 \| \| PM_10_ \| <0.001 \| 0.021 \| 0.002 \| 0.040 \| 0.169 \| \| AQI \| <0.001 \| 0.014 \| 0.007 \| 0.050 \| 0.084 \| \|  \|  \|  \|  \|  \|  \| \| METs \|  \|  \|  \|  \|  \| \| CO \| <0.001 \| 0.004 \| 0.001 \| 0.008 \| 0.096 \| \| PM_1_ \| <0.001 \| 0.003 \| 0.001 \| 0.028 \| 0.169 \| \| PM_2.5_ \| <0.001 \| 0.005 \| 0.002 \| 0.018 \| 0.088 \| \| SO_2_ \| <0.001 \| 0.011 \| 0.004 \| 0.004 \| 0.001 \| \| NO_2_ \| <0.001 \| 0.005 \| 0.000 \| 0.027 \| 0.092 \| \| O_3_ \| <0.001 \| 0.001 \| 0.001 \| 0.098 \| 0.200 \| \| PM_10_ \| <0.001 \| 0.008 \| 0.001 \| 0.008 \| 0.072 \| \| AQI \| <0.001 \| 0.004 \| 0.003 \| 0.016 \| 0.050 \| \| P<0.05 was considered statistically significant. The cut off point of air pollutants and moderate-vigorous physical activity are cohort-specific cut-off points at the 75%. All models were adjusted for was used to examine the associations, which were adjusted for sex, age, education, residence, marital status, household expenses per capita, smoke, drink, solid fuel usage, sleep duration, depressive symptoms, non-communicable diseases, installation of residential air cleaner, and residential temperature. CVD, cardiovascular diseases; MVPA, moderate-vigorous physical activity; METs, Metabolic equivalents; CO, Carbon monoxide; PM_1_, particulate matter with aerodynamic diameter ≤1µm; PM_2.5_, particulate matter with aerodynamic diameter ≤2.5µm; SO_2_, Sulfur dioxide; NO_2_, nitrogen dioxide; O_3_, Ozone; PM_10_, particulate matter with aerodynamic diameter ≤10µm; AQI, air quality index. \| \| \| \| \| \| |
| --- | --- | --- | --- | --- | --- | --- | --- | --- | --- | --- | --- | --- | --- | --- | --- | --- | --- | --- | --- | --- | --- | --- | --- | --- | --- | --- | --- | --- | --- | --- | --- | --- | --- | --- | --- | --- | --- | --- | --- | --- | --- | --- | --- | --- | --- | --- | --- | --- | --- | --- | --- | --- | --- | --- | --- | --- | --- | --- | --- | --- | --- | --- | --- | --- | --- | --- | --- | --- | --- | --- | --- | --- | --- | --- | --- | --- | --- | --- | --- | --- | --- | --- | --- | --- | --- | --- | --- | --- | --- | --- | --- | --- | --- | --- | --- | --- | --- | --- | --- | --- | --- | --- | --- | --- | --- | --- | --- | --- | --- | --- | --- | --- | --- | --- | --- | --- | --- | --- | --- | --- | --- | --- | --- | --- | --- | --- | --- | --- | --- | --- | --- | --- |

| \| Supplemental table 26. Dose–response associations of MVPA with the prevalence of CVD stratified by air pollutants concentration in females, CHARLS 2018 (adjusted for additional covariates). \| \| \| \| \| \| \| --- \| --- \| --- \| --- \| --- \| --- \| \| Air pollutants \| P for overall (low-medium) \| P for nonlinear (low-medium) \| P for overall (high) \| P for nonlinear (high) \| P for interaction \| \| Duration \|  \|  \|  \|  \|  \| \| CO \| <0.001 \| 0.569 \| 0.240 \| 0.371 \| 0.143 \| \| PM_1_ \| <0.001 \| 0.510 \| 0.105 \| 0.321 \| 0.271 \| \| PM_2.5_ \| <0.001 \| 0.583 \| 0.133 \| 0.261 \| 0.171 \| \| SO_2_ \| <0.001 \| 0.414 \| 0.139 \| 0.077 \| 0.009 \| \| NO_2_ \| <0.001 \| 0.093 \| 0.165 \| 0.664 \| 0.145 \| \| O_3_ \| <0.001 \| 0.133 \| 0.087 \| 0.440 \| 0.202 \| \| PM_10_ \| <0.001 \| 0.573 \| 0.069 \| 0.170 \| 0.235 \| \| AQI \| <0.001 \| 0.454 \| 0.184 \| 0.300 \| 0.186 \| \|  \|  \|  \|  \|  \|  \| \| METs \|  \|  \|  \|  \|  \| \| CO \| <0.001 \| 0.332 \| 0.162 \| 0.098 \| 0.034 \| \| PM_1_ \| <0.001 \| 0.328 \| 0.070 \| 0.083 \| 0.118 \| \| PM_2.5_ \| <0.001 \| 0.402 \| 0.084 \| 0.069 \| 0.071 \| \| SO_2_ \| <0.001 \| 0.378 \| 0.030 \| 0.008 \| 0.001 \| \| NO_2_ \| <0.001 \| 0.070 \| 0.073 \| 0.091 \| 0.039 \| \| O_3_ \| <0.001 \| 0.099 \| 0.078 \| 0.203 \| 0.147 \| \| PM_10_ \| <0.001 \| 0.523 \| 0.024 \| 0.015 \| 0.038 \| \| AQI \| <0.001 \| 0.337 \| 0.086 \| 0.052 \| 0.053 \|   P<0.05 was considered statistically significant. The cut off point of air pollutants and moderate-vigorous physical activity are cohort-specific cut-off points at the 75%. All models were adjusted for was used to examine the associations, which were adjusted for age, education, residence, marital status, household expenses per capita, smoke, drink, solid fuel usage, sleep duration, depressive symptoms, non-communicable diseases, installation of residential air cleaner, and residential temperature. CVD, cardiovascular diseases; MVPA, moderate-vigorous physical activity; METs, Metabolic equivalents; CO, Carbon monoxide; PM_1_, particulate matter with aerodynamic diameter ≤1µm; PM_2.5_, particulate matter with aerodynamic diameter ≤2.5µm; SO_2_, Sulfur dioxide; NO_2_, nitrogen dioxide; O_3_, Ozone; PM_10_, particulate matter with aerodynamic diameter ≤10µm; AQI, air quality index. |
| --- | --- | --- | --- | --- | --- | --- | --- | --- | --- | --- | --- | --- | --- | --- | --- | --- | --- | --- | --- | --- | --- | --- | --- | --- | --- | --- | --- | --- | --- | --- | --- | --- | --- | --- | --- | --- | --- | --- | --- | --- | --- | --- | --- | --- | --- | --- | --- | --- | --- | --- | --- | --- | --- | --- | --- | --- | --- | --- | --- | --- | --- | --- | --- | --- | --- | --- | --- | --- | --- | --- | --- | --- | --- | --- | --- | --- | --- | --- | --- | --- | --- | --- | --- | --- | --- | --- | --- | --- | --- | --- | --- | --- | --- | --- | --- | --- | --- | --- | --- | --- | --- | --- | --- | --- | --- | --- | --- | --- | --- | --- | --- | --- | --- | --- | --- | --- | --- | --- | --- | --- | --- | --- | --- | --- | --- | --- |
|  |
|  |

| \| Supplemental table 27. Dose–response associations of MVPA with the prevalence of CVD stratified by air pollutants concentration in males, CHARLS 2018 (adjusted for additional covariates). \| \| \| \| \| \| \| --- \| --- \| --- \| --- \| --- \| --- \| \| Air pollutants \| P for overall (low-medium) \| P for nonlinear (low-medium) \| P for overall (high) \| P for nonlinear (high) \| P for interaction \| \| Duration \|  \|  \|  \|  \|  \| \| CO \| <0.001 \| 0.003 \| 0.007 \| 0.047 \| 0.921 \| \| PM_1_ \| <0.001 \| 0.004 \| 0.027 \| 0.092 \| 0.573 \| \| PM_2.5_ \| <0.001 \| 0.003 \| 0.023 \| 0.094 \| 0.587 \| \| SO_2_ \| <0.001 \| 0.007 \| 0.050 \| 0.037 \| 0.184 \| \| NO_2_ \| <0.001 \| 0.007 \| 0.009 \| 0.090 \| 0.730 \| \| O_3_ \| <0.001 \| 0.003 \| 0.035 \| 0.185 \| 0.728 \| \| PM_10_ \| <0.001 \| 0.002 \| 0.047 \| 0.155 \| 0.497 \| \| AQI \| <0.001 \| 0.003 \| 0.049 \| 0.110 \| 0.458 \| \|  \|  \|  \|  \|  \|  \| \| METs \|  \|  \|  \|  \|  \| \| CO \| <0.001 \| 0.006 \| 0.002 \| 0.034 \| 0.919 \| \| PM_1_ \| <0.001 \| 0.003 \| 0.017 \| 0.126 \| 0.679 \| \| PM_2.5_ \| <0.001 \| 0.003 \| 0.014 \| 0.119 \| 0.686 \| \| SO_2_ \| <0.001 \| 0.006 \| 0.039 \| 0.079 \| 0.390 \| \| NO_2_ \| <0.001 \| 0.007 \| 0.004 \| 0.118 \| 0.854 \| \| O_3_ \| <0.001 \| 0.003 \| 0.014 \| 0.229 \| 0.847 \| \| PM_10_ \| <0.001 \| 0.002 \| 0.033 \| 0.188 \| 0.559 \| \| AQI \| <0.001 \| 0.003 \| 0.034 \| 0.138 \| 0.556 \| \| P<0.05 was considered statistically significant. The cut off point of air pollutants and moderate-vigorous physical activity are cohort-specific cut-off points at the 75%. All models were adjusted for was used to examine the associations, which were adjusted for age, education, residence, marital status, household expenses per capita, smoke, drink, solid fuel usage, sleep duration, depressive symptoms, non-communicable diseases, installation of residential air cleaner, and residential temperature. CVD, cardiovascular diseases; MVPA, moderate-vigorous physical activity; METs, Metabolic equivalents; CO, Carbon monoxide; PM_1_, particulate matter with aerodynamic diameter ≤1µm; PM_2.5_, particulate matter with aerodynamic diameter ≤2.5µm; SO_2_, Sulfur dioxide; NO_2_, nitrogen dioxide; O_3_, Ozone; PM_10_, particulate matter with aerodynamic diameter ≤10µm; AQI, air quality index. \| \| \| \| \| \| |
| --- | --- | --- | --- | --- | --- | --- | --- | --- | --- | --- | --- | --- | --- | --- | --- | --- | --- | --- | --- | --- | --- | --- | --- | --- | --- | --- | --- | --- | --- | --- | --- | --- | --- | --- | --- | --- | --- | --- | --- | --- | --- | --- | --- | --- | --- | --- | --- | --- | --- | --- | --- | --- | --- | --- | --- | --- | --- | --- | --- | --- | --- | --- | --- | --- | --- | --- | --- | --- | --- | --- | --- | --- | --- | --- | --- | --- | --- | --- | --- | --- | --- | --- | --- | --- | --- | --- | --- | --- | --- | --- | --- | --- | --- | --- | --- | --- | --- | --- | --- | --- | --- | --- | --- | --- | --- | --- | --- | --- | --- | --- | --- | --- | --- | --- | --- | --- | --- | --- | --- | --- | --- | --- | --- | --- | --- | --- | --- | --- | --- | --- | --- | --- |

|  |
| --- |
|  |

| Supplemental table 28. Path data of the example in which MVPA is hypothesized as a mediator of the relation between air pollutants and the prevalence of CVD, CHARLS 2018 (adjusted for additional covariates). | | | |
| --- | --- | --- | --- |
| Air pollution | ADE (average) | ACME (average) | Proportion of mediation  (average) |
| CO | 0.056820*** | 0.009150*** | 13.64%*** |
| PM_1_ | 0.001691*** | 0.000271*** | 13.78%*** |
| PM_2.5_ | 0.001261*** | 0.000154*** | 10.83%*** |
| SO_2_ | 0.002621*** | 0.000342*** | 11.54%*** |
| NO_2_ | 0.001237*** | 0.000230*** | 15.56%*** |
| O_3_ | 0.000793*** | 0.000158*** | 16.15%*** |
| PM_10_ | 0.000895*** | 0.000073*** | 7.56%*** |
| AQI | 0.000872*** | 0.000091*** | 9.35%*** |
| *** p<0.001, ** p<0.01, * p<0.05. Multivariable logistic regression model was used to examine the associations, which were adjusted for sex, age, education, residence, marital status, household expenses per capita, smoke, drink, solid fuel usage, sleep duration, depressive symptoms, non-communicable diseases, installation of residential air cleaner, and residential temperature. CVD, cardiovascular diseases; MVPA, moderate-vigorous physical activity; CO, Carbon monoxide; PM_1_, particulate matter with aerodynamic diameter ≤1µm; PM_2.5_, particulate matter with aerodynamic diameter ≤2.5µm; SO_2_, Sulfur dioxide; NO_2_, nitrogen dioxide; O_3_, Ozone; PM_10_, particulate matter with aerodynamic diameter ≤10µm; AQI, air quality index; ACME, average causal mediation effects; ADE, average direct effects. | | | |

| Supplemental table 29. Path data of the example in which MVPA is hypothesized as a mediator of the relation between air pollutants and the prevalence of CVD in females, CHARLS 2018 (adjusted for additional covariates). | | | |
| --- | --- | --- | --- |
| Air pollution | ADE (average) | ACME (average) | Proportion of mediation  (average) |
| CO | 0.020520 | 0.013850*** | 37.38% |
| PM_1_ | 0.000955 | 0.000416*** | 29.15%* |
| PM_2.5_ | 0.001063*** | 0.000230*** | 17.56%*** |
| SO_2_ | 0.001809** | 0.000490*** | 20.94%*** |
| NO_2_ | 0.000653 | 0.000345*** | 32.70% |
| O_3_ | 0.000555 | 0.000290*** | 32.32%* |
| PM_10_ | 0.000895*** | 0.000105*** | 10.50%*** |
| AQI | 0.000820*** | 0.000131*** | 13.40%*** |
| *** p<0.001, ** p<0.01, * p<0.05. Multivariable logistic regression model was used to examine the associations, which were adjusted for age, education, residence, marital status, household expenses per capita, smoke, drink, solid fuel usage, sleep duration, depressive symptoms, non-communicable diseases, installation of residential air cleaner, and residential temperature. CVD, cardiovascular diseases; MVPA, moderate-vigorous physical activity; CO, Carbon monoxide; PM_1_, particulate matter with aerodynamic diameter ≤1µm; PM_2.5_, particulate matter with aerodynamic diameter ≤2.5µm; SO_2_, Sulfur dioxide; NO_2_, nitrogen dioxide; O_3_, Ozone; PM_10_, particulate matter with aerodynamic diameter ≤10µm; AQI, air quality index; ACME, average causal mediation effects; ADE, average direct effects. | | | |

| Supplemental table 30. Path data of the example in which MVPA is hypothesized as a mediator of the relation between air pollutants and the prevalence of CVD in males, CHARLS 2018 (adjusted for additional covariates). | | | |
| --- | --- | --- | --- |
| Air pollution | ADE (average) | ACME (average) | Proportion of mediation  (average) |
| CO | 0.090720*** | 0.005180*** | 5.32%*** |
| PM_1_ | 0.002290*** | 0.000150*** | 5.98%*** |
| PM_2.5_ | 0.001430*** | 0.000090*** | 5.74%*** |
| SO_2_ | 0.003173*** | 0.000218*** | 6.30%*** |
| NO_2_ | 0.001760*** | 0.000134*** | 6.75%*** |
| O_3_ | 0.000797*** | 0.000063*** | 6.92%*** |
| PM_10_ | 0.000877*** | 0.000047*** | 4.97%*** |
| AQI | 0.000883*** | 0.000055*** | 5.82%*** |
| *** p<0.001, ** p<0.01, * p<0.05. Multivariable logistic regression model was used to examine the associations, which were adjusted for age, education, residence, marital status, household expenses per capita, smoke, drink, solid fuel usage, sleep duration, depressive symptoms, non-communicable diseases, installation of residential air cleaner, and residential temperature. CVD, cardiovascular diseases; MVPA, moderate-vigorous physical activity; CO, Carbon monoxide; PM_1_, particulate matter with aerodynamic diameter ≤1µm; PM_2.5_, particulate matter with aerodynamic diameter ≤2.5µm; SO_2_, Sulfur dioxide; NO_2_, nitrogen dioxide; O_3_, Ozone; PM_10_, particulate matter with aerodynamic diameter ≤10µm; AQI, air quality index; ACME, average causal mediation effects; ADE, average direct effects. | | | |

| Supplemental table 31. Joint association of MVPA and air pollutants with the prevalence of CVD, CHARLS 2018 (3-year lag average air pollutants concentrations). |  |
| --- | --- |
| \|  \| Odd Ratio (95%CI) \| \| \|  \| Odd Ratio (95%CI) \| \| \| \| --- \| --- \| --- \| --- \| --- \| --- \| --- \| --- \| \|  \| Physically active^&^ \| Insufficiently active^&^ \| Inactive^&^ \|  \| Physically active^&^ \| Insufficiently active^&^ \| Inactive^&^ \| \| CO (µg/m^3^) \|  \|  \|  \| NO_2_ (µg/m^3^) \|  \|  \|  \| \| Q1 \| Reference \| 1.16 (0.91, 1.48) \| 1.33 (1.14, 1.56) \| Q1 \| Reference \| 1.10 (0.85, 1.42) \| 1.45 (1.24, 1.69) \| \| Q2 \| 0.72 (0.62, 0.84) \| 0.91 (0.69, 1.20) \| 1.23 (1.05, 1.45) \| Q2 \| 0.63 (0.54, 0.74) \| 1.07 (0.83, 1.38) \| 1.04 (0.89, 1.22) \| \| Q3 \| 0.80 (0.69, 0.94) \| 1.14 (0.89, 1.47) \| 1.23 (1.05, 1.45) \| Q3 \| 0.91 (0.79, 1.06) \| 1.00 (0.78, 1.30) \| 1.41 (1.21, 1.64) \| \| Q4 \| 1.45 (1.25, 1.69) \| 1.39 (1.09, 1.78) \| 1.91 (1.65, 2.23) \| Q4 \| 1.31 (1.12, 1.52) \| 1.39 (1.09, 1.76) \| 1.71 (1.48, 1.98) \| \| PM_1_ (µg/m^3^) \|  \|  \|  \| O_3_ (µg/m^3^) \|  \|  \|  \| \| Q1 \| Reference \| 1.37 (1.06, 1.78) \| 1.45 (1.23, 1.72) \| Q1 \| Reference \| 1.47 (1.15, 1.88) \| 1.61 (1.38, 1.89) \| \| Q2 \| 1.07 (0.91, 1.24) \| 1.11 (0.86, 1.42) \| 1.64 (1.40, 1.92) \| Q2 \| 0.86 (0.74, 1.00) \| 0.96 (0.73, 1.25) \| 1.06 (0.89, 1.25) \| \| Q3 \| 0.91 (0.78, 1.07) \| 1.31 (1.01, 1.70) \| 1.47 (1.25, 1.73) \| Q3 \| 1.26 (1.08, 1.46) \| 1.41 (1.10, 1.82) \| 2.10 (1.80, 2.45) \| \| Q4 \| 1.77 (1.52, 2.06) \| 1.86 (1.46, 2.39) \| 2.27 (1.96, 2.64) \| Q4 \| 1.34 (1.14, 1.56) \| 1.48 (1.16, 1.90) \| 1.81 (1.55, 2.10) \| \| PM_2.5_ (µg/m^3^) \|  \|  \|  \| PM_10_ (µg/m^3^) \|  \|  \|  \| \| Q1 \| Reference \| 1.31 (1.01, 1.71) \| 1.37 (1.16, 1.61) \| Q1 \| Reference \| 1.36 (1.04, 1.78) \| 1.37 (1.15, 1.63) \| \| Q2 \| 1.01 (0.87, 1.18) \| 1.09 (0.85, 1.40) \| 1.52 (1.30, 1.78) \| Q2 \| 1.17 (1.00, 1.37) \| 1.46 (1.14, 1.88) \| 1.92 (1.63, 2.25) \| \| Q3 \| 0.89 (0.76, 1.04) \| 1.23 (0.95, 1.59) \| 1.51 (1.29, 1.78) \| Q3 \| 1.39 (1.19, 1.62) \| 1.58 (1.21, 2.05) \| 2.04 (1.73, 2.40) \| \| Q4 \| 1.67 (1.43, 1.95) \| 1.83 (1.44, 2.34) \| 2.19 (1.88, 2.54) \| Q4 \| 2.01 (1.71, 2.36) \| 2.18 (1.71, 2.79) \| 2.68 (2.30, 3.13) \| \| SO_2_ (µg/m^3^) \|  \|  \|  \| AQI* \|  \|  \|  \| \| Q1 \| Reference \| 1.19 (0.93, 1.52) \| 1.44 (1.22, 1.69) \| Q1 \| Reference \| 1.41 (1.09, 1.83) \| 1.45 (1.23, 1.71) \| \| Q2 \| 0.79 (0.67, 0.92) \| 1.14 (0.88, 1.48) \| 1.18 (1.00, 1.40) \| Q2 \| 0.93 (0.79, 1.08) \| 0.89 (0.68, 1.16) \| 1.25 (1.06, 1.48) \| \| Q3 \| 1.11 (0.96, 1.30) \| 1.24 (0.94, 1.64) \| 1.84 (1.57, 2.15) \| Q3 \| 1.16 (0.99, 1.35) \| 1.51 (1.18, 1.95) \| 1.84 (1.57, 2.15) \| \| Q4 \| 1.62 (1.39, 1.90) \| 1.68 (1.32, 2.13) \| 2.06 (1.77, 2.40) \| Q4 \| 1.52 (1.30, 1.77) \| 1.81 (1.42, 2.30) \| 2.25 (1.93, 2.62) \|   All model adjusted for sex, age, education, residence, marital status, household expenses per capita, smoke, drink, solid fuel usage, sleep duration, depressive symptoms, and non-communicable diseases. ^a^ Based on the WHO Guidelines on Physical Activity and Sedentary Behaviour 2020, inactive was considered as not participating MVPA. And we use the same METs as 300 minutes of moderate physical activity or 150 minutes of vigorous physical activity as the dividing line between insufficiently active and physically active. ^b^ The AQI is a numerical indicator used to assess air quality, based on the concentrations of multiple pollutants, including CO, PM_2.5_, SO_2_, NO_2_, O_3_, and PM_10_. CVD, cardiovascular diseases; MVPA, moderate-vigorous physical activity; CI, confidence interval; CO, Carbon monoxide; PM_1_, particulate matter with aerodynamic diameter ≤1µm; PM_2.5_, particulate matter with aerodynamic diameter ≤2.5µm; SO_2_, Sulfur dioxide; NO_2_, nitrogen dioxide; O_3_, Ozone; PM_10_, particulate matter with aerodynamic diameter ≤10µm; AQI, air quality index. | |

| Supplemental table 32. Joint association of MVPA and air pollutants with the prevalence of CVD in females, CHARLS 2018 (3-year lag average air pollutants concentrations). |  |
| --- | --- |
| \|  \| Odd Ratio (95%CI) \| \| \|  \| Odd Ratio (95%CI) \| \| \| \| --- \| --- \| --- \| --- \| --- \| --- \| --- \| --- \| \|  \| Physically active^&^ \| Insufficiently active^&^ \| Inactive^&^ \|  \| Physically active^&^ \| Insufficiently active^&^ \| Inactive^&^ \| \| CO (µg/m^3^) \|  \|  \|  \| NO_2_ (µg/m^3^) \|  \|  \|  \| \| Q1 \| Reference \| 1.11 (0.82, 1.51) \| 1.26 (1.02, 1.56) \| Q1 \| Reference \| 1.10 (0.80, 1.52) \| 1.40 (1.13, 1.73) \| \| Q2 \| 0.69 (0.56, 0.84) \| 0.88 (0.62, 1.25) \| 1.12 (0.89, 1.39) \| Q2 \| 0.61 (0.50, 0.75) \| 0.97 (0.70, 1.35) \| 0.97 (0.77, 1.20) \| \| Q3 \| 0.66 (0.54, 0.81) \| 1.08 (0.79, 1.49) \| 1.08 (0.87, 1.33) \| Q3 \| 0.89 (0.73, 1.09) \| 1.07 (0.78, 1.48) \| 1.39 (1.13, 1.70) \| \| Q4 \| 1.33 (1.09, 1.63) \| 1.05 (0.76, 1.44) \| 1.62 (1.33, 1.98) \| Q4 \| 1.24 (1.01, 1.52) \| 1.13 (0.82, 1.54) \| 1.47 (1.21, 1.79) \| \| PM_1_ (µg/m^3^) \|  \|  \|  \| O_3_ (µg/m^3^) \|  \|  \|  \| \| Q1 \| Reference \| 1.39 (1.00, 1.94) \| 1.48 (1.19, 1.85) \| Q1 \| Reference \| 1.35 (0.99, 1.85) \| 1.51 (1.23, 1.87) \| \| Q2 \| 1.16 (0.95, 1.42) \| 1.13 (0.82, 1.56) \| 1.67 (1.34, 2.07) \| Q2 \| 0.76 (0.62, 0.93) \| 0.84 (0.59, 1.20) \| 0.93 (0.74, 1.18) \| \| Q3 \| 0.96 (0.78, 1.19) \| 1.32 (0.95, 1.83) \| 1.48 (1.19, 1.84) \| Q3 \| 1.12 (0.92, 1.37) \| 1.27 (0.92, 1.76) \| 1.79 (1.46, 2.19) \| \| Q4 \| 1.70 (1.38, 2.10) \| 1.74 (1.27, 2.40) \| 2.07 (1.69, 2.53) \| Q4 \| 1.30 (1.05, 1.60) \| 1.38 (1.01, 1.88) \| 1.67 (1.37, 2.03) \| \| PM_2.5_ (µg/m^3^) \|  \|  \|  \| PM_10_ (µg/m^3^) \|  \|  \|  \| \| Q1 \| Reference \| 1.33 (0.95, 1.85) \| 1.44 (1.15, 1.80) \| Q1 \| Reference \| 1.48 (1.06, 2.08) \| 1.40 (1.11, 1.78) \| \| Q2 \| 1.16 (0.95, 1.42) \| 1.09 (0.78, 1.51) \| 1.62 (1.30, 2.01) \| Q2 \| 1.35 (1.10, 1.67) \| 1.44 (1.04, 1.99) \| 2.00 (1.60, 2.49) \| \| Q3 \| 0.95 (0.77, 1.16) \| 1.38 (1.00, 1.91) \| 1.50 (1.21, 1.87) \| Q3 \| 1.59 (1.30, 1.96) \| 1.81 (1.29, 2.55) \| 2.22 (1.79, 2.76) \| \| Q4 \| 1.69 (1.37, 2.08) \| 1.76 (1.29, 2.42) \| 2.08 (1.70, 2.54) \| Q4 \| 2.00 (1.61, 2.48) \| 2.12 (1.54, 2.91) \| 2.57 (2.09, 3.16) \| \| SO_2_ (µg/m^3^) \|  \|  \|  \| AQI* \|  \|  \|  \| \| Q1 \| Reference \| 1.24 (0.91, 1.70) \| 1.48 (1.18, 1.84) \| Q1 \| Reference \| 1.34 (0.96, 1.87) \| 1.49 (1.20, 1.87) \| \| Q2 \| 0.94 (0.76, 1.15) \| 1.17 (0.84, 1.63) \| 1.19 (0.95, 1.49) \| Q2 \| 0.92 (0.75, 1.14) \| 0.80 (0.56, 1.14) \| 1.32 (1.06, 1.65) \| \| Q3 \| 1.17 (0.95, 1.43) \| 1.46 (1.03, 2.07) \| 1.94 (1.57, 2.39) \| Q3 \| 1.30 (1.06, 1.59) \| 1.70 (1.24, 2.33) \| 1.72 (1.39, 2.12) \| \| Q4 \| 1.64 (1.33, 2.04) \| 1.55 (1.13, 2.12) \| 1.99 (1.63, 2.44) \| Q4 \| 1.41 (1.14, 1.74) \| 1.68 (1.24, 2.30) \| 2.02 (1.65, 2.48) \|   All model adjusted for age, education, residence, marital status, household expenses per capita, smoke, drink, solid fuel usage, sleep duration, depressive symptoms, and non-communicable diseases. ^a^ Based on the WHO Guidelines on Physical Activity and Sedentary Behaviour 2020, inactive was considered as not participating MVPA. And we use the same METs as 300 minutes of moderate physical activity or 150 minutes of vigorous physical activity as the dividing line between insufficiently active and physically active. ^b^ The AQI is a numerical indicator used to assess air quality, based on the concentrations of multiple pollutants, including CO, PM_2.5_, SO_2_, NO_2_, O_3_, and PM_10_. CVD, cardiovascular diseases; MVPA, moderate-vigorous physical activity; CI, confidence interval; CO, Carbon monoxide; PM_1_, particulate matter with aerodynamic diameter ≤1µm; PM_2.5_, particulate matter with aerodynamic diameter ≤2.5µm; SO_2_, Sulfur dioxide; NO_2_, nitrogen dioxide; O_3_, Ozone; PM_10_, particulate matter with aerodynamic diameter ≤10µm; AQI, air quality index. | |

| Supplemental table 33. Joint association of MVPA and air pollutants with the prevalence of CVD in males, CHARLS 2018 (3-year lag average air pollutants concentrations). | |
| --- | --- |
| \|  \| Odd Ratio (95%CI) \| \| \|  \| Odd Ratio (95%CI) \| \| \| \| --- \| --- \| --- \| --- \| --- \| --- \| --- \| --- \| \|  \| Physically active^&^ \| Insufficiently active^&^ \| Inactive^&^ \|  \| Physically active^&^ \| Insufficiently active^&^ \| Inactive^&^ \| \| CO (µg/m^3^) \|  \|  \|  \| NO_2_ (µg/m^3^) \|  \|  \|  \| \| Q1 \| Reference \| 1.25 (0.84, 1.86) \| 1.46 (1.15, 1.85) \| Q1 \| Reference \| 1.08 (0.70, 1.66) \| 1.54 (1.22, 1.95) \| \| Q2 \| 0.79 (0.62, 1.01) \| 0.90 (0.56, 1.44) \| 1.43 (1.11, 1.84) \| Q2 \| 0.70 (0.55, 0.89) \| 1.26 (0.85, 1.88) \| 1.17 (0.92, 1.48) \| \| Q3 \| 1.04 (0.82, 1.32) \| 1.22 (0.81, 1.84) \| 1.45 (1.14, 1.86) \| Q3 \| 0.98 (0.77, 1.23) \| 0.92 (0.61, 1.41) \| 1.46 (1.15, 1.84) \| \| Q4 \| 1.68 (1.33, 2.12) \| 2.17 (1.48, 3.18) \| 2.48 (1.96, 3.13) \| Q4 \| 1.43 (1.14, 1.79) \| 1.90 (1.30, 2.77) \| 2.15 (1.72, 2.70) \| \| PM_1_ (µg/m^3^) \|  \|  \|  \| O_3_ (µg/m^3^) \|  \|  \|  \| \| Q1 \| Reference \| 1.35 (0.87, 2.07) \| 1.43 (1.12, 1.83) \| Q1 \| Reference \| 1.65 (1.10, 2.48) \| 1.79 (1.41, 2.28) \| \| Q2 \| 0.96 (0.76, 1.22) \| 1.13 (0.76, 1.67) \| 1.60 (1.26, 2.02) \| Q2 \| 1.05 (0.82, 1.34) \| 1.20 (0.78, 1.83) \| 1.28 (0.99, 1.65) \| \| Q3 \| 0.88 (0.69, 1.12) \| 1.29 (0.85, 1.96) \| 1.50 (1.18, 1.91) \| Q3 \| 1.50 (1.19, 1.89) \| 1.50 (0.99, 2.28) \| 2.69 (2.12, 3.41) \| \| Q4 \| 1.87 (1.49, 2.35) \| 2.10 (1.43, 3.10) \| 2.64 (2.10, 3.32) \| Q4 \| 1.47 (1.16, 1.87) \| 1.89 (1.28, 2.80) \| 2.11 (1.66, 2.67) \| \| PM_2.5_ (µg/m^3^) \|  \|  \|  \| PM_10_ (µg/m^3^) \|  \|  \|  \| \| Q1 \| Reference \| 1.33 (0.86, 2.07) \| 1.32 (1.03, 1.69) \| Q1 \| Reference \| 1.16 (0.74, 1.84) \| 1.33 (1.03, 1.72) \| \| Q2 \| 0.89 (0.70, 1.14) \| 1.17 (0.79, 1.71) \| 1.51 (1.19, 1.91) \| Q2 \| 0.97 (0.76, 1.25) \| 1.55 (1.05, 2.30) \| 1.83 (1.44, 2.32) \| \| Q3 \| 0.85 (0.67, 1.08) \| 1.05 (0.68, 1.63) \| 1.52 (1.20, 1.93) \| Q3 \| 1.17 (0.92, 1.49) \| 1.34 (0.88, 2.03) \| 1.83 (1.43, 2.34) \| \| Q4 \| 1.73 (1.38, 2.17) \| 2.04 (1.39, 2.99) \| 2.47 (1.97, 3.11) \| Q4 \| 2.02 (1.60, 2.55) \| 2.30 (1.56, 3.39) \| 2.87 (2.27, 3.62) \| \| SO_2_ (µg/m^3^) \|  \|  \|  \| AQI* \|  \|  \|  \| \| Q1 \| Reference \| 1.16 (0.78, 1.72) \| 1.42 (1.12, 1.81) \| Q1 \| Reference \| 1.53 (1.00, 2.33) \| 1.42 (1.10, 1.82) \| \| Q2 \| 0.68 (0.53, 0.88) \| 1.17 (0.78, 1.78) \| 1.26 (0.99, 1.61) \| Q2 \| 0.95 (0.75, 1.21) \| 1.10 (0.74, 1.65) \| 1.26 (0.99, 1.61) \| \| Q3 \| 1.11 (0.88, 1.39) \| 0.99 (0.62, 1.59) \| 1.74 (1.37, 2.21) \| Q3 \| 1.00 (0.79, 1.28) \| 1.24 (0.81, 1.90) \| 1.96 (1.54, 2.48) \| \| Q4 \| 1.64 (1.30, 2.07) \| 2.01 (1.38, 2.91) \| 2.26 (1.80, 2.85) \| Q4 \| 1.71 (1.35, 2.15) \| 2.02 (1.37, 2.98) \| 2.63 (2.09, 3.33) \|   All model adjusted for age, education, residence, marital status, household expenses per capita, smoke, drink, solid fuel usage, sleep duration, depressive symptoms, and non-communicable diseases. ^a^ Based on the WHO Guidelines on Physical Activity and Sedentary Behaviour 2020, inactive was considered as not participating MVPA. And we use the same METs as 300 minutes of moderate physical activity or 150 minutes of vigorous physical activity as the dividing line between insufficiently active and physically active. ^b^ The AQI is a numerical indicator used to assess air quality, based on the concentrations of multiple pollutants, including CO, PM_2.5_, SO_2_, NO_2_, O_3_, and PM_10_. CVD, cardiovascular diseases; MVPA, moderate-vigorous physical activity; CI, confidence interval; CO, Carbon monoxide; PM_1_, particulate matter with aerodynamic diameter ≤1µm; PM_2.5_, particulate matter with aerodynamic diameter ≤2.5µm; SO_2_, Sulfur dioxide; NO_2_, nitrogen dioxide; O_3_, Ozone; PM_10_, particulate matter with aerodynamic diameter ≤10µm; AQI, air quality index. |  |

| Supplemental table 34. Subgroup analysis on the associations between air pollutants and the prevalence of CVD stratified by MVPA categories, CHARLS 2018 (3-year lag average air pollutants concentrations). |  |
| --- | --- |
| \|  \| MVPA level \| \|  \| \| --- \| --- \| --- \| --- \| \| High levels of air pollutants \| Low-medium \| High \| P for interaction \| \| CO \| 1.55 (1.41, 1.70) \| 1.93 (1.58, 2.36) \| 0.044 \| \| PM_1_ \| 1.58 (1.45, 1.74) \| 1.86 (1.52, 2.28) \| 0.160 \| \| PM_2.5_ \| 1.59 (1.45, 1.74) \| 1.77 (1.44, 2.18) \| 0.338 \| \| SO_2_ \| 1.48 (1.35, 1.62) \| 1.78 (1.45, 2.20) \| 0.105 \| \| NO_2_ \| 1.39 (1.27, 1.53) \| 1.63 (1.32, 2.01) \| 0.176 \| \| O_3_ \| 1.17 (1.06, 1.28) \| 1.20 (0.97, 1.49) \| 0.803 \| \| PM_10_ \| 1.56 (1.43, 1.71) \| 1.75 (1.42, 2.15) \| 0.324 \| \| AQI \| 1.55 (1.41, 1.70) \| 1.57 (1.28, 1.94) \| 0.881 \|   P<0.05 was considered statistically significant. The cut off point of air pollutants and moderate-vigorous physical activity are cohort-specific cut-off points at the 75%. Multivariable logistic regression model was used to examine the associations, which were adjusted for sex, age, education, residence, marital status, household expenses per capita, smoke, drink, solid fuel usage, sleep duration, depressive symptoms, and non-communicable diseases. The results expressed as odd ratios (OR) and 95% confidence intervals (CI). CVD, cardiovascular diseases; MVPA, moderate-vigorous physical activity; CO, Carbon monoxide; PM_1_, particulate matter with aerodynamic diameter ≤1µm; PM_2.5_, particulate matter with aerodynamic diameter ≤2.5µm; SO_2_, Sulfur dioxide; NO_2_, nitrogen dioxide; O_3_, Ozone; PM_10_, particulate matter with aerodynamic diameter ≤10µm; AQI, air quality index. | |

| Supplemental table 35. Subgroup analysis on the associations between air pollutants and the prevalence of CVD stratified by MVPA categories in females, CHARLS 2018 (3-year lag average air pollutants concentrations). |  |
| --- | --- |
| \|  \| MVPA level \| \|  \| \| --- \| --- \| --- \| --- \| \| High levels of air pollutants \| Low-medium \| High \| P for interaction \| \| CO \| 1.46 (1.29, 1.66) \| 1.77 (1.33, 2.37) \| 0.229 \| \| PM_1_ \| 1.46 (1.29, 1.65) \| 1.70 (1.27, 2.29) \| 0.342 \| \| PM_2.5_ \| 1.48 (1.31, 1.67) \| 1.53 (1.13, 2.08) \| 0.847 \| \| SO_2_ \| 1.40 (1.23, 1.58) \| 1.57 (1.16, 2.14) \| 0.470 \| \| NO_2_ \| 1.27 (1.13, 1.44) \| 1.52 (1.12, 2.08) \| 0.295 \| \| O_3_ \| 1.20 (1.06, 1.37) \| 1.12 (0.81, 1.54) \| 0.674 \| \| PM_10_ \| 1.44 (1.27, 1.63) \| 1.49 (1.09, 2.03) \| 0.834 \| \| AQI \| 1.44 (1.27, 1.63) \| 1.25 (0.91, 1.71) \| 0.405 \|   P<0.05 was considered statistically significant. The cut off point of air pollutants and moderate-vigorous physical activity are cohort-specific cut-off points at the 75%. Multivariable logistic regression model was used to examine the associations, which were adjusted for age, education, residence, marital status, household expenses per capita, smoke, drink, solid fuel usage, sleep duration, depressive symptoms, and non-communicable diseases. The results expressed as odd ratios (OR) and 95% confidence intervals (CI). CVD, cardiovascular diseases; MVPA, moderate-vigorous physical activity; CO, Carbon monoxide; PM_1_, particulate matter with aerodynamic diameter ≤1µm; PM_2.5_, particulate matter with aerodynamic diameter ≤2.5µm; SO_2_, Sulfur dioxide; NO_2_, nitrogen dioxide; O_3_, Ozone; PM_10_, particulate matter with aerodynamic diameter ≤10µm; AQI, air quality index. | |

| Supplemental table 36. Subgroup analysis on the associations between air pollutants and the prevalence of CVD stratified by MVPA categories in males, CHARLS 2018 (3-year lag average air pollutants concentrations). |  |
| --- | --- |
| \|  \| MVPA level \| \|  \| \| --- \| --- \| --- \| --- \| \| High levels of air pollutants \| Low-medium \| High \| P for interaction \| \| CO \| 1.67 (1.45, 1.92) \| 2.27 (1.71, 3.02) \| 0.054 \| \| PM_1_ \| 1.79 (1.56, 2.06) \| 2.30 (1.72, 3.07) \| 0.124 \| \| PM_2.5_ \| 1.72 (1.50, 1.97) \| 2.28 (1.70, 3.04) \| 0.084 \| \| SO_2_ \| 1.56 (1.36, 1.79) \| 2.28 (1.70, 3.06) \| 0.022 \| \| NO_2_ \| 1.54 (1.34, 1.77) \| 1.95 (1.45, 2.62) \| 0.158 \| \| O_3_ \| 1.09 (0.94, 1.26) \| 1.43 (1.05, 1.94) \| 0.114 \| \| PM_10_ \| 1.76 (1.53, 2.02) \| 2.29 (1.71, 3.07) \| 0.103 \| \| AQI \| 1.67 (1.46, 1.93) \| 2.21 (1.64, 2.96) \| 0.094 \|   P<0.05 was considered statistically significant. The cut off point of air pollutants and moderate-vigorous physical activity are cohort-specific cut-off points at the 75%. Multivariable logistic regression model was used to examine the associations, which were adjusted for age, education, residence, marital status, household expenses per capita, smoke, drink, solid fuel usage, sleep duration, depressive symptoms, and non-communicable diseases. The results expressed as odd ratios (OR) and 95% confidence intervals (CI). CVD, cardiovascular diseases; MVPA, moderate-vigorous physical activity; CO, Carbon monoxide; PM_1_, particulate matter with aerodynamic diameter ≤1µm; PM_2.5_, particulate matter with aerodynamic diameter ≤2.5µm; SO_2_, Sulfur dioxide; NO_2_, nitrogen dioxide; O_3_, Ozone; PM_10_, particulate matter with aerodynamic diameter ≤10µm; AQI, air quality index. | |

| Supplemental table 37. Dose–response associations of MVPA with the prevalence of CVD stratified by air pollutants concentration, CHARLS 2018 (3-year lag average air pollutants concentrations). |
| --- |
| \| Air pollutants \| P for overall (low-medium) \| P for nonlinear (low-medium) \| P for overall (high) \| P for nonlinear (high) \| P for interaction \| \| --- \| --- \| --- \| --- \| --- \| --- \| \| Duration \|  \|  \|  \|  \|  \| \| CO \| <0.001 \| 0.022 \| 0.009 \| 0.026 \| 0.017 \| \| PM_1_ \| <0.001 \| 0.018 \| 0.009 \| 0.080 \| 0.024 \| \| PM_2.5_ \| <0.001 \| 0.018 \| 0.007 \| 0.092 \| 0.071 \| \| SO_2_ \| <0.001 \| 0.024 \| 0.016 \| 0.036 \| 0.005 \| \| NO_2_ \| <0.001 \| 0.015 \| 0.004 \| 0.129 \| 0.071 \| \| O_3_ \| <0.001 \| 0.007 \| 0.010 \| 0.246 \| 0.149 \| \| PM_10_ \| <0.001 \| 0.019 \| 0.005 \| 0.077 \| 0.085 \| \| AQI \| <0.001 \| 0.024 \| 0.000 \| 0.045 \| 0.402 \| \|  \|  \|  \|  \|  \|  \| \| METs \|  \|  \|  \|  \|  \| \| CO \| <0.001 \| 0.005 \| 0.004 \| 0.009 \| 0.010 \| \| PM_1_ \| <0.001 \| 0.004 \| 0.004 \| 0.030 \| 0.019 \| \| PM_2.5_ \| <0.001 \| 0.007 \| 0.002 \| 0.013 \| 0.025 \| \| SO_2_ \| <0.001 \| 0.004 \| 0.010 \| 0.025 \| 0.006 \| \| NO_2_ \| <0.001 \| 0.006 \| 0.001 \| 0.015 \| 0.022 \| \| O_3_ \| <0.001 \| 0.001 \| 0.005 \| 0.135 \| 0.172 \| \| PM_10_ \| <0.001 \| 0.005 \| 0.002 \| 0.016 \| 0.046 \| \| AQI \| <0.001 \| 0.007 \| 0.000 \| 0.008 \| 0.284 \| |
| \| P<0.05 was considered statistically significant. The cut off point of air pollutants and moderate-vigorous physical activity are cohort-specific cut-off points at the 75%. All models were adjusted for was used to examine the associations, which were adjusted for sex, age, education, residence, marital status, household expenses per capita, smoke, drink, solid fuel usage, sleep duration, depressive symptoms, and non-communicable diseases. CVD, cardiovascular diseases; MVPA, moderate-vigorous physical activity; METs, Metabolic equivalents; CO, Carbon monoxide; PM_1_, particulate matter with aerodynamic diameter ≤1µm; PM_2.5_, particulate matter with aerodynamic diameter ≤2.5µm; SO_2_, Sulfur dioxide; NO_2_, nitrogen dioxide; O_3_, Ozone; PM_10_, particulate matter with aerodynamic diameter ≤10µm; AQI, air quality index. \| \| --- \| |
|  |

|  |
| --- |
| Supplemental table 38. Dose–response associations of MVPA with the prevalence of CVD stratified by air pollutants concentration in females, CHARLS 2018 (3-year lag average air pollutants concentrations). |
| \| Air pollutants \| P for overall (low-medium) \| P for nonlinear (low-medium) \| P for overall (high) \| P for nonlinear (high) \| P for interaction \| \| --- \| --- \| --- \| --- \| --- \| --- \| \| Duration \|  \|  \|  \|  \|  \| \| CO \| <0.001 \| 0.477 \| 0.346 \| 0.210 \| 0.021 \| \| PM_1_ \| <0.001 \| 0.406 \| 0.365 \| 0.427 \| 0.072 \| \| PM_2.5_ \| <0.001 \| 0.375 \| 0.313 \| 0.640 \| 0.253 \| \| SO_2_ \| <0.001 \| 0.345 \| 0.256 \| 0.177 \| 0.031 \| \| NO_2_ \| <0.001 \| 0.238 \| 0.395 \| 0.596 \| 0.085 \| \| O_3_ \| <0.001 \| 0.347 \| 0.086 \| 0.406 \| 0.381 \| \| PM_10_ \| <0.001 \| 0.470 \| 0.160 \| 0.267 \| 0.173 \| \| AQI \| <0.001 \| 0.500 \| 0.024 \| 0.357 \| 0.619 \| \|  \|  \|  \|  \|  \|  \| \| METs \|  \|  \|  \|  \|  \| \| CO \| <0.001 \| 0.256 \| 0.158 \| 0.057 \| 0.005 \| \| PM_1_ \| <0.001 \| 0.229 \| 0.237 \| 0.139 \| 0.029 \| \| PM_2.5_ \| <0.001 \| 0.263 \| 0.173 \| 0.109 \| 0.060 \| \| SO_2_ \| <0.001 \| 0.205 \| 0.113 \| 0.042 \| 0.008 \| \| NO_2_ \| <0.001 \| 0.143 \| 0.207 \| 0.109 \| 0.017 \| \| O_3_ \| <0.001 \| 0.217 \| 0.080 \| 0.122 \| 0.164 \| \| PM_10_ \| <0.001 \| 0.348 \| 0.069 \| 0.039 \| 0.038 \| \| AQI \| <0.001 \| 0.394 \| 0.013 \| 0.029 \| 0.161 \| |
| \| P<0.05 was considered statistically significant. The cut off point of air pollutants and moderate-vigorous physical activity are cohort-specific cut-off points at the 75%. All models were adjusted for was used to examine the associations, which were adjusted for age, education, residence, marital status, household expenses per capita, smoke, drink, solid fuel usage, sleep duration, depressive symptoms, and non-communicable diseases. CVD, cardiovascular diseases; MVPA, moderate-vigorous physical activity; METs, Metabolic equivalents; CO, Carbon monoxide; PM_1_, particulate matter with aerodynamic diameter ≤1µm; PM_2.5_, particulate matter with aerodynamic diameter ≤2.5µm; SO_2_, Sulfur dioxide; NO_2_, nitrogen dioxide; O_3_, Ozone; PM_10_, particulate matter with aerodynamic diameter ≤10µm; AQI, air quality index. \| \| --- \| |

| Supplemental table 39. Dose–response associations of MVPA with the prevalence of CVD stratified by air pollutants concentration in males, CHARLS 2018 (3-year lag average air pollutants concentrations).   \| Air pollutants \| P for overall (low-medium) \| P for nonlinear (low-medium) \| P for overall (high) \| P for nonlinear (high) \| P for interaction \| \| --- \| --- \| --- \| --- \| --- \| --- \| \| Duration \|  \|  \|  \|  \|  \| \| CO \| <0.001 \| 0.006 \| 0.020 \| 0.074 \| 0.455 \| \| PM_1_ \| <0.001 \| 0.005 \| 0.022 \| 0.104 \| 0.440 \| \| PM_2.5_ \| <0.001 \| 0.005 \| 0.020 \| 0.113 \| 0.405 \| \| SO_2_ \| <0.001 \| 0.008 \| 0.055 \| 0.090 \| 0.173 \| \| NO_2_ \| <0.001 \| 0.010 \| 0.006 \| 0.117 \| 0.694 \| \| O_3_ \| <0.001 \| 0.002 \| 0.134 \| 0.438 \| 0.228 \| \| PM_10_ \| <0.001 \| 0.003 \| 0.035 \| 0.189 \| 0.462 \| \| AQI \| <0.001 \| 0.006 \| 0.015 \| 0.101 \| 0.571 \| \|  \|  \|  \|  \|  \|  \| \| METs \|  \|  \|  \|  \|  \| \| CO \| <0.001 \| 0.004 \| 0.011 \| 0.073 \| 0.571 \| \| PM_1_ \| <0.001 \| 0.003 \| 0.013 \| 0.110 \| 0.539 \| \| PM_2.5_ \| <0.001 \| 0.004 \| 0.010 \| 0.070 \| 0.438 \| \| SO_2_ \| <0.001 \| 0.003 \| 0.035 \| 0.157 \| 0.330 \| \| NO_2_ \| <0.001 \| 0.009 \| 0.003 \| 0.058 \| 0.645 \| \| O_3_ \| <0.001 \| 0.001 \| 0.051 \| 0.539 \| 0.352 \| \| PM_10_ \| <0.001 \| 0.002 \| 0.023 \| 0.193 \| 0.525 \| \| AQI \| <0.001 \| 0.003 \| 0.006 \| 0.131 \| 0.750 \| |
| --- | --- | --- | --- | --- | --- | --- | --- | --- | --- | --- | --- | --- | --- | --- | --- | --- | --- | --- | --- | --- | --- | --- | --- | --- | --- | --- | --- | --- | --- | --- | --- | --- | --- | --- | --- | --- | --- | --- | --- | --- | --- | --- | --- | --- | --- | --- | --- | --- | --- | --- | --- | --- | --- | --- | --- | --- | --- | --- | --- | --- | --- | --- | --- | --- | --- | --- | --- | --- | --- | --- | --- | --- | --- | --- | --- | --- | --- | --- | --- | --- | --- | --- | --- | --- | --- | --- | --- | --- | --- | --- | --- | --- | --- | --- | --- | --- | --- | --- | --- | --- | --- | --- | --- | --- | --- | --- | --- | --- | --- | --- | --- | --- | --- | --- | --- | --- | --- | --- | --- | --- |
| P<0.05 was considered statistically significant. The cut off point of air pollutants and moderate-vigorous physical activity are cohort-specific cut-off points at the 75%. All models were adjusted for was used to examine the associations, which were adjusted for age, education, residence, marital status, household expenses per capita, smoke, drink, solid fuel usage, sleep duration, depressive symptoms, and non-communicable diseases. CVD, cardiovascular diseases; MVPA, moderate-vigorous physical activity; METs, Metabolic equivalents; CO, Carbon monoxide; PM_1_, particulate matter with aerodynamic diameter ≤1µm; PM_2.5_, particulate matter with aerodynamic diameter ≤2.5µm; SO_2_, Sulfur dioxide; NO_2_, nitrogen dioxide; O_3_, Ozone; PM_10_, particulate matter with aerodynamic diameter ≤10µm; AQI, air quality index. |

| Supplemental table 40. Path data of the example in which MVPA is hypothesized as a mediator of the relation between air pollutants and the prevalence of CVD, CHARLS 2018 (3-year lag average air pollutants concentrations). | | | |  |
| --- | --- | --- | --- | --- |
| Air pollution | ADE (average) | ACME (average) | Proportion of mediation  (average) |  |
| CO | 0.062920*** | 0.008760*** | 12.15%*** |  |
| PM_1_ | 0.001930*** | 0.000256*** | 11.64%*** |  |
| PM_2.5_ | 0.001328*** | 0.000142*** | 9.66%*** |  |
| SO_2_ | 0.002364*** | 0.000260*** | 9.91%*** |  |
| NO_2_ | 0.001740*** | 0.000246*** | 12.30%*** |  |
| O_3_ | 0.000830** | 0.000168*** | 16.27%*** |  |
| PM_10_ | 0.000860*** | 0.000070*** | 7.49%*** |  |
| AQI | 0.000900*** | 0.000088*** | 8.84%*** |  |
| *** p<0.001, ** p<0.01, * p<0.05. Multivariable logistic regression model was used to examine the associations, which were adjusted for sex, age, education, residence, marital status, household expenses per capita, smoke, drink, solid fuel usage, sleep duration, depressive symptoms, and non-communicable diseases. CVD, cardiovascular diseases; MVPA, moderate-vigorous physical activity; CO, Carbon monoxide; PM_1_, particulate matter with aerodynamic diameter ≤1µm; PM_2.5_, particulate matter with aerodynamic diameter ≤2.5µm; SO_2_, Sulfur dioxide; NO_2_, nitrogen dioxide; O_3_, Ozone; PM_10_, particulate matter with aerodynamic diameter ≤10µm; AQI, air quality index; ACME, average causal mediation effects; ADE, average direct effects. | | | | |

| Supplemental table 41. Path data of the example in which MVPA is hypothesized as a mediator of the relation between air pollutants and the prevalence of CVD in females, CHARLS 2018 (3-year lag average air pollutants concentrations). | | | | |
| --- | --- | --- | --- | --- |
| Air pollution | ADE (average) | ACME (average) | Proportion of mediation  (average) | |
| CO | 0.039900** | 0.012370*** | 23.30%*** | |
| PM_1_ | 0.001497*** | 0.000375*** | 19.85%*** | |
| PM_2.5_ | 0.001230*** | 0.000199*** | 13.89%*** | |
| SO_2_ | 0.001946*** | 0.000358*** | 15.19%*** | |
| NO_2_ | 0.001440** | 0.000343*** | 19.10%*** | |
| O_3_ | 0.000627 | 0.000293*** | 29.42%* | |
| PM_10_ | 0.000883*** | 0.000094*** | 9.56%*** | |
| AQI | 0.000884*** | 0.000123*** | 12.10%*** | |
| *** p<0.001, ** p<0.01, * p<0.05. Multivariable logistic regression model was used to examine the associations, which were adjusted for age, education, residence, marital status, household expenses per capita, smoke, drink, solid fuel usage, sleep duration, depressive symptoms, and non-communicable diseases. CVD, cardiovascular diseases; MVPA, moderate-vigorous physical activity; CO, Carbon monoxide; PM_1_, particulate matter with aerodynamic diameter ≤1µm; PM_2.5_, particulate matter with aerodynamic diameter ≤2.5µm; SO_2_, Sulfur dioxide; NO_2_, nitrogen dioxide; O_3_, Ozone; PM_10_, particulate matter with aerodynamic diameter ≤10µm; AQI, air quality index; ACME, average causal mediation effects; ADE, average direct effects. | | | |  |

| Supplemental table 42. Path data of the example in which MVPA is hypothesized as a mediator of the relation between air pollutants and the prevalence of CVD in males, CHARLS 2018 (3-year lag average air pollutants concentrations). | | | | |
| --- | --- | --- | --- | --- |
| Air pollution | ADE (average) | ACME (average) | Proportion of mediation  (average) | |
| CO | 0.084430*** | 0.005580*** | 6.16%*** | |
| PM_1_ | 0.002290*** | 0.000152*** | 6.14%*** | |
| PM_2.5_ | 0.001390*** | 0.000090*** | 6.01%*** | |
| SO_2_ | 0.002615*** | 0.000176*** | 6.28%*** | |
| NO_2_ | 0.001990*** | 0.000159*** | 7.24%*** | |
| O_3_ | 0.000851*** | 0.000071*** | 7.16%*** | |
| PM_10_ | 0.000828*** | 0.000047*** | 5.29%*** | |
| AQI | 0.000888*** | 0.000054*** | 5.71%*** | |
| *** p<0.001, ** p<0.01, * p<0.05. Multivariable logistic regression model was used to examine the associations, which were adjusted for age, education, residence, marital status, household expenses per capita, smoke, drink, solid fuel usage, sleep duration, depressive symptoms, and non-communicable diseases. CVD, cardiovascular diseases; MVPA, moderate-vigorous physical activity; CO, Carbon monoxide; PM_1_, particulate matter with aerodynamic diameter ≤1µm; PM_2.5_, particulate matter with aerodynamic diameter ≤2.5µm; SO_2_, Sulfur dioxide; NO_2_, nitrogen dioxide; O_3_, Ozone; PM_10_, particulate matter with aerodynamic diameter ≤10µm; AQI, air quality index; ACME, average causal mediation effects; ADE, average direct effects. | | | |  |

| Supplemental table 43. Joint association of MVPA and air pollutants with the prevalence of CVD, CHARLS 2018 (Individual weights were further adjusted). |  |
| --- | --- |
| \|  \| Odd Ratio (95%CI) \| \| \|  \| Odd Ratio (95%CI) \| \| \| \| --- \| --- \| --- \| --- \| --- \| --- \| --- \| --- \| \|  \| Physically active^&^ \| Insufficiently active^&^ \| Inactive^&^ \|  \| Physically active^&^ \| Insufficiently active^&^ \| Inactive^&^ \| \| CO (µg/m^3^) \|  \|  \|  \| NO_2_ (µg/m^3^) \|  \|  \|  \| \| Q1 \| Reference \| 1.05 (0.80, 1.38) \| 1.43 (1.19, 1.71) \| Q1 \| Reference \| 0.88 (0.66, 1.18) \| 1.43 (1.20, 1.71) \| \| Q2 \| 0.78 (0.65, 0.93) \| 0.87 (0.63, 1.21) \| 1.08 (0.90, 1.30) \| Q2 \| 0.81 (0.69, 0.97) \| 1.17 (0.89, 1.53) \| 1.25 (1.05, 1.49) \| \| Q3 \| 0.83 (0.69, 0.98) \| 1.20 (0.91, 1.58) \| 1.34 (1.11, 1.61) \| Q3 \| 0.84 (0.71, 0.99) \| 0.97 (0.72, 1.30) \| 1.31 (1.10, 1.57) \| \| Q4 \| 1.50 (1.26, 1.78) \| 1.43 (1.08, 1.89) \| 2.03 (1.72, 2.40) \| Q4 \| 1.25 (1.05, 1.48) \| 1.46 (1.11, 1.90) \| 1.70 (1.44, 2.00) \| \| PM_1_ (µg/m^3^) \|  \|  \|  \| O_3_ (µg/m^3^) \|  \|  \|  \| \| Q1 \| Reference \| 1.26 (0.94, 1.68) \| 1.39 (1.16, 1.68) \| Q1 \| Reference \| 1.33 (0.99, 1.77) \| 1.58 (1.31, 1.89) \| \| Q2 \| 1.00 (0.84, 1.19) \| 0.90 (0.68, 1.20) \| 1.49 (1.24, 1.78) \| Q2 \| 1.26 (1.06, 1.50) \| 1.26 (0.95, 1.66) \| 1.62 (1.35, 1.94) \| \| Q3 \| 0.93 (0.78, 1.11) \| 1.48 (1.12, 1.97) \| 1.47 (1.23, 1.77) \| Q3 \| 1.17 (0.98, 1.39) \| 1.43 (1.08, 1.90) \| 2.04 (1.71, 2.43) \| \| Q4 \| 1.81 (1.53, 2.15) \| 1.78 (1.34, 2.36) \| 2.36 (1.99, 2.78) \| Q4 \| 1.44 (1.21, 1.72) \| 1.56 (1.17, 2.09) \| 1.88 (1.58, 2.24) \| \| PM_2.5_ (µg/m^3^) \|  \|  \|  \| PM_10_ (µg/m^3^) \|  \|  \|  \| \| Q1 \| Reference \| 1.12 (0.83, 1.51) \| 1.34 (1.11, 1.62) \| Q1 \| Reference \| 1.19 (0.88, 1.61) \| 1.41 (1.15, 1.72) \| \| Q2 \| 1.25 (1.05, 1.48) \| 1.26 (0.97, 1.65) \| 1.90 (1.59, 2.27) \| Q2 \| 1.31 (1.10, 1.56) \| 1.49 (1.13, 1.98) \| 1.98 (1.64, 2.37) \| \| Q3 \| 0.77 (0.64, 0.92) \| 1.21 (0.90, 1.63) \| 1.25 (1.04, 1.50) \| Q3 \| 1.33 (1.11, 1.58) \| 1.66 (1.24, 2.22) \| 1.91 (1.59, 2.31) \| \| Q4 \| 1.90 (1.60, 2.25) \| 1.88 (1.42, 2.49) \| 2.42 (2.04, 2.87) \| Q4 \| 2.18 (1.82, 2.60) \| 2.17 (1.64, 2.87) \| 2.92 (2.46, 3.47) \| \| SO_2_ (µg/m^3^) \|  \|  \|  \| AQI* \|  \|  \|  \| \| Q1 \| Reference \| 1.19 (0.91, 1.55) \| 1.45 (1.21, 1.74) \| Q1 \| Reference \| 1.14 (0.86, 1.53) \| 1.47 (1.22, 1.76) \| \| Q2 \| 0.73 (0.60, 0.87) \| 0.93 (0.68, 1.27) \| 1.15 (0.95, 1.39) \| Q2 \| 0.84 (0.70, 0.99) \| 0.76 (0.56, 1.04) \| 1.20 (1.00, 1.45) \| \| Q3 \| 1.33 (1.13, 1.58) \| 1.43 (1.06, 1.92) \| 2.05 (1.72, 2.45) \| Q3 \| 1.02 (0.86, 1.21) \| 1.56 (1.18, 2.05) \| 1.59 (1.33, 1.91) \| \| Q4 \| 1.68 (1.40, 2.00) \| 1.69 (1.29, 2.21) \| 2.09 (1.76, 2.48) \| Q4 \| 1.70 (1.43, 2.02) \| 1.68 (1.27, 2.21) \| 2.18 (1.84, 2.58) \|   All model adjusted for sex, age, education, residence, marital status, household expenses per capita, smoke, drink, solid fuel usage, sleep duration, depressive symptoms, and non-communicable diseases. ^a^ Based on the WHO Guidelines on Physical Activity and Sedentary Behaviour 2020, inactive was considered as not participating MVPA. And we use the same METs as 300 minutes of moderate physical activity or 150 minutes of vigorous physical activity as the dividing line between insufficiently active and physically active. ^b^ The AQI is a numerical indicator used to assess air quality, based on the concentrations of multiple pollutants, including CO, PM_2.5_, SO_2_, NO_2_, O_3_, and PM_10_. CVD, cardiovascular diseases; MVPA, moderate-vigorous physical activity; CI, confidence interval; CO, Carbon monoxide; PM_1_, particulate matter with aerodynamic diameter ≤1µm; PM_2.5_, particulate matter with aerodynamic diameter ≤2.5µm; SO_2_, Sulfur dioxide; NO_2_, nitrogen dioxide; O_3_, Ozone; PM_10_, particulate matter with aerodynamic diameter ≤10µm; AQI, air quality index. | |

| Supplemental table 44. Joint association of MVPA and air pollutants with the prevalence of CVD in females, CHARLS 2018 (Individual weights were further adjusted). |  |
| --- | --- |
| \|  \| Odd Ratio (95%CI) \| \| \|  \| Odd Ratio (95%CI) \| \| \| \| --- \| --- \| --- \| --- \| --- \| --- \| --- \| --- \| \|  \| Physically active^&^ \| Insufficiently active^&^ \| Inactive^&^ \|  \| Physically active^&^ \| Insufficiently active^&^ \| Inactive^&^ \| \| CO (µg/m^3^) \|  \|  \|  \| NO_2_ (µg/m^3^) \|  \|  \|  \| \| Q1 \| Reference \| 1.01 (0.72, 1.41) \| 1.37 (1.08, 1.74) \| Q1 \| Reference \| 0.89 (0.62, 1.27) \| 1.40 (1.11, 1.78) \| \| Q2 \| 0.75 (0.60, 0.95) \| 0.86 (0.56, 1.32) \| 1.08 (0.84, 1.37) \| Q2 \| 0.76 (0.61, 0.95) \| 1.19 (0.85, 1.66) \| 1.18 (0.92, 1.50) \| \| Q3 \| 0.76 (0.60, 0.96) \| 1.37 (0.98, 1.93) \| 1.19 (0.93, 1.52) \| Q3 \| 0.77 (0.62, 0.96) \| 1.00 (0.68, 1.47) \| 1.25 (0.99, 1.58) \| \| Q4 \| 1.51 (1.20, 1.90) \| 1.10 (0.77, 1.57) \| 1.87 (1.49, 2.33) \| Q4 \| 1.28 (1.02, 1.62) \| 1.18 (0.83, 1.68) \| 1.52 (1.22, 1.90) \| \| PM_1_ (µg/m^3^) \|  \|  \|  \| O_3_ (µg/m^3^) \|  \|  \|  \| \| Q1 \| Reference \| 1.30 (0.90, 1.86) \| 1.43 (1.11, 1.83) \| Q1 \| Reference \| 1.38 (0.97, 1.98) \| 1.43 (1.12, 1.83) \| \| Q2 \| 1.05 (0.84, 1.32) \| 0.91 (0.63, 1.30) \| 1.54 (1.20, 1.97) \| Q2 \| 1.12 (0.89, 1.40) \| 1.34 (0.96, 1.88) \| 1.55 (1.21, 1.98) \| \| Q3 \| 0.98 (0.78, 1.24) \| 1.60 (1.13, 2.27) \| 1.43 (1.12, 1.83) \| Q3 \| 1.13 (0.90, 1.42) \| 1.07 (0.74, 1.56) \| 2.00 (1.58, 2.52) \| \| Q4 \| 1.75 (1.38, 2.20) \| 1.56 (1.08, 2.24) \| 2.11 (1.69, 2.64) \| Q4 \| 1.35 (1.06, 1.72) \| 1.39 (0.95, 2.03) \| 1.57 (1.25, 1.97) \| \| PM_2.5_ (µg/m^3^) \|  \|  \|  \| PM_10_ (µg/m^3^) \|  \|  \|  \| \| Q1 \| Reference \| 1.15 (0.79, 1.67) \| 1.42 (1.10, 1.83) \| Q1 \| Reference \| 1.41 (0.97, 2.06) \| 1.51 (1.15, 1.98) \| \| Q2 \| 1.46 (1.16, 1.82) \| 1.45 (1.03, 2.04) \| 2.08 (1.64, 2.66) \| Q2 \| 1.56 (1.23, 1.97) \| 1.62 (1.14, 2.32) \| 2.02 (1.57, 2.61) \| \| Q3 \| 0.85 (0.66, 1.08) \| 1.28 (0.88, 1.85) \| 1.30 (1.01, 1.68) \| Q3 \| 1.64 (1.29, 2.07) \| 1.91 (1.31, 2.78) \| 2.28 (1.77, 2.93) \| \| Q4 \| 1.93 (1.52, 2.44) \| 1.87 (1.30, 2.68) \| 2.31 (1.84, 2.89) \| Q4 \| 2.25 (1.76, 2.87) \| 2.16 (1.50, 3.10) \| 2.93 (2.32, 3.69) \| \| SO_2_ (µg/m^3^) \|  \|  \|  \| AQI* \|  \|  \|  \| \| Q1 \| Reference \| 1.21 (0.87, 1.70) \| 1.43 (1.12, 1.83) \| Q1 \| Reference \| 1.14 (0.80, 1.62) \| 1.52 (1.18, 1.95) \| \| Q2 \| 0.75 (0.59, 0.96) \| 0.91 (0.62, 1.34) \| 0.97 (0.75, 1.26) \| Q2 \| 0.79 (0.63, 0.99) \| 0.80 (0.54, 1.20) \| 1.24 (0.97, 1.59) \| \| Q3 \| 1.35 (1.08, 1.68) \| 1.58 (1.08, 2.31) \| 2.09 (1.65, 2.64) \| Q3 \| 1.22 (0.97, 1.52) \| 1.67 (1.18, 2.37) \| 1.56 (1.22, 1.98) \| \| Q4 \| 1.59 (1.25, 2.02) \| 1.45 (1.01, 2.07) \| 1.99 (1.59, 2.49) \| Q4 \| 1.62 (1.28, 2.05) \| 1.49 (1.04, 2.13) \| 2.00 (1.60, 2.49) \|   All model adjusted for age, education, residence, marital status, household expenses per capita, smoke, drink, solid fuel usage, sleep duration, depressive symptoms, and non-communicable diseases. ^a^ Based on the WHO Guidelines on Physical Activity and Sedentary Behaviour 2020, inactive was considered as not participating MVPA. And we use the same METs as 300 minutes of moderate physical activity or 150 minutes of vigorous physical activity as the dividing line between insufficiently active and physically active. ^b^ The AQI is a numerical indicator used to assess air quality, based on the concentrations of multiple pollutants, including CO, PM_2.5_, SO_2_, NO_2_, O_3_, and PM_10_. CVD, cardiovascular diseases; MVPA, moderate-vigorous physical activity; CI, confidence interval; CO, Carbon monoxide; PM_1_, particulate matter with aerodynamic diameter ≤1µm; PM_2.5_, particulate matter with aerodynamic diameter ≤2.5µm; SO_2_, Sulfur dioxide; NO_2_, nitrogen dioxide; O_3_, Ozone; PM_10_, particulate matter with aerodynamic diameter ≤10µm; AQI, air quality index. | |

| Supplemental table 45. Joint association of MVPA and air pollutants with the prevalence of CVD in males, CHARLS 2018 (Individual weights were further adjusted). | |
| --- | --- |
| \|  \| Odd Ratio (95%CI) \| \| \|  \| Odd Ratio (95%CI) \| \| \| \| --- \| --- \| --- \| --- \| --- \| --- \| --- \| --- \| \|  \| Physically active^&^ \| Insufficiently active^&^ \| Inactive^&^ \|  \| Physically active^&^ \| Insufficiently active^&^ \| Inactive^&^ \| \| CO (µg/m^3^) \|  \|  \|  \| NO_2_ (µg/m^3^) \|  \|  \|  \| \| Q1 \| Reference \| 1.20 (0.75, 1.90) \| 1.59 (1.21, 2.10) \| Q1 \| Reference \| 0.87 (0.52, 1.44) \| 1.50 (1.15, 1.96) \| \| Q2 \| 0.83 (0.63, 1.09) \| 0.83 (0.49, 1.39) \| 1.09 (0.83, 1.44) \| Q2 \| 0.90 (0.69, 1.17) \| 1.13 (0.72, 1.77) \| 1.34 (1.03, 1.74) \| \| Q3 \| 0.90 (0.69, 1.18) \| 0.93 (0.58, 1.49) \| 1.49 (1.12, 1.97) \| Q3 \| 0.96 (0.74, 1.24) \| 0.95 (0.58, 1.55) \| 1.43 (1.10, 1.86) \| \| Q4 \| 1.54 (1.19, 2.00) \| 2.14 (1.38, 3.31) \| 2.31 (1.78, 2.99) \| Q4 \| 1.21 (0.93, 1.57) \| 1.99 (1.32, 2.99) \| 1.97 (1.53, 2.54) \| \| PM_1_ (µg/m^3^) \|  \|  \|  \| O_3_ (µg/m^3^) \|  \|  \|  \| \| Q1 \| Reference \| 1.21 (0.74, 1.99) \| 1.38 (1.05, 1.83) \| Q1 \| Reference \| 1.25 (0.77, 2.03) \| 1.82 (1.38, 2.41) \| \| Q2 \| 0.96 (0.74, 1.26) \| 0.96 (0.62, 1.51) \| 1.46 (1.12, 1.92) \| Q2 \| 1.51 (1.16, 1.96) \| 1.16 (0.71, 1.89) \| 1.83 (1.39, 2.41) \| \| Q3 \| 0.88 (0.67, 1.15) \| 1.24 (0.76, 2.02) \| 1.54 (1.18, 2.01) \| Q3 \| 1.27 (0.97, 1.67) \| 2.12 (1.38, 3.26) \| 2.11 (1.61, 2.77) \| \| Q4 \| 1.91 (1.48, 2.46) \| 2.17 (1.41, 3.35) \| 2.70 (2.08, 3.49) \| Q4 \| 1.64 (1.26, 2.14) \| 1.98 (1.24, 3.14) \| 2.45 (1.87, 3.20) \| \| PM_2.5_ (µg/m^3^) \|  \|  \|  \| PM_10_ (µg/m^3^) \|  \|  \|  \| \| Q1 \| Reference \| 1.09 (0.65, 1.81) \| 1.27 (0.95, 1.69) \| Q1 \| Reference \| 0.91 (0.54, 1.54) \| 1.30 (0.97, 1.76) \| \| Q2 \| 1.03 (0.79, 1.34) \| 1.01 (0.66, 1.57) \| 1.72 (1.32, 2.23) \| Q2 \| 1.06 (0.81, 1.39) \| 1.35 (0.86, 2.14) \| 1.90 (1.45, 2.47) \| \| Q3 \| 0.69 (0.52, 0.91) \| 1.23 (0.76, 1.99) \| 1.21 (0.92, 1.58) \| Q3 \| 1.04 (0.79, 1.36) \| 1.48 (0.93, 2.34) \| 1.58 (1.19, 2.08) \| \| Q4 \| 1.86 (1.44, 2.39) \| 1.94 (1.26, 3.01) \| 2.56 (1.98, 3.31) \| Q4 \| 2.10 (1.62, 2.73) \| 2.26 (1.46, 3.49) \| 2.94 (2.26, 3.81) \| \| SO_2_ (µg/m^3^) \|  \|  \|  \| AQI* \|  \|  \|  \| \| Q1 \| Reference \| 1.17 (0.74, 1.85) \| 1.50 (1.14, 1.98) \| Q1 \| Reference \| 1.19 (0.73, 1.94) \| 1.43 (1.08, 1.89) \| \| Q2 \| 0.71 (0.53, 0.94) \| 0.87 (0.51, 1.49) \| 1.35 (1.02, 1.78) \| Q2 \| 0.92 (0.70, 1.20) \| 0.68 (0.41, 1.11) \| 1.19 (0.90, 1.58) \| \| Q3 \| 1.34 (1.04, 1.74) \| 1.33 (0.82, 2.16) \| 2.09 (1.59, 2.74) \| Q3 \| 0.84 (0.64, 1.11) \| 1.54 (0.99, 2.42) \| 1.68 (1.28, 2.21) \| \| Q4 \| 1.76 (1.34, 2.30) \| 2.15 (1.42, 3.26) \| 2.20 (1.69, 2.88) \| Q4 \| 1.82 (1.40, 2.36) \| 2.04 (1.32, 3.16) \| 2.48 (1.91, 3.21) \|   All model adjusted for age, education, residence, marital status, household expenses per capita, smoke, drink, solid fuel usage, sleep duration, depressive symptoms, and non-communicable diseases. ^a^ Based on the WHO Guidelines on Physical Activity and Sedentary Behaviour 2020, inactive was considered as not participating MVPA. And we use the same METs as 300 minutes of moderate physical activity or 150 minutes of vigorous physical activity as the dividing line between insufficiently active and physically active. ^b^ The AQI is a numerical indicator used to assess air quality, based on the concentrations of multiple pollutants, including CO, PM_2.5_, SO_2_, NO_2_, O_3_, and PM_10_. CVD, cardiovascular diseases; MVPA, moderate-vigorous physical activity; CI, confidence interval; CO, Carbon monoxide; PM_1_, particulate matter with aerodynamic diameter ≤1µm; PM_2.5_, particulate matter with aerodynamic diameter ≤2.5µm; SO_2_, Sulfur dioxide; NO_2_, nitrogen dioxide; O_3_, Ozone; PM_10_, particulate matter with aerodynamic diameter ≤10µm; AQI, air quality index. |  |

| Supplemental table 46. Subgroup analysis on the associations between air pollutants and the prevalence of CVD stratified by MVPA categories, CHARLS 2018 (Individual weights were further adjusted). |  |
| --- | --- |
| \|  \| MVPA level \| \|  \| \| --- \| --- \| --- \| --- \| \| High levels of air pollutants \| Low-medium \| High \| P for interaction \| \| CO \| 1.58 (1.42, 1.75) \| 1.92 (1.54, 2.41) \| 0.118 \| \| PM_1_ \| 1.68 (1.51, 1.86) \| 1.98 (1.59, 2.48) \| 0.181 \| \| PM_2.5_ \| 1.70 (1.54, 1.89) \| 2.09 (1.67, 2.61) \| 0.101 \| \| SO_2_ \| 1.44 (1.30, 1.61) \| 1.91 (1.52, 2.41) \| 0.029 \| \| NO_2_ \| 1.32 (1.19, 1.46) \| 1.53 (1.21, 1.94) \| 0.244 \| \| O_3_ \| 1.17 (1.05, 1.30) \| 1.30 (1.03, 1.65) \| 0.410 \| \| PM_10_ \| 1.67 (1.51, 1.86) \| 1.93 (1.54, 2.41) \| 0.268 \| \| AQI \| 1.61 (1.45, 1.78) \| 2.00 (1.59, 2.51) \| 0.084 \|   P<0.05 was considered statistically significant. The cut off point of air pollutants and moderate-vigorous physical activity are cohort-specific cut-off points at the 75%. Multivariable logistic regression model was used to examine the associations, which were adjusted for sex, age, education, residence, marital status, household expenses per capita, smoke, drink, solid fuel usage, sleep duration, depressive symptoms, and non-communicable diseases. The results expressed as odd ratios (OR) and 95% confidence intervals (CI). CVD, cardiovascular diseases; MVPA, moderate-vigorous physical activity; CO, Carbon monoxide; PM_1_, particulate matter with aerodynamic diameter ≤1µm; PM_2.5_, particulate matter with aerodynamic diameter ≤2.5µm; SO_2_, Sulfur dioxide; NO_2_, nitrogen dioxide; O_3_, Ozone; PM_10_, particulate matter with aerodynamic diameter ≤10µm; AQI, air quality index. | |

| Supplemental table 47. Subgroup analysis on the associations between air pollutants and the prevalence of CVD stratified by MVPA categories in females, CHARLS 2018 (Individual weights were further adjusted). |  |
| --- | --- |
| \|  \| MVPA level \| \|  \| \| --- \| --- \| --- \| --- \| \| High levels of air pollutants \| Low-medium \| High \| P for interaction \| \| CO \| 1.54 (1.34, 1.77) \| 1.75 (1.25, 2.44) \| 0.485 \| \| PM_1_ \| 1.56 (1.36, 1.79) \| 1.72 (1.23, 2.41) \| 0.587 \| \| PM_2.5_ \| 1.59 (1.38, 1.83) \| 1.87 (1.34, 2.60) \| 0.378 \| \| SO_2_ \| 1.40 (1.21, 1.61) \| 1.72 (1.22, 2.43) \| 0.273 \| \| NO_2_ \| 1.30 (1.13, 1.50) \| 1.40 (0.99, 1.99) \| 0.703 \| \| O_3_ \| 1.08 (0.93, 1.24) \| 1.18 (0.83, 1.68) \| 0.637 \| \| PM_10_ \| 1.57 (1.37, 1.80) \| 1.60 (1.13, 2.26) \| 0.918 \| \| AQI \| 1.51 (1.32, 1.74) \| 1.61 (1.14, 2.27) \| 0.754 \|   P<0.05 was considered statistically significant. The cut off point of air pollutants and moderate-vigorous physical activity are cohort-specific cut-off points at the 75%. Multivariable logistic regression model was used to examine the associations, which were adjusted for age, education, residence, marital status, household expenses per capita, smoke, drink, solid fuel usage, sleep duration, depressive symptoms, and non-communicable diseases. The results expressed as odd ratios (OR) and 95% confidence intervals (CI). CVD, cardiovascular diseases; MVPA, moderate-vigorous physical activity; CO, Carbon monoxide; PM_1_, particulate matter with aerodynamic diameter ≤1µm; PM_2.5_, particulate matter with aerodynamic diameter ≤2.5µm; SO_2_, Sulfur dioxide; NO_2_, nitrogen dioxide; O_3_, Ozone; PM_10_, particulate matter with aerodynamic diameter ≤10µm; AQI, air quality index. | |

| Supplemental table 48. Subgroup analysis on the associations between air pollutants and the prevalence of CVD stratified by MVPA categories in males, CHARLS 2018 (Individual weights were further adjusted). |  |
| --- | --- |
| \|  \| MVPA level \| \|  \| \| --- \| --- \| --- \| --- \| \| High levels of air pollutants \| Low-medium \| High \| P for interaction \| \| CO \| 1.64 (1.40, 1.91) \| 2.30 (1.67, 3.16) \| 0.059 \| \| PM_1_ \| 1.83 (1.56, 2.14) \| 2.56 (1.87, 3.52) \| 0.059 \| \| PM_2.5_ \| 1.85 (1.58, 2.16) \| 2.62 (1.91, 3.58) \| 0.050 \| \| SO_2_ \| 1.48 (1.26, 1.74) \| 2.32 (1.67, 3.21) \| 0.015 \| \| NO_2_ \| 1.37 (1.17, 1.61) \| 1.90 (1.37, 2.64) \| 0.076 \| \| O_3_ \| 1.26 (1.07, 1.48) \| 1.59 (1.14, 2.21) \| 0.215 \| \| PM_10_ \| 1.80 (1.54, 2.11) \| 2.52 (1.84, 3.46) \| 0.060 \| \| AQI \| 1.72 (1.47, 2.02) \| 2.70 (1.97, 3.71) \| 0.012 \|   P<0.05 was considered statistically significant. The cut off point of air pollutants and moderate-vigorous physical activity are cohort-specific cut-off points at the 75%. Multivariable logistic regression model was used to examine the associations, which were adjusted for age, education, residence, marital status, household expenses per capita, smoke, drink, solid fuel usage, sleep duration, depressive symptoms, and non-communicable diseases. The results expressed as odd ratios (OR) and 95% confidence intervals (CI). CVD, cardiovascular diseases; MVPA, moderate-vigorous physical activity; CO, Carbon monoxide; PM_1_, particulate matter with aerodynamic diameter ≤1µm; PM_2.5_, particulate matter with aerodynamic diameter ≤2.5µm; SO_2_, Sulfur dioxide; NO_2_, nitrogen dioxide; O_3_, Ozone; PM_10_, particulate matter with aerodynamic diameter ≤10µm; AQI, air quality index. | |

| Supplemental table 49. Dose–response associations of MVPA with the prevalence of CVD stratified by air pollutants concentration, CHARLS 2018 (Individual weights were further adjusted). |
| --- |
| \| Air pollutants \| P for overall (low-medium) \| P for nonlinear (low-medium) \| P for overall (high) \| P for nonlinear (high) \| P for interaction \| \| --- \| --- \| --- \| --- \| --- \| --- \| \| Duration \|  \|  \|  \|  \|  \| \| CO \| <0.001 \| 0.006 \| 0.005 \| 0.078 \| 0.282 \| \| PM_1_ \| <0.001 \| 0.012 \| 0.008 \| 0.096 \| 0.156 \| \| PM_2.5_ \| <0.001 \| 0.017 \| 0.010 \| 0.062 \| 0.069 \| \| SO_2_ \| <0.001 \| 0.035 \| 0.010 \| 0.013 \| 0.004 \| \| NO_2_ \| <0.001 \| 0.013 \| 0.003 \| 0.121 \| 0.379 \| \| O_3_ \| <0.001 \| 0.010 \| 0.007 \| 0.133 \| 0.179 \| \| PM_10_ \| <0.001 \| 0.015 \| 0.008 \| 0.074 \| 0.277 \| \| AQI \| <0.001 \| 0.011 \| 0.022 \| 0.078 \| 0.070 \| \|  \|  \|  \|  \|  \|  \| \| METs \|  \|  \|  \|  \|  \| \| CO \| <0.001 \| 0.002 \| 0.002 \| 0.012 \| 0.107 \| \| PM_1_ \| <0.001 \| 0.002 \| 0.004 \| 0.030 \| 0.105 \| \| PM_2.5_ \| <0.001 \| 0.003 \| 0.005 \| 0.015 \| 0.034 \| \| SO_2_ \| <0.001 \| 0.006 \| 0.005 \| 0.006 \| 0.004 \| \| NO_2_ \| <0.001 \| 0.003 \| 0.001 \| 0.017 \| 0.134 \| \| O_3_ \| <0.001 \| 0.001 \| 0.003 \| 0.064 \| 0.179 \| \| PM_10_ \| <0.001 \| 0.004 \| 0.003 \| 0.010 \| 0.100 \| \| AQI \| <0.001 \| 0.002 \| 0.008 \| 0.015 \| 0.033 \| |
| \| P<0.05 was considered statistically significant. The cut off point of air pollutants and moderate-vigorous physical activity are cohort-specific cut-off points at the 75%. All models were adjusted for was used to examine the associations, which were adjusted for sex, age, education, residence, marital status, household expenses per capita, smoke, drink, solid fuel usage, sleep duration, depressive symptoms, and non-communicable diseases. CVD, cardiovascular diseases; MVPA, moderate-vigorous physical activity; METs, Metabolic equivalents; CO, Carbon monoxide; PM_1_, particulate matter with aerodynamic diameter ≤1µm; PM_2.5_, particulate matter with aerodynamic diameter ≤2.5µm; SO_2_, Sulfur dioxide; NO_2_, nitrogen dioxide; O_3_, Ozone; PM_10_, particulate matter with aerodynamic diameter ≤10µm; AQI, air quality index. \| \| --- \| |
|  |

|  |
| --- |
| Supplemental table 50. Dose–response associations of MVPA with the prevalence of CVD stratified by air pollutants concentration in females, CHARLS 2018 (Individual weights were further adjusted). |
| \| Air pollutants \| P for overall (low-medium) \| P for nonlinear (low-medium) \| P for overall (high) \| P for nonlinear (high) \| P for interaction \| \| --- \| --- \| --- \| --- \| --- \| --- \| \| Duration \|  \|  \|  \|  \|  \| \| CO \| <0.001 \| 0.542 \| 0.340 \| 0.456 \| 0.199 \| \| PM_1_ \| <0.001 \| 0.469 \| 0.291 \| 0.391 \| 0.210 \| \| PM_2.5_ \| <0.001 \| 0.509 \| 0.332 \| 0.327 \| 0.108 \| \| SO_2_ \| <0.001 \| 0.396 \| 0.183 \| 0.104 \| 0.029 \| \| NO_2_ \| <0.001 \| 0.130 \| 0.313 \| 0.721 \| 0.304 \| \| O_3_ \| <0.001 \| 0.147 \| 0.268 \| 0.425 \| 0.086 \| \| PM_10_ \| <0.001 \| 0.520 \| 0.183 \| 0.240 \| 0.336 \| \| AQI \| <0.001 \| 0.447 \| 0.340 \| 0.332 \| 0.209 \| \|  \|  \|  \|  \|  \|  \| \| METs \|  \|  \|  \|  \|  \| \| CO \| <0.001 \| 0.277 \| 0.221 \| 0.128 \| 0.060 \| \| PM_1_ \| <0.001 \| 0.250 \| 0.175 \| 0.110 \| 0.121 \| \| PM_2.5_ \| <0.001 \| 0.317 \| 0.181 \| 0.089 \| 0.061 \| \| SO_2_ \| <0.001 \| 0.296 \| 0.049 \| 0.015 \| 0.006 \| \| NO_2_ \| <0.001 \| 0.058 \| 0.144 \| 0.115 \| 0.119 \| \| O_3_ \| <0.001 \| 0.080 \| 0.224 \| 0.206 \| 0.068 \| \| PM_10_ \| <0.001 \| 0.381 \| 0.057 \| 0.025 \| 0.090 \| \| AQI \| <0.001 \| 0.261 \| 0.143 \| 0.063 \| 0.087 \| |
| \| P<0.05 was considered statistically significant. The cut off point of air pollutants and moderate-vigorous physical activity are cohort-specific cut-off points at the 75%. All models were adjusted for was used to examine the associations, which were adjusted for age, education, residence, marital status, household expenses per capita, smoke, drink, solid fuel usage, sleep duration, depressive symptoms, and non-communicable diseases. CVD, cardiovascular diseases; MVPA, moderate-vigorous physical activity; METs, Metabolic equivalents; CO, Carbon monoxide; PM_1_, particulate matter with aerodynamic diameter ≤1µm; PM_2.5_, particulate matter with aerodynamic diameter ≤2.5µm; SO_2_, Sulfur dioxide; NO_2_, nitrogen dioxide; O_3_, Ozone; PM_10_, particulate matter with aerodynamic diameter ≤10µm; AQI, air quality index. \| \| --- \| |

| Supplemental table 51. Dose–response associations of MVPA with the prevalence of CVD stratified by air pollutants concentration in males, CHARLS 2018 (Individual weights were further adjusted).   \| Air pollutants \| P for overall (low-medium) \| P for nonlinear (low-medium) \| P for overall (high) \| P for nonlinear (high) \| P for interaction \| \| --- \| --- \| --- \| --- \| --- \| --- \| \| Duration \|  \|  \|  \|  \|  \| \| CO \| <0.001 \| 0.002 \| 0.011 \| 0.101 \| 0.850 \| \| PM_1_ \| <0.001 \| 0.003 \| 0.036 \| 0.116 \| 0.445 \| \| PM_2.5_ \| <0.001 \| 0.002 \| 0.026 \| 0.114 \| 0.470 \| \| SO_2_ \| <0.001 \| 0.005 \| 0.055 \| 0.072 \| 0.101 \| \| NO_2_ \| <0.001 \| 0.007 \| 0.005 \| 0.080 \| 0.801 \| \| O_3_ \| <0.001 \| 0.002 \| 0.025 \| 0.206 \| 0.853 \| \| PM_10_ \| <0.001 \| 0.001 \| 0.054 \| 0.212 \| 0.460 \| \| AQI \| <0.001 \| 0.002 \| 0.071 \| 0.157 \| 0.302 \| \|  \|  \|  \|  \|  \|  \| \| METs \|  \|  \|  \|  \|  \| \| CO \| <0.001 \| 0.002 \| 0.005 \| 0.051 \| 0.780 \| \| PM_1_ \| <0.001 \| 0.001 \| 0.021 \| 0.084 \| 0.424 \| \| PM_2.5_ \| <0.001 \| 0.002 \| 0.015 \| 0.075 \| 0.439 \| \| SO_2_ \| <0.001 \| 0.002 \| 0.035 \| 0.113 \| 0.199 \| \| NO_2_ \| <0.001 \| 0.004 \| 0.003 \| 0.067 \| 0.612 \| \| O_3_ \| <0.001 \| 0.002 \| 0.009 \| 0.174 \| 0.942 \| \| PM_10_ \| <0.001 \| 0.001 \| 0.038 \| 0.153 \| 0.496 \| \| AQI \| <0.001 \| 0.001 \| 0.047 \| 0.114 \| 0.316 \| |
| --- | --- | --- | --- | --- | --- | --- | --- | --- | --- | --- | --- | --- | --- | --- | --- | --- | --- | --- | --- | --- | --- | --- | --- | --- | --- | --- | --- | --- | --- | --- | --- | --- | --- | --- | --- | --- | --- | --- | --- | --- | --- | --- | --- | --- | --- | --- | --- | --- | --- | --- | --- | --- | --- | --- | --- | --- | --- | --- | --- | --- | --- | --- | --- | --- | --- | --- | --- | --- | --- | --- | --- | --- | --- | --- | --- | --- | --- | --- | --- | --- | --- | --- | --- | --- | --- | --- | --- | --- | --- | --- | --- | --- | --- | --- | --- | --- | --- | --- | --- | --- | --- | --- | --- | --- | --- | --- | --- | --- | --- | --- | --- | --- | --- | --- | --- | --- | --- | --- | --- | --- |
| P<0.05 was considered statistically significant. The cut off point of air pollutants and moderate-vigorous physical activity are cohort-specific cut-off points at the 75%. All models were adjusted for was used to examine the associations, which were adjusted for age, education, residence, marital status, household expenses per capita, smoke, drink, solid fuel usage, sleep duration, depressive symptoms, and non-communicable diseases. CVD, cardiovascular diseases; MVPA, moderate-vigorous physical activity; METs, Metabolic equivalents; CO, Carbon monoxide; PM_1_, particulate matter with aerodynamic diameter ≤1µm; PM_2.5_, particulate matter with aerodynamic diameter ≤2.5µm; SO_2_, Sulfur dioxide; NO_2_, nitrogen dioxide; O_3_, Ozone; PM_10_, particulate matter with aerodynamic diameter ≤10µm; AQI, air quality index. |

| Supplemental table 52. Path data of the example in which MVPA is hypothesized as a mediator of the relation between air pollutants and the prevalence of CVD, CHARLS 2018 (Individual weights were further adjusted). | | | |  |
| --- | --- | --- | --- | --- |
| Air pollution | ADE (average) | ACME (average) | Proportion of mediation  (average) |  |
| CO | 0.070860*** | 0.008710*** | 10.88%*** |  |
| PM_1_ | 0.002108*** | 0.000280*** | 11.68%*** |  |
| PM_2.5_ | 0.001464*** | 0.000158*** | 9.68%*** |  |
| SO_2_ | 0.002922*** | 0.000322*** | 9.90%*** |  |
| NO_2_ | 0.001156*** | 0.000246*** | 17.45%*** |  |
| O_3_ | 0.000860*** | 0.000145*** | 13.90%*** |  |
| PM_10_ | 0.000974*** | 0.000073*** | 6.94%*** |  |
| AQI | 0.000950*** | 0.000092*** | 8.80%*** |  |
| *** p<0.001, ** p<0.01, * p<0.05. Multivariable logistic regression model was used to examine the associations, which were adjusted for sex, age, education, residence, marital status, household expenses per capita, smoke, drink, solid fuel usage, sleep duration, depressive symptoms, and non-communicable diseases. CVD, cardiovascular diseases; MVPA, moderate-vigorous physical activity; CO, Carbon monoxide; PM_1_, particulate matter with aerodynamic diameter ≤1µm; PM_2.5_, particulate matter with aerodynamic diameter ≤2.5µm; SO_2_, Sulfur dioxide; NO_2_, nitrogen dioxide; O_3_, Ozone; PM_10_, particulate matter with aerodynamic diameter ≤10µm; AQI, air quality index; ACME, average causal mediation effects; ADE, average direct effects. | | | | |

| Supplemental table 53. Path data of the example in which MVPA is hypothesized as a mediator of the relation between air pollutants and the prevalence of CVD in females, CHARLS 2018 (Individual weights were further adjusted). | | | | |
| --- | --- | --- | --- | --- |
| Air pollution | ADE (average) | ACME (average) | Proportion of mediation  (average) | |
| CO | 0.052790** | 0.011890*** | 18.14%** | |
| PM_1_ | 0.001535** | 0.000392*** | 19.95%** | |
| PM_2.5_ | 0.001318*** | 0.000211*** | 13.57%*** | |
| SO_2_ | 0.002665*** | 0.000409*** | 13.19%*** | |
| NO_2_ | 0.000660 | 0.000342*** | 31.60% | |
| O_3_ | 0.000686* | 0.000245*** | 24.30%** | |
| PM_10_ | 0.000991*** | 0.000095*** | 8.62%*** | |
| AQI | 0.000931*** | 0.000119*** | 11.10%*** | |
| *** p<0.001, ** p<0.01, * p<0.05. Multivariable logistic regression model was used to examine the associations, which were adjusted for age, education, residence, marital status, household expenses per capita, smoke, drink, solid fuel usage, sleep duration, depressive symptoms, and non-communicable diseases. CVD, cardiovascular diseases; MVPA, moderate-vigorous physical activity; CO, Carbon monoxide; PM_1_, particulate matter with aerodynamic diameter ≤1µm; PM_2.5_, particulate matter with aerodynamic diameter ≤2.5µm; SO_2_, Sulfur dioxide; NO_2_, nitrogen dioxide; O_3_, Ozone; PM_10_, particulate matter with aerodynamic diameter ≤10µm; AQI, air quality index; ACME, average causal mediation effects; ADE, average direct effects. | | | |  |

| Supplemental table 54. Path data of the example in which MVPA is hypothesized as a mediator of the relation between air pollutants and the prevalence of CVD in males, CHARLS 2018 (Individual weights were further adjusted). | | | | |
| --- | --- | --- | --- | --- |
| Air pollution | ADE (average) | ACME (average) | Proportion of mediation  (average) | |
| CO | 0.088960*** | 0.005370*** | 5.54%*** | |
| PM_1_ | 0.002590*** | 0.000168*** | 5.96%*** | |
| PM_2.5_ | 0.001580*** | 0.000102*** | 5.89%*** | |
| SO_2_ | 0.003085*** | 0.000231*** | 6.87%*** | |
| NO_2_ | 0.001610** | 0.000148*** | 8.17%** | |
| O_3_ | 0.000857*** | 0.000064*** | 6.42%*** | |
| PM_10_ | 0.000955*** | 0.000049*** | 4.74%*** | |
| AQI | 0.000946*** | 0.000064*** | 6.17%*** | |
| *** p<0.001, ** p<0.01, * p<0.05. Multivariable logistic regression model was used to examine the associations, which were adjusted for age, education, residence, marital status, household expenses per capita, smoke, drink, solid fuel usage, sleep duration, depressive symptoms, and non-communicable diseases. CVD, cardiovascular diseases; MVPA, moderate-vigorous physical activity; CO, Carbon monoxide; PM_1_, particulate matter with aerodynamic diameter ≤1µm; PM_2.5_, particulate matter with aerodynamic diameter ≤2.5µm; SO_2_, Sulfur dioxide; NO_2_, nitrogen dioxide; O_3_, Ozone; PM_10_, particulate matter with aerodynamic diameter ≤10µm; AQI, air quality index; ACME, average causal mediation effects; ADE, average direct effects. | | | |  |
